# Synthesis and Anti-Cancer Activity In Vitro of Synephrine Derivatives

**DOI:** 10.3390/biom15010002

**Published:** 2024-12-24

**Authors:** Ekaterina M. Zhidkova, Evgeniya S. Oleynik, Ekaterina A. Mikhina, Daria V. Stepanycheva, Diana D. Grigoreva, Lyubov E. Grebenkina, Kirill V. Gordeev, Ekaterina D. Savina, Andrey V. Matveev, Marianna G. Yakubovskaya, Ekaterina A. Lesovaya

**Affiliations:** 1 Department of Chemical Carcinogenesis, Institute of Carcinogenesis, N.N. Blokhin National Medical Research Center for Oncology, Kashirskoe Shosse 24-15, Moscow 115478, Russia; zhidkova_em@mail.ru (E.M.Z.); darya.stepanycheva@yandex.ru (D.V.S.); grigodidmit@gmail.com (D.D.G.); mgyakubovskaya@mail.ru (M.G.Y.); 2 Department of Biotechnology and Industrial Pharmacy, Lomonosov Institute of Fine Chemical Technologies, MIREA—Russian Technological University, 86 Vernadsky Prospekt, Moscow 119571, Russia; oleynik_evgenia@mail.ru (E.S.O.); mik.hi@mail.ru (E.A.M.); legrebenkina@mail.ru (L.E.G.); katy.dm.savina@gmail.com (E.D.S.); 4motya@gmail.com (A.V.M.); 3 Faculty of Pharmacy, Kuban State Medical University, Ministry of Health of Russia, 4 Mitrofan Sedin Str., Krasnodar 350063, Russia; gordeev.kirill.loremipsum@gmail.com; 4 Institute of Medicine, Peoples’ Friendship University of Russia, Miklukho-Maklaya St. 6, Moscow 117198, Russia; 5 Faculty of Oncology, I.P. Pavlov Ryazan State Medical University, Vysokovol’tnaya Str. 9, Ryazan 390026, Russia

**Keywords:** selective glucocorticoid receptor agonists, synephrine derivative synthesis, virtual docking, cytotoxicity, hematological malignancies

## Abstract

Glucocorticoids (GCs) are routinely used to treat hematological malignancies; however, long-term treatment with GCs can lead to atrophic and metabolic adverse effects. Selective glucocorticoid receptor agonists (SEGRAs) with reduced side effects may act as a superior alternative to GCs. More than 30 SEGRAs have been described so far, yet none of them reached clinical trials for anti-cancer treatment. In the present work, we propose a novel approach to increase the number of potential SEGRAs by obtaining derivatives of synephrine, a molecule of natural origin. We synthesized 26 novel compounds from the class of synephrine derivatives and characterized them by HRMS, and ^1^H and ^13^C NMR. We evaluated *in vitro* anti-cancer effects in leukemia K562 and lymphoma Granta cells using the MTT assay and studied their potential affinity for the glucocorticoid receptor (GR) *in silico* using the molecular docking approach. The novel derivative 1-[4-(benzyloxy)phenyl]-2-(hexylamino)ethanol (**10S-E2**) with the highest GR affinity *in silico* exhibited cytotoxic activity against K562 and Granta cells after 24 h of treatment at the concentration of approximately 13 µM which correlated with its highest MolDock Score. The other compound with high GR affinity, 2-(hexylamino)-1-(4-nitrophenyl)ethanol (**13S-G2**), demonstrated cytotoxicity in both cell lines at concentrations of 50–70 µM. Overall, our results may provide a solid rationale for developing and further investigating synephrine derivatives as SEGRAs with anti-cancer activity.

## 1. Introduction

Glucocorticoids (GCs) have been remarkably popular for treating autoimmune and inflammatory diseases (rheumatoid arthritis, psoriasis, atopic dermatitis, asthma) as well as cancer for the last 75 years. Unfortunately, in most cases, chronic GC administration induces severe adverse effects including osteoporosis, steroid diabetes, water–salt imbalance, lipodystrophy, and other metabolic complications [1,2]. The biological effects of GCs depend on activating the glucocorticoid receptor (GR), a well-known transcription factor regulating gene expression through two distinct mechanisms. Therapeutic effects of GCs arise from DNA-independent transcriptional repression (TR) mediated by protein–protein interactions between the GR and other transcription factors that inhibit cancer cell survival. The side effects of GCs are associated with transcriptional activation (TA) that requires GR dimerization and dimer binding to the glucocorticoid-responsive elements in the promoters of pro-inflammatory and anti-apoptotic genes [2,3,4].

Since GCs exhibit specific cytotoxic action towards immune cells, they are routinely used to treat hematological malignancies: acute and chronic leukemia as well lymphomas of different origins. Blood malignancies have been among the most common cancers in 2024, and the number of the patients is increasing; 400,000 patients with chronic myeloid leukemia are expected in Europe by 2050 [5]. Mantle cell lymphoma is a subtype of non-Hodgkin’s lymphoma accounting for 5–7% of all NHL cases associated with a poor long-term prognosis [6]. GCs still remain the first-line therapy and are often combined with conventional and novel agents for combination chemotherapy [7,8,9].

**Figure 1:**
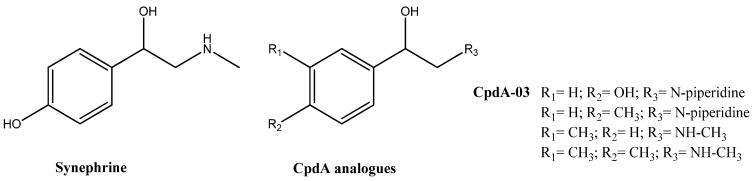
Structure of synephrine and described SEGRAs.

The approaches to increase GC therapeutic activity and decrease their side effects include devising selective glucocorticoid receptor agonists (SEGRAs) that activate only GR TR [4,10,11]. Over the last decades, more than 30 SEGRAs of natural and synthetic origin have been described [4,12]. Our group and others have previously described the low-molecular-weight compound 2-(4-acetoxyphenyl)-2-chloro-N-methylammonium chloride, or CpdA, isolated from the African shrub *Salsola tuberculatiformis Botschantzev*, which acts as a SEGRA. CpdA selectively induced GR TR and exhibited the anti-cancer effects in models of blood and solid cancers, both *in vitro* and *in vivo*, either individually or in tandem with other anti-cancer drugs (Bortezomib, MLN4924 and some others) [4,9,13,14]. However, CpdA use in clinics is limited due to its chemical instability and decomposition to the intermediate metabolite aziridine, a well-known carcinogen [15,16]. Therefore, we designed CpdA derivatives to invent novel SEGRAs (Figure 1). 4-(1-hydroxy-2-(piperidin-1-yl)ethyl)phenol, or CpdA-03, demonstrated superior GR affinity and stability compared to CpdA as well as anti-lymphoma properties both *in vitro* and *in vivo* [17].

In the present work, we aim to evaluate a new synthetic strategy for SEGRA development with the synephrine molecule used as a template instead of CpdA. Synephrine is the final product of CpdA metabolism, which serves as a CpdA precursor [16,18,19,20]. Synephrine derivatives may prove to be more beneficial for clinical practice as compared to the GR ligands, the selective glucocorticoid receptor agonist CpdA, which is structurally similar to synephrine but less stable as stated above. First, we designed new strategies to synthesize novel synephrine derivatives that could act as potential SEGRAs. Second, we performed *in silico* and *in vitro* screening of their biological activity to select the hit compounds for further investigation.

## 2. Materials and Methods

### 2.1. Chemistry

#### 2.1.1. General Procedures and Reagents

All reagents were obtained from commercial sources (Merck KGaA, Darmstadt, Germany) and used without additional purification. Deuterated solvents were purchased from Cambridge Isotope Laboratories, Inc. (Tewkesbury, MA, USA). Silica gel 60 (Merck KGaA, Darmstadt, Germany) was used for column chromatography. Analytical thin layaer chromatography (TLC) was performed on Sorbfil PTX-AF-A-UV silica gel plates (Imid Co., Ltd., Moscow, Russia).

Solvents were removed under reduced pressure on a Rotavapor R-200 rotary vacuum evaporator using a vacuum water jet pump (Buchi, Flawil, Switzerland). ^1^H and ^13^C NMR spectra were recorded on a Bruker DPX-300 instrument (300 and 75 MHz, respectively). Chemical shifts are in parts per million (ppm, δ) relative to the solvents CDCl_3_ (7.25 ppm), DMSO-d_6_ (2.49 ppm), and CD_3_OD (4.78 ppm) for ^1^H nuclear magnetic resonance (NMR) and CDCl_3_ (77.2 ppm), DMSO-d_6_ (39.5 ppm), and CD_3_OD (49.0 ppm) for ^13^C NMR. The data are presented as follows: chemical shifts, multiplicity (s—singlet, br.s—broad singlet, m—multiplet), and the relative value of integration. The determination of solvent peaks was carried out following the literature data [21]. The spectra can be found in Supplementary Figures S1–S52. High-resolution mass spectra (HRMS) were recorded on an Agilent 6224 using electron sputtering ionization, ESI (Agilent Technologies, Santa Clara, CA, USA). HPLC–MS measurements were performed on the Agilent InfinityLab LC/MSD iQ liquid chromatograph with the Agilent Single Quadrupole LC/MSD iQ mass spectrometric detector (Agilent Technologies, Santa Clara, CA, USA). The separation was performed on the Agilent Poroshell 300SB C18 column (Agilent Technologies, Santa Clara, CA, USA), 2.1 × 75 mm. Eluent: 0.1% acetonitrile in deionized water with 0.1% trifluoroacetic acid, with consumption of 0.5 mL per minute.

#### 2.1.2. General Procedure for the Synthesis of Compounds **1A**,**B**,**C**

Bromoacetophenones **1A**,**B**,**C** were obtained by α-bromination of the corresponding commercially available acetophenones according to the method [22] with comparable yields.

#### 2.1.3. General Procedure for the Synthesis of Products **3S-C1**, **6S-C4**, **7S-E1**, and **11S-E4**

A solution of bromoacetophenone **1** in methylene chloride (1 mL of solvent per 100 mg **1**) were added dropwise to a solution of amine in methylene chloride (10 mL of solvent per 1 mL of amine). The reaction was monitored by the initial bromoacetophenone **1** conversion by TLC. After bromoacetophenone **1** conversion was complete, volatile components were evaporated. The residue was dissolved in 5 mL of water and extracted with methylene chloride (3 × 10 mL). The organic layers were combined and dried with anhydrous calcium chloride, filtered off, and evaporated. The resulting oil was dissolved in 10 mL of methanol, and NaBH_4_ was added in portions during cooling. After the intermediate conversion (monitored by TLC), the reaction mixture was acidified with 10% aqueous HCl solution to pH 3, and the volatile components were evaporated. The extraction and drying processes were repeated. The products **3S-C1**, **6S-C4**, **7S-E1**, and **11S-E4** were isolated by column chromatography (methanol gradient from 0 to 15%).

*1-(4-methoxyphenyl)-2-piperedine-1-yl ethanol (* **3S-C1** *)*

Reaction mixture composition: 0.63 mL (6.30 mmol) piperidine, 0.42 g (1.80 mmol) of 4-methoxybromoacetophenone **1A**, and 0.07 g (1.80 mmol) NaBH_4_. Product yield was 0.31 g (74%). R_f_ = 0.45 (10% methanol in chloroform). Based on HPLC data, the content of the target substance was 97.5%. ^1^H NMR spectrum (CDCl_3_) δ: 7.31–7.28 (m, 2H, Ph); 6.83–6.80 (m, 2H, Ph); 5.33–5.30 (m, 1H, -CH); 4.60 (br. s, 1H, -OH); 3.76–3.69 (m, 2H,-CH_2_-OH); 3.74 (s, 1H, -O-CH_3_); 3.19–2.99 (m, 2H, piperidine -CH_2_-); 2.83–2.76 (m, 2H, piperidine -CH_2_-); 2.29–2.16 (m, 2H, piperidine -CH_2_-); 1.83–1.77 (m, 3H, piperidine 2×-CH_2_-); 1.47–1.37 (m, 1H, piperidine -CH_2_-). ^13^C NMR spectrum (CDCl_3_) δ: 159.42; 132.18; 127.15; 114.03; 67.43; 65.52; 55.51; 55.26; 54.04; 22.72; 22.67; 21.80. For C_14_H_21_NO_2_, the calculated and experimental [M+H]^+^ values were 236.1650 and 236.1652, respectively.

*2-(2-hydroxyethyl)(methyl) amino)-1-(4-methoxyphenyl)ethanol (* **6S-C4** *)*

Reaction mixture composition: 0.55 mL (7.00 mmol) of N-methyl ethanolamine, 0.45 g (2.00 mmol) of 4-methoxybromoacetophenone **1A**, and 0.08 g (2.00 mmol) of NaBH_4_. Product yield was 0.20 g (45%). R_f_ = 0.75 (5% methanol in chloroform). The content of the target substance was 99% (based on HPLC). ^1^H NMR spectrum (CDCl_3_) δ: 7.28–7.25 (m, 2H, Ph); 6.87–6.84 (m, 2H, Ph); 4.53–4.49 (m, 1H, -CH-); 3.99–3.84 (m, 2H, -CH_2_-OH); 3.77 (s, 3H,-O-CH_3_); 2.88–2.72 (m, 2H, -N(CH_3_)-CH_2_-); 2.32 (s, 3H, -N-CH_3_); 2.28–2.06 (m, 2H, -CH(OH)-CH_2_-). ^13^C NMR-spectrum (CDCl_3_) δ: 159.11; 132.16; 127.34; 113.61; 77.48; 66.74; 61.86; 55.15; 54.46; 45.92. For C_12_H_19_NO_3_, the calculated and experimental [M+H]^+^ values were 226.1443 and 226.1445, respectively.

*1-(4-(benzyloxy) phenyl)-2-piperidine-1-yl-ethanol (* **7S-E1** *)*

Reaction mixture composition: 0.63 mL (6.35 mmol) of piperidine, 0.39 g (1.27 mmol) of 4-benzyloxybromoacetophenone **1B**, and 0.05 g (1.27 mmol) of NaBH_4_. Product yield was **7S-E1** was 0.30 g (76%). R_f_ = 0.45 (5% methanol in chloroform). The content of the target substance was 98% (based on HPLC). ^1^H NMR spectrum (CDCl_3_) δ: 7.43–7.27 (m, 7H, Ph); 6.96–6.93 (m, 2H, Ph); 5.04 (s, 2H, -CH_2_-O-); 4.91–4.86 (m, 1H, -CH-OH); 4.62 (br.s., 1H, -OH); 2.86 (br.s., 2H, -CH_2_-); 2.71–2.59 (m, 4H, piperidine 2×CH_2_); 1.78–1.74 (m, 4H, piperidine 2×CH_2_); 1.56–1.50 (m, 2H, piperidine CH_2_). ^13^C NMR spectrum (CDCl_3_) δ: 158.31; 136.89; 133.80; 128.54; 127.42; 127.13; 114.77; 69.96; 67.96; 66.57; 54.62; 24.94; 23.42. For C_20_H_25_NO_2_, the calculated and experimental [M+H]^+^ values were 312.1963 and 312.1965, respectively.

*1-(4-(benzyloxy)phenyl)-2-((2-hydroxyethyl)(methyl)amino)ethanol (* **11S-E4** *)*

Reaction mixture composition: 0.39 mL (4.9 mmol) of N-methyl ethanolamine, 0.30 g (0.98 mmol) of 4-benzyloxybromoacetophenone **1B**, and 0.04 g (0.98 mmol) of NaBH_4_. Product yield was 0.18 g (61%). R_f_ = 0.55 (7% methanol in chloroform). The content of the target substance was 98% (based on HPLC). ^1^H NMR spectrum (CDCl_3_) δ: 7.43–7.27 (m, 7H, Ph); 6.96–6.93 (m, 2H, Ph); 5.04 (s, 2H, -O-CH_2_-); 4.91–4.86 (m, 1H, -CH(OH)-); 4.63 (br.s., 1H, -OH); 2.87 (br.s., 2H, -CH(OH)-CH_2_-) 2.71–2.59 (m, 2H, -N(CH_3_)-CH_2_-); 1.76 (s, 3H, -N-CH_3_); 1.54–1.52 (m, 2H, -CH_2_-OH). ^13^C NMR spectrum (CDCl_3_) δ: 158.93; 136.88; 130.18; 128.60; 128.02; 127.44; 123.29; 114.94; 70.15; 68.97; 61.42; 58.63; 55.24; 37.62. For C_18_H_23_NO_3_, the calculated and experimental [M+H]^+^ values were 302.1756 and 302.1760, respectively.

#### 2.1.4. General Procedure for the Synthesis of the Compounds **4S-C2**, **5S-C3**, **18S-C5**, **19S-C6**, **10S-E2**, **8S-E3**, **20S-E5**, **21S-E6**, **13S-G2**, **14S-G3**, **22S-G5**, **23S-G6**, and **9S-G1**

To a solution of bromoacetophenone **1** in methanol (for **4S-C2**, **5S-C3**, **18S-C5**, **19S-C6**, **10S-E2**, **8S-E3**, **20S-E5**, and **21S-E6**) or 1,4-dioxane (for **13S-G2**, **14S-G3**, **22S-G5**, **23S-G6**, and **9S-G1**) (1 mL per 100 mg) cooled in an ice bath (or at room temperature in the case of **9S-G1** compounds), 1 equivalent (eq) of NaBH_4_ was added in portions. After the conversion of bromoacetophenone **1** (monitored with TLC), a solution of 5 eq of a primary amine and 1.2 eq of KOH in methanol (1 mL per 1 mL amine) was added. After 12 h incubation with stirring, the reaction mixture was acidified with 10% aqueous solution of HCl to pH 3, the volatile components were removed under reduced pressure, and the residue was suspended in 5 mL of water and extracted with methylene chloride (3 × 10 mL). The organic layers were combined and dried using anhydrous calcium chloride, filtered off, and evaporated. The product was isolated by column chromatography on silica gel in a chloroform–methanol solvent system (a methanol gradient from 0 to 20%).

*2-(hexylamino)-1-(4-methoxyphenyl)ethanol (* **4S-C2** *)*

Reaction mixture composition: 0.5 g (2.70 mmol) of 4-methoxybromoacetophenone **1A**, 0.1 g (2.70 mmol) of NaBH_4_, 1.75 mL (13.5 mmol) of hexylamine, and 0.19 g (3.30 mmol) of KOH. Product yield was 0.37 g (54%). R_f_ = 0.45 (10% methanol in chloroform). The content of the target substance was 98% (based on HPLC). ^1^H NMR spectrum (CDCl_3_) δ: 7.30–7.27 (m, 2H, Ph); 6.87–6.84 (m, 2H, Ph); 4.86–4.82 (m, 1H, -CH); 3.82 (br.s, 1H, -OH); 3.78 (s, 3H, O-CH_3_); 2.94–2.67 (m, 4H, 2×CH_2_); 1.59–1.52 (m, 2H, -CH_2_-); 1.29–1.23 (m, 6H, 3×CH_2_); 0.88–0.84 (m, 3H, -CH_3_). ^13^C NMR spectrum (CDCl_3_) δ: 159.13; 133.70; 127.06; 113.80; 70.24; 56.30; 55.24; 49.11; 31.47; 28.39; 26.67; 22.51; 13.99. For C_15_H_25_NO_2_, the calculated and experimental [M+H]^+^ values were 252.1963 and 252.1966, respectively.

*2-((2-hydroxyethyl)amino)-1-(4-methoxyphenyl)ethanol (* **5S-C3** *)*

Reaction mixture composition: 0.5 g (2.70 mmol) of 4-methoxybromoacetophenone **1A**, 0.1 g (2.70 mmol) of NaBH_4_, 0.82 mL (13.5 mmol) of ethanolamine, and 0.19 g (3.30 mmol) of KOH. Product yield was 0.33 g (58%). Product R_f_ = 0.40 (20% methanol in chloroform). The content of the target substance was 99% (based on HPLC). ^1^H NMR spectrum (DMSO-d_6_) δ: 7.44–7.41 (m, 2H, Ph); 6.96–6.93 (m, 2H, Ph); 5.40 (br.s., 1H, -CH_2_-OH-); 5.02 (br.s., 1H, -CH-OH-); 4.11–4.07 (m, 1H, -CH-); 3.74 (s, 3H, -O-CH_3_); 3.71–3.32 (m, 4H, -CH_2_-CH_2_-OH); 2.75–2.56 (m, 2H, -CH_2_-NH-). ^13^C NMR spectrum (DMSO-d_6_) δ: 159.30; 129.63; 127.28; 113.96; 62.92; 57.21; 55.11; 47.79. For C_11_H_17_NO_3_, the calculated and experimental [M+H]^+^ values were 212.1286 and 212.1290, respectively.

*2-((2-hydroxy-2-(4-methoxyphenyl)ethyl)amino)propane-1,3-diol (* **18S-C5** *)*

Reaction mixture composition: 0.5 g (2.20 mmol) of 4-methoxybromoacetophenone **1A**, 0.08 g (2.20 mmol) of NaBH_4_, 1.0 g (11.0 mmol) of 2-aminopropane-1,3-diol, and 0.15 g (2.60 mmol) of KOH. Product yield of **18S-C5** was 0.07 g (13%). Product R_f_ = 0.55 (15% methanol in chloroform). The content of the target substance was 96%. ^1^H NMR spectrum (DMSO-d_6_) δ: 7.26–7.21 (m, 2H, Ph); 6.87–6.82 (m, 2H, Ph); 4.72 (br.s., 1H, -CH-OH); 4.31 (br.s., 1H, -CH_2_-OH); 4.21 (br.s., 1H, -CH-OH); 3.78–2.74 (m, 1H, -CH-NH-); 3.71 (s, 3H, -O-CH_3_); 3.42–3.23 (m, 5H, -CH_2_-NH- and -CH-(CH_2_-OH)_2_); 2.36–2.31 (m, 1H, -CH_2_-NH-). ^13^C NMR spectrum (DMSO-d_6_) δ: 158.17; 134.47; 128.42; 113.43; 66.90; 61.89; 61.35; 57.69; 54.93. For C_12_H_20_NO_4_, the calculated and experimental [M+H]^+^ values were 242.1392 and 242.1395, respectively.

*3-((2-hydroxy-2-(4-methoxyphenyl)ethyl)amino)propane-1,2-diol (* **19S-C6** *)*

Reaction mixture composition: 0.5 g (2.20 mmol) of 4-methoxybromoacetophenone **1A**, 0.08 g (2.20 mmol) of NaBH_4_, 0.85 mL (11.0 mmol) of 3-aminopropane-1,2-diol, and 0.15 g (2.60 mmol) of KOH. Product yield was 0.05 g (9%). Product R_f_ = 0.46 (13% methanol in chloroform). The content of the target substance was 98%. ^1^H NMR spectrum (CD_3_OD) δ: 7.27–7.22 (m, 2H, Ph); 6.90–6.86 (m, 2H, Ph); 3.76 (s, 3H, -O-CH_3_); 3.61–3.43 (m, 4H, -CH_2_-CH(OH)-CH_2_-OH); 3.30–3.28 (m, 1H, -CH(OH)-CH_2_-OH); 2.65–2.40 (m, 2H, -CH_2_-NH-). ^13^C NMR spectrum (CD_3_OD) δ: 160.78; 132.86; 132.76; 129.92; 129.89; 115.06; 115.04; 67.41; 67.27; 66.26; 66.13; 66.03; 65.07; 55.73; 51.53; 50.75. For C_12_H_20_NO_4_, the calculated and experimental [M+H]^+^ values were 242.1392 and 242.1394, respectively.

*1-(4-(benzyloxy)phenyl)-2(hexylamino)ethanol (* **10S-E2** *)*

Reaction mixture composition: 0.5 g (1.92 mmol) of 4-benzyloxybromoacetophenone **1B**, 0.07 g (1.92 mmol) of NaBH_4_, 1.25 mL (9.6 mmol) of hexylamine, and 0.17 g (3.0 mmol) KOH. Product yield was 0.17 g (27%). R_f_ = 0.55 (20% methanol in chloroform). The content of the target substance was 98%. ^1^H NMR spectrum (CDCl_3_) δ: 7.43–7.33 (m, 7H, Ph); 6.94–6.92 (m, 2H, Ph); 5.38 (br. s, 1H, -CH-OH-); 5.03 (s, 2H, -CH_2_-O-); 4.23 (br.s., 1H, -OH); 3.16–3.03 (m, 4H, -CH_2_-NH-CH_2_); 1.90 (br.s., 2H, -CH_2_-(CH_2_)_3_-CH_3_); 1.35–1.28 (m, 6H, 3×CH_2_); 0.89–0.85 (m, 3H, -CH_3_). ^13^C NMR spectrum (CDCl_3_) δ: 158.66; 136.76; 132.37; 128.56; 127.96; 127.41; 127.18; 69.97; 68.69; 55.11; 48.65; 31.13; 26.36; 25.82; 22.38; 13.92. For C_21_H_29_NO_2_, the calculated and experimental [M+H]^+^ values were 328.2277 and 328.2281, respectively.

*1-(4-(benzyloxy)phenyl)-2-((2-hydroxyethyl)amino)ethanol (* **8S-E3** *)*

Reaction mixture composition: 0.65 g (2.50 mmol) of 4-benzyloxybromoacetophenone **1B**, 0.09 g (2.50 mmol) NaBH_4_, 0.75 mL (12.5 mmol) ethanolamine, and 0.17 g (3.0 mmol) KOH. Product yield was 0.16 g (22%). R_f_ = 0.35 (20% methanol in chloroform). The content of the target substance was 98%. ^1^H NMR spectrum (DMSO-d_6_) δ: 7.45–7.29 (m, 7H, Ph); 7.03–7.00 (m, 2H, Ph); 5.27 (br.s, 1H, -CH-OH); 5.09 (s, 2H, -O-CH_2_-); 4.89 (br.s, 1H, -CH_2_-OH); 4.04–4.00 (m, 1H, -CH-); 3.64–3.55 (m, 4H, -CH_2_-CH_2_-OH); 2.75–2.54 (m, 2H, -CH_2_-CH(OH)). ^13^C NMR spectrum (DMSO-d_6_) δ: 158.28; 135.95; 129.42; 128.37; 127.78; 127.59; 114.73; 88.84; 69.18; 69.46; 63.00; 57.65; 48.01. For C_17_H_21_NO_3_, the calculated and experimental [M+H]^+^ values were 288.1599 and 288.1602, respectively.

*2-((2-(4-(benzyloxy)phenyl)-2-hydroxyethyl)amino)propane-1,3-diol (* **20S-E5** *)*

Reaction mixture composition: 1.0 g (3.30 mmol) of 4-benzyloxybromoacetophenone **1B**, 0.12 g (3.30 mmol) NaBH_4_, 1.5 g (16.5 mmol) 2-aminopropane-1,3-diol, and 0.22 g (4.0 mmol) KOH. Product yield was 0.27 g (27%). R_f_ = 0.67 (13% methanol in chloroform). The content of the target substance was 98%. ^1^H NMR spectrum (DMSO-d_6_) δ: 7.45–7.35 (m, 5H, Ph); 7.25–7.22 (m, 2H, Ph); 6.96–6.91 (m, 2H, Ph); 5.18 (br.s., 1H, -CH-OH); 5.07 (s., 2H, -O-CH_2_-); 4.53–4.49 (m., 1H, -CH-OH); 4.44–4.26 (m, 2H, 2×-CH_2_-OH-); 3.44–3.24 (m, 5H, -CH-(-CH_2_-OH)_2_); 2.67–2.51 (m, 2H, -CH_2_-NH-). ^13^C NMR spectrum (DMSO-d_6_) δ: 157.25; 137.24; 136.87; 128.43; 127.77; 127.63; 127.07; 114.25; 71.61; 69.12; 61.36; 61.24; 61.08; 55.64. For C_18_H_24_NO_4_, the calculated and experimental [M+H]^+^ values were 318.1705 and 318.1707, respectively.

*3-((2-(4-(benzyloxy)phenyl)-2-hydroxyethyl)amino)propane-1,2-diol (* **21S-E6** *)*

Reaction mixture composition: 1.0 g (3.30 mmol) of 4-benzyloxybromoacetophenone **1B**, 0.12 g (3.30 mmol) of NaBH_4_, 1.27 mL (16.5 mmol) of 3-aminopropane-1,2-diol, and 0.22 g (4.0 mmol) of KOH. Product yield was 0.12 g (12%). R_f_ = 0.75 (15% methanol in chloroform). The content of the target substance was 98%. ^1^H NMR spectrum (DMSO-d_6_) δ: 7.45–7.29 (m, 5H, Ph); 7.25–7.20 (m, 2H, Ph); 6.95–6.92 (m, 2H, Ph); 5.05 (s, 2H, -O-CH_2_-); 4.58–4.56 (m, 1H, -CH-OH); 4.34 (br.s, 4H, -CH(OH)-CH_2_-OH and HO-CH-CH_2_-NH-); 3.58–3.18 (m, 5H, -CH_2_-CH(OH)-CH_2_-OH). ^13^C NMR spectrum (DMSO-d_6_) δ: 157.38; 157.33; 137.24; 137.21; 134.11; 134.06; 128.40; 128.36; 127.71; 127.58; 127.54; 127.01; 126.98; 114.39; 71.01; 70.15; 69.14; 66.75; 66.60; 64.76; 64.48; 63.89; 51.15; 50.35. For C_18_H_24_NO_4_, the calculated and experimental [M+H]^+^ values were 318.1705 and 318.1708, respectively.

*2-(hexylamino)-1-(4-nitrophenyl)ethanol (* **13S-G2** *)*

Reaction mixture composition: 0.85 g (3.50 mmol) of 4-nitrobromoacetophenone **1C**, 0.13 g (3.50 mmol) of NaBH_4_, 2.30 mL (17.5 mmol) of hexylamine, and 0.23 g (4.2 mmol) of KOH. Obtained 0.32 g (34%) of the product **13S-G2**. R_f_ = 0.61 (15% methanol in chloroform). The content of the target substance was 98%. ^1^H NMR spectrum (DMSO-d_6_) δ: 8.18–8.16 (m, 2H, Ph); 7.62–7.59 (m, 2H, Ph); 5.58 (br.s, 1H,-OH); 4.77–4.73 (m, 1H, -CH); 2.69–2.53 (m, 4H, -CH_2_-NH-CH_2_-); 1.40–1.35 (m, 2H, -NH-CH_2_-CH_2_-); 1.26–1.22 (m, 6H, -(CH_2_)_3_-CH_3_); 0.86–0.81 (m, 3H, -CH_3_). ^13^C NMR spectrum (DMSO-d_6_) δ: 152.80; 146.37; 127.11; 123.08; 70.78; 57.20; 49.01; 31.25; 29.53; 26.48; 22.12; 13.94. For C_14_H_23_N_2_O_3_, the calculated and experimental [M+H]^+^ values were 267.1709 and 267.1712, respectively.

*2-((2-hydroxyethyl)amino)-1-(4-nitrophenyl)ethanol (* **14S-G3** *)*

Reaction mixture composition: 1.25 g (5.14 mmol) of 4-nitrobromoacetophenone **1C**, 0.19 g (5.14 mmol) of NaBH_4_, 1.55 mL (25.7 mmol) of ethanolamine, and 0.34 g (6.17 mmol) of KOH. Obtained 0.41 g (35%) of the product **14S-G3**. R_f_ = 0.55 (13% methanol in chloroform). The content of the target substance was 95%. ^1^H NMR spectrum (DMSO-d_6_) δ: 8.19–8.16 (m, 2H, Ph); 7.63–7.60 (m, 2H, Ph); 5.61 (br.s, 1H, -CH-OH); 4.78–4.74 (m, 1H, -CH-OH); 4.49 (br.s, 1H, -CH_2_-OH); 3.45–3.37 (m, 2H, -CH_2_- OH); 2.72–2.53 (m, 4H, -CH_2_-NH-CH_2_-). ^13^C NMR spectrum (DMSO-d_6_) δ: 152.76; 146.39; 127.12; 123.11; 70.96; 60.42; 57.15; 51.45. For C_10_H_15_N_2_O_4_, the calculated and experimental [M+H]^+^ values were 227.1032 and 227.1034, respectively.

*2-((2-hydroxy-2-(4-nitrophenyl)ethyl)amino)propane-1,3-diol (* **22S-G5** *)*

Reaction mixture composition: 1.7 g (7.00 mmol) of 4-nitrobromoacetophenone **1C**, 0.265 g (7.00 mmol) of NaBH_4_, 3.18 g (35.0 mmol) of 2-aminopropane-1,3-diol, and 0.47 g (8.4 mmol) of KOH. Obtained 0.72 g (40%) of the product **22S-G5**. R_f_ = 0.58 (10% methanol in chloroform). The content of the target substance was 98%. ^1^H NMR spectrum (DMSO-d_6_) δ: 8.18–8.15 (m, 2H, Ph); 7.64–7.61 (m, 2H, Ph); 5.51 (br.s., 1H, -CH-OH); 4.74–4.72 (m, 1H, -CH-OH); 4.31–4.27 (m, 2H, 2×-CH_2_-OH); 3.43–3.24 (m, 4H, 2×-CH_2_-OH-); 2.84–2.64 (m, 2H, -CH_2_-NH); 2.55–2.51 (m, 1H, -CH-NH-). ^13^C NMR spectrum (DMSO-d_6_) δ: 152.69; 146.39; 127.13; 123.13; 71.39; 61.39; 61.23; 60.98; 55.10. For C_11_H_17_N_2_O_5_, the calculated and experimental [M+H]^+^ values were 257.1137 and 257.1140, respectively.

*3-((2-hydroxy-2-(4-nitrophenyl)ethyl)amino)propane-1,2-diol (* **23S-G6** *)*

Reaction mixture composition: 1.7 g (7.00 mmol) of 4-nitrobromoacetophenone **1C**, 0.265 g (7.00 mmol) of NaBH_4_, 2.7 mL (35.0 mmol) of 3-aminopropane-1,2-diol, and 0.47 g (8.4 mmol) of KOH. Obtained 0.52 g (29%) of the product **23S-G6**. R_f_ = 0.49 (10% methanol in chloroform). The content of the target substance was 98%. ^1^H NMR spectrum (DMSO-d_6_) δ: 8.19–8.16 (m, 2H, Ph); 7.63–7.60 (m, 2H, Ph); 5.62 (br.s, 1H, -CH-OH); 4.77–4.57 (m, 1H, -CH-OH); 4.57 (br.s, 1H, -CH_2_-OH); 3.49 (br.s, 1H, -CH-OH); 3.34–3.23 (m, 3H, -CH(OH)-CH_2_-OH); 2.72–2.52 (m, 3H, -CH_2_-NH-CH_2_-); 2.46–2.38 (m, 1H, -CH_2_-NH). ^13^C NMR spectrum (DMSO-d_6_) δ: 152.73; 146.41; 127.11; 123.13; 71.08; 70.68; 64.54; 57.48; 52.73. For C_11_H_17_N_2_O_5_, the calculated and experimental [M+H]^+^ values were 257.1137 and 257.1141, respectively.

*1-(4-nitrophenyl)-2-piperidin-1-ylethanol (* **9S-G1** *)*

Reaction mixture composition: 0.5 g (2.05 mmol) of 4-nitrobromoacetophenone **1C**, 0.08 g (2.05 mmol) of NaBH_4_, 1.0 mL (10.2 mmol) of piperedine, and 0.14 g (2.46 mmol) of KOH. Product yield was 0.20 g (40%). R_f_ = 0.67 (5% methanol in chloroform). The content of the target substance was 97%. ^1^H NMR spectrum (CDCl_3_) δ: 8.19–8.16 (m, 2H, Ph); 7.57–7.55 (m, 2H, Ph); 5.05–5.00 (m, 1H, -CH-); 4.40 (br.s., 1H, -OH); 2.91–2.84 (m, 2H, -CH_2_-CH-); 2.70–2.49 (m, 4H, piperidine 2×CH_2_); 1.78–1.70 (m, 4H, piperidine 4×CH_2_); 1.54–1.50 (m, 2H, piperidine CH_2_). ^13^C NMR spectrum (CDCl_3_) δ: 149.41; 147.30; 126.53; 123.65; 67.71; 65.99; 54.63; 25.11; 23.45. For C_13_H_18_N_2_O_3_, the calculated and experimental [M+H]^+^ values were 251.1396 and 251.1398, respectively.

#### 2.1.5. General Procedure for the Synthesis of the Products **12S-B2**, **2S-B3**, **27S-B5**, **17S-B6**, **26S-F2**, **16S-F3**, **24S-F5**, **25S-F6**, and **15S-F1**

Initially, 10% Pd on carbon (0.05 eq of Pd) was added to a solution of **12S-B2**, **2S-B3**, **27S-B5**, **17S-B6**, **26S-F2**, **16S-F3**, **24S-F5**, **25S-F6**, or **15S-F1** in ethanol and mixed in an atmosphere of H_2_ at room temperature overnight. The reaction mass was filtered through a layer of celite washed with 10–15 mL ethanol, and the combined organic solutions were evaporated. The product was isolated by column chromatography on silica gel in a chloroform–methanol solvent system (methanol gradient from 0 to 35%).

*4-(2-(hexylamino)-1-hydroxyethyl)phenol (* **12S-B2** *)*

Obtained 0.03 g (41%) of the product **12S-B2** from 0.10 g (0.3 mmol) of **10S-E2**. R_f_ = 0.34 (20% methanol in chloroform). The content of the target substance was 97%. ^1^H NMR spectrum (DMSO-d_6_) δ: 7.16–7.12 (m, 2H, Ph); 6.76–6.71 (m, 2H, Ph); 4.73–4.68 (m, 1H, -CH); 3.86–2.71 (m, 4H, -CH_2_-NH-CH_2_-); 1.58–1.48 (m, 2H, -NH-CH_2_-CH_2_-); 1.30–1.25 (m, 6H, -(CH_2_)_3_-CH_3_); 1.29–0.87–0.84 (m, 3H, -CH_3_). ^13^C NMR spectrum (DMSO-d_6_) δ: 156.75; 133.09; 127.06; 114.91; 69.18; 55.24; 47.78; 30.93; 26.88; 26.02; 21.98; 13.90. For C_14_H_24_NO_2_, the calculated and experimental [M+H]^+^ values were 238.1807 and 238.1810, respectively.

*4-(1-hydroxy-2-((2-hydroxyethyl)amino)ethyl)phenol (* **2S-B3** *)*

Obtained 0.05 g (73%) of the product **2S-B3** from 0.1 g (0.35 mmol) of **8S-E3**. R_f_ = 0.35 (30% methanol in chloroform). The content of the target substance was 97%. ^1^H NMR spectrum (CD_3_OD) δ: 7.36–7.33 (m, 2H, Ph); 6.87–6.85 (m, 2H, Ph); 5.01 (br.s, 2H, 2 × OH); 4.33–4.28 (m, 1H, -CH-OH); 3.99–3.86 (m, 2H, -CH_2_-OH); 3.83–3.69 (m, 2H, -CH_2_-NH-); 3.05–2.91 (m, 2H, -CH_2_-CH-); 2.15 (s, 1H, -OH). ^13^C NMR spectrum (CD_3_OD) δ: 159.90; 130.97; 124.11; 117.02; 64.74; 63.39; 57.78; 30.76. For C_10_H_15_NO_3_, the calculated and experimental [M+H]^+^ values were 198.1130 and 198.1134, respectively.

*2-((2-hydroxy-2-(4-hydroxyphenyl)ethyl)amino)propane-1,3-diol (* **27S-B5** *)*

Obtained 0.03 g (25%) of the product **27S-B5** from 0.17 g (0.5 mmol) of **20S-E5**. R_f_ = 0.23 (15% methanol in chloroform). The content of the target substance was 96%. ^1^H NMR spectrum (DMSO-d_6_) δ: 7.13–7.09 (m, 2H, Ph); 6.70–6.65 (m, 2H, Ph); 4.47–4.43 (m, 1H, -CH-OH); 3.44–3.20 (m, 5H, -CH-(-CH_2_-OH)_2_); 2.69–2.52 (m, 2H, -CH_2_-NH). ^13^C NMR spectrum (DMSO-d_6_) δ: 156.14; 134.72; 126.96; 114.62; 71.67; 61.29; 61.17; 60.98; 55.51. For C_11_H_18_NO_4_, the calculated and experimental [M+H]^+^ values were 228.1235 and 228.1239, respectively.

*3-((2-hydroxy-2-(4-hydroxyphenyl)ethyl)amino)propane-1,2-diol (* **17S-B6** *)*

Obtained 0.03 g (42%) of the product **17S-B6** from 0.1 g (0.3 mmol) of **21S-E6**. R_f_ = 0.37 (20% methanol in chloroform). The content of the target substance was 98%. ^1^H NMR spectrum (DMSO-d_6_) δ: 7.12–7.08 (m, 2H, Ph); 6.70–6.66 (m, 2H, Ph); 4.82 (br.s, 1H, -CH-OH); 4.82 (br.s, 2H, -CH(OH)-CH_2_-OH); 4.06 (br.s, 1H, -CH-OH); 3.60–3.17 (m, 7H, -CH_2_-CH(OH)-CH_2_-OH and -CH-OH and Ph-OH); 2.47–2.19 (m, 2H, -CH_2_-NH). ^13^C NMR spectrum (DMSO-d_6_) δ: 156.38; 156.34; 131.28; 128.44; 128,33; 114,88; 70.70; 69.73; 64,69; 64,40; 63,84; 50,92; 50.10. For C_11_H_18_NO_4_, the calculated and experimental [M+H]^+^ values were 228.1235 and 228.1238, respectively.

*1-(4-aminophenyl)-2-(hexylamino)ethanol (* **26S-F2** *)*

Obtained 0.035 g (37%) of the product **26S-F2** from 0.1 g (0.4 mmol) of **13S-G2**. R_f_ = 0.41 (10% methanol in chloroform). The content of the target substance was 95%. ^1^H NMR spectrum (DMSO-d_6_) δ: 6.96–6.94 (m, 2H, Ph); 6.51–6.47 (m, 2H, Ph); 4.86 (br.s, 2H,-NH_2_); 4.42–4.37 (m, 1H, -CH); 2.59–2.46 (m, 4H, -CH_2_-NH-CH_2_-); 1.40–1.32 (m, 2H, -NH-CH_2_-CH_2_-); 1.27–1.22 (m, 6H, -(CH_2_)_3_-CH_3_); 0.87–0.83 (m, 3H, -CH_3_). ^13^C NMR spectrum (DMSO-d_6_) δ: 147.39; 131.63; 126.53; 113.40; 71.27; 57.73; 49.02; 31.18; 29.58; 26.42; 22.02; 13.84. For C_14_H_25_N_2_O, the calculated and experimental [M+H]^+^ values were 237.1967 and 237.1970, respectively.

*1-(4-aminophenyl)-2-((2-hydroxyethyl)amino)ethanol (* **16S-F3** *)*

Obtained 0.062 g (79%) of the product **16S-F3** from 0.09 g (0.4 mmol) of **14S-G3**. R_f_ = 0.31 (13% methanol in chloroform). The content of the target substance was 98%. ^1^H NMR spectrum (DMSO-d_6_) δ: 6.97–6.94 (m, 2H, Ph); 6.49–6.47 (m, 2H, Ph); 4.90 (br.s, 2H, 2×-OH); 4.42–4.38 (m, 1H, -CH-OH); 3.43–3.40 (m, 2H, -CH_2_-OH); 2.61–2.54 (m, 4H, -CH_2_-NH-CH_2_-). ^13^C NMR spectrum (DMSO-d_6_) δ: 147.52; 131.66; 126.64; 113.46; 71.51; 60.43; 57.75; 51.53. For C_10_H_17_N_2_O_2_, the calculated and experimental [M+H]^+^ values were 197.1290 and 197.1294, respectively.

*2-((2-(4-aminophenyl)-2-hydroxyethyl)amino)propane-1,3-diol (* **24S-F5** *)*

Obtained 0.1 g (88%) of the product **24S-F5** from 0.14 g (0.5 mmol) of **22S-G5**. R_f_ = 0.26 (10% methanol in chloroform). The content of the target substance is 98%. ^1^H NMR spectrum (DMSO-d_6_) δ: 6.97–6.94 (m, 2H, Ph); 6.49–6.46 (m, 2H, Ph); 4.92–4.90 (m, 3H, -NH_2_ and -CH-OH); 4.41–4.35 (m, 3H, -CH-OH and 2×-CH_2_-OH); 3.30–3.23 (m, 5H, -CH-(CH_2_-OH)_2_); 2.64–2.56 (m, 2H, -CH_2_-NH). ^13^C NMR spectrum (DMSO-d_6_) δ: 147.49; 131.67; 126.65; 113.44; 71.99; 61.37; 61.28; 61.11; 55.68. For C_11_H_19_N_2_O_3_, the calculated and experimental [M+H]^+^ values were 227.1396 and 227.1400, respectively.

*3-((2-(4-aminophenyl)-2-hydroxyethyl)amino)propane-1,2-diol (* **25S-F6** *)*

Obtained 0.095 g (53%) of the product **25S-F6** from 0.2 g (0.8 mmol) of **23S-G6**. R_f_ = 0.23 (10% methanol in chloroform). The content of the target substance was 95%. ^1^H NMR spectrum (DMSO-d_6_) δ: 6.97–6.94 (m, 2H, Ph); 6.50–6.47 (m, 2H, Ph); 4.90 (br.s, 3H, -NH_2_ and -CH-OH); 4.43–4.38 (m, 1H, -CH-OH); 3.53–3.23 (m, 5H, -CH(OH)-CH_2_-OH and -CH_2_-NH); 2.63–2.52 (m, 3H, -CH_2_-NH-CH_2_-); 2.44–2.37 (m, 1H, -CH_2_-NH). ^13^C NMR spectrum (DMSO-d_6_) δ: 147.53; 131.66; 126.63; 113.48; 71.59; 70.65; 64.63; 58.09; 52.83. For C_11_H_19_N_2_O_3_, the calculated and experimental [M+H]^+^ values were 227.1396 and 227.1399, respectively.

*1-(4-aminophenyl)-2-piperidin-1-ylethanol (* **15S-F1** *)*

Obtained 0.03 g (34%) of the product **15S-F1** from 0.1 g (0.4 mmol) of **9S-G1**. R_f_ = 0.35 (10% methanol in chloroform). The content of the target substance was 95%. ^1^H NMR spectrum (DMSO-d_6_) δ: 7.04–7.01 (m, 2H, Ph); 6.54–6.51 (m, 2H, Ph); 5.86 (br.s., 1H, -OH); 5.09 (br.s., 2H, -NH_2_); 4.95–4.91 (m, 1H, -CH-); 3.36–2.98 (m, 6H, piperidine 2×CH_2_ and -CH-CH_2_-); 1.77–1.76 (m, 4H, piperidine 4×CH_2_); 1.51 (br.s., 2H, piperidine CH_2_). ^13^C NMR spectrum (DMSO-d_6_) δ: 148.24; 128.69; 126.70; 113.47; 66.72; 62.98; 52.49; 22.24; 21.46. For C_13_H_21_N_2_O, the calculated and experimental [M+H]^+^ values were 221.1654 and 221.1656, respectively.

### 2.2. Biology

#### 2.2.1. Cell Cultures

Chronic myeloid leukemia cell line K562 and mantle cell lymphoma cell line Granta were obtained from ATCC and cultured as described [9].

#### 2.2.2. MTT Cytotoxicity Assay

Cell cultures were seeded into 96-well plates and incubated overnight under 5% CO_2_ at 37 °C for 24 h. Then, cells were treated with a synthesized compound (in the concentration range of 10 mM–100 nM) or a vehicle for 24 h and 72 h. Then, 20 μL of MTT solution was added to each well and mixed. After 4 h, the supernatants were removed, and 100 μL of DMSO was added to each well to dissolve the precipitate. The cells’ viability was estimated by measuring absorbance at 570 nm using the MultiScan MCC 340 spectrophotometer (Thermo Fisher, Waltham, MA, USA). IC_50_ values were determined with GraphPad Prism (Ver.7.0). Results are presented as mean ± standard deviation (SD).

#### 2.2.3. Molecular Docking

Molegro Virtual Docker 6.0 software was used to perform molecular docking. The structure of GR (PDB ID: 1P93) was chosen as a target. The target structure was prepared automatically using standard procedures of the Molegro Virtual Docker package. Ligand structures were constructed and optimized by molecular dynamic methods in the MMFF94 force field using Avogadro 1.2.0. The MolDock Score was used as a scoring function; dexamethasone (CID 5743) served as a reference ligand. Molecular docking was carried out in 40 iterations. MolDock SE was used as a docking algorithm following energy minimization and optimization of hydrogen bonds.

#### 2.2.4. Statistical Analysis

Mean and standard deviation values were calculated using Microsoft Excel and GraphPad Prism (Ver.7.0) software. Treatment effects in each experiment were compared by one-way ANOVA or *t*-test. The differences between groups were considered significant at *p* < 0.05. All experiments were repeated three times.

## 3. Results

### 3.1. Synthesis of 26 Novel Synephrine Derivatives

**Scheme 1:**
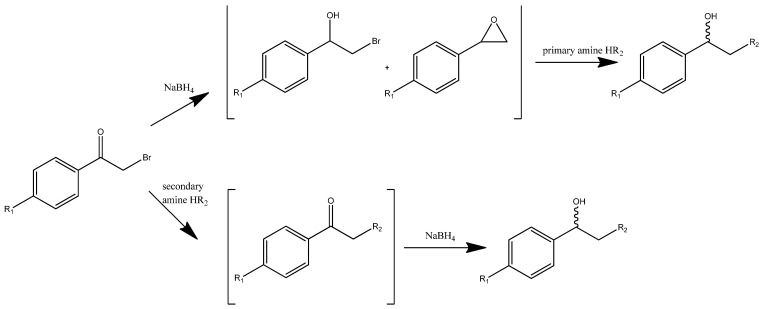
Two synthetic routes to non-steroid Dex analogues.

We aimed to evaluate the potential of 4-(1-hydroxy-2-aminoethyl)benzene derivatives substituted at the amino group and the 4-position of the benzene ring 1 as non-steroidal dexamethasone (Dex) analogs. We employed two methods to synthesize synephrine derivatives with different primary and secondary amines (Scheme 1).

**Scheme 2:**
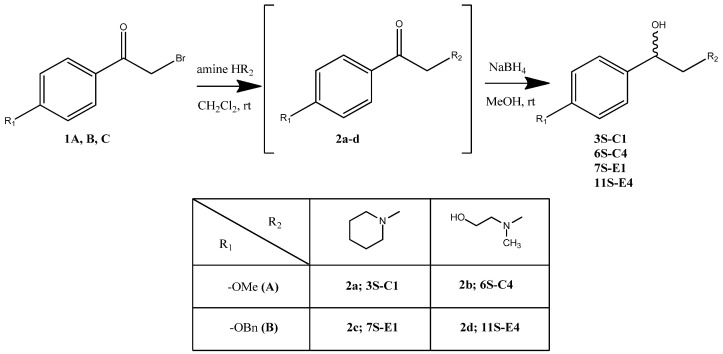
Synthesis of the compounds **3S-C1**, **6S-C4**, **7S-E1**, and **11S-E4**.

The first synthetic route involved the direct alkylation of secondary amines with bromoacetophenones **1** followed by the reduction of intermediates **2a**–**d** with NaBH_4_ (Scheme 2). Bromoacetophenones **1** were obtained by α-bromination of the corresponding commercially available acetophenones.

The direct alkylation of primary amines with bromoacetophenones **1** was less preferable because of the lower selectivity of the reaction; therefore, they were synthesized by another synthetic pathway.

**Scheme 3:**
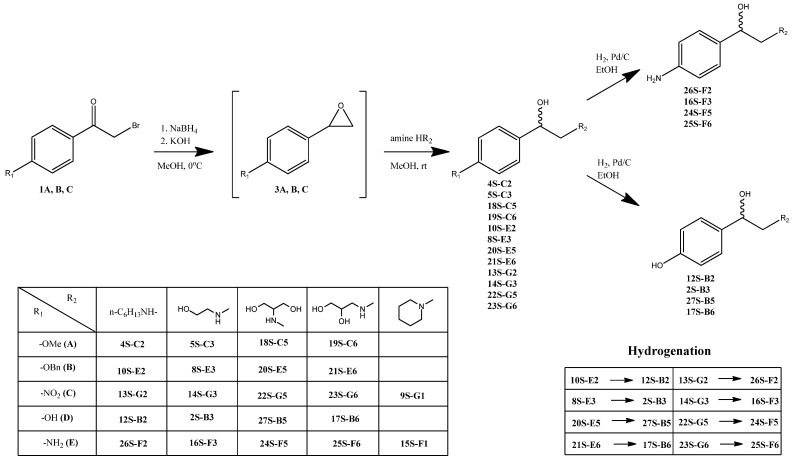
Synthesis of the compounds **4S-C2**, **5S-C3**, **18S-C5**, **19S-C6**, **10S-E2**, **8S-E3**, **20S-E5**, **21S-E6**, **13S-G2**, **14S-G3**, **22S-G5**, **23S-G6** and **12S-B2**, **2S-B3**, **27S-B5**, **17S-B6**, **26S-F2**, **16S-F3**, **24S-F5**, **25S-F6**, and **15S-F1**.

**Scheme 4:**
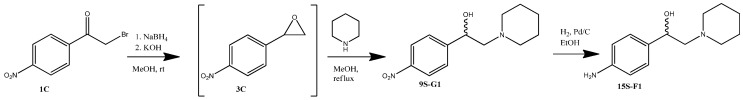
Synthesis of the compounds **9S-G1** and **15S-F1**.

The second strategy was to obtain epoxides **3A**,**B**,**C** in situ by the sequential reduction of bromoacetophenones **1** with NaBH_4_ in methanol and the further epoxidation of the intermediates by adding 1.2 alkali equivalents. The target compounds **4S-C2**, **5S-C3**, **18S-C5**, **19S-C6**, **10S-E2**, **8S-E3**, **20S-E5**, **21S-E6**, **13S-G2**, **14S-G3**, **22S-G5**, and **23S-G6** were obtained by adding a five-fold excess of primary amines to epoxides **3A**,**B**,**C** in line with Scheme 3. The **9S-G1** analog was obtained by a similar strategy to the compounds **4S-C2**, **5S-C3**, **18S-C5**, **19S-C6**, **10S-E2**, **8S-E3**, **20S-E5**, **21S-E6**, **13S-G2**, **14S-G3**, **22S-G5**, and **23S-G6**, according to Scheme 4, except that the reaction was carried out at reflux. The first synthetic pathway for the synthesis of **3S-C1**, **6S-C4**, **7S-E1**, and **11S-E4** (Scheme 2) was not optimal for **9S-G1** synthesis due to the low reactivity of the source bromide **1C**. The compounds **12S-B2**, **2S-B3**, **27S-B5**, and **17S-B6**, as well as compounds **26S-F2**, **16S-F3**, **24S-F5**, **25S-F6**, and **15S-F**, were obtained by the hydrogenation of **10S-E2**, **8S-E3**, **20S-E5**, **21S-E6**, **13S-G2**, **14S-G3**, **22S-G5**, **23S-G6**, and **9S-G1**, respectively (Scheme 3 and Scheme 4) using palladium on carbon.

The compounds used for the initial scrutiny of their biological properties were synthesized as a mixture of enantiomers.

In summary, we obtained 26 synephrine analogs. Their structures were confirmed by ^1^H and ^13^C NMR spectroscopy and high-resolution mass spectrometry. The purity of the obtained compounds was assessed using HPLC with a mass spectrometer. Compounds with purity greater than 95% were further subjected to biological activity evaluation.

### 3.2. The Evaluation of Cytotoxicity of Novel Synephrine Derivatives

**Figure 2:**
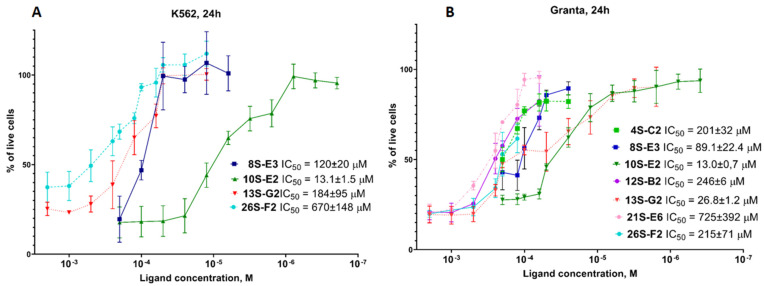
The cytotoxic effects of synephrine derivatives on K562 (**A**) and Granta (**B**) cells after 24 h incubation. Chronic myeloid leukemia cell line K562 (**A**) and mantle cell lymphoma cell line Granta (**B**) were seeded into 96-well plates and incubated overnight under 5% CO_2_ at 37 °C for 24 h. Next, cells were treated with **3S-C1**, **6S-C4**, **7S-E1**, **11S-E4**, **4S-C2**, **5S-C3**, **18S-C5**, **19S-C6**, **10S-E2**, **8S-E3**, **20S-E5**, **21S-E6**, **13S-G2**, **14S-G3**, **22S-G5**, **23S-G6**, **12S-B2**, **2S-B3**, **27S-B5**, **17S-B6**, **26S-F2**, **16S-F3**, **24S-F5**, **25S-F6**, **15S-F**, **9S-G1** (100 nM–10 mM), or vehicle for 24 h. Then, 20 μL of MTT solution was added to each well and mixed. After 4 h, the supernatants were removed and 100 μL DMSO was added to each well to dissolve the precipitate. Cell viability was estimated by measuring absorbance at 570 nm using the MultiScan MCC 340 spectrophotometer (Thermo Fisher, Waltham, MA, USA). IC_50_ values were determined using GraphPad Prism, Ver.7.0. Results are presented as mean ± standard deviation (SD).

The cytotoxic effects of new synthetic synephrine derivatives were evaluated using the MTT assay in chronic myelocytic leukemia K562 cells and mantle cell lymphoma Granta cells, well-characterized cell lines used in our previous studies [9,14,17,23]. Cells were treated with synephrine derivatives in a wide concentration range of 10 mM–100 nM for 24 h. Ultimately, **8S-E3**, **10S-E2**, **13S-G2,** and **26S-F2** exhibited the strongest cytotoxic effect in K562 cells, with IC_50_ values of 120 ± 20 µM, 13.1 ± 1.5 µM, 184 ± 95 µM, and 670 ± 148 µM, respectively (Figure 2A). Granta-519 cells were more sensitive to **4S-C2**, **8S-E3**, **10S-E2**, **12S-B2**, **13S-G2**, **21S-E6**, and **26S-F2**, with IC_50_ values of 201 ± 32 µM, 89.1 ± 22.4 µM, 13 ± 0.7µM, 246 ± 6 µM, 26.8 ± 1.2 µM, 725 ± 392 µM, and 215 ± 71 µM, respectively (Figure 2B). The other tested synephrine derivatives did not have IC_50_ values, which most likely exceeded 1 mM. The highest cytotoxicity in both cell lines was demonstrated for the compound 1-[4-(benzyloxy)phenyl]-2-(hexylamino)ethanol (**10S-E2)** at the concentration of 13 µM. Taken together, these results and the data on **10S-E2**’s affinity for the GR make this synephrine derivative an attractive candidate for further investigation. Another potential target is 2-(hexylamino)-1-(4-nitrophenyl)ethanol (**13S-G2**), with the IC_50_ value of 26.8 ± 1.2 µM in Granta cells.

**Figure 3:**
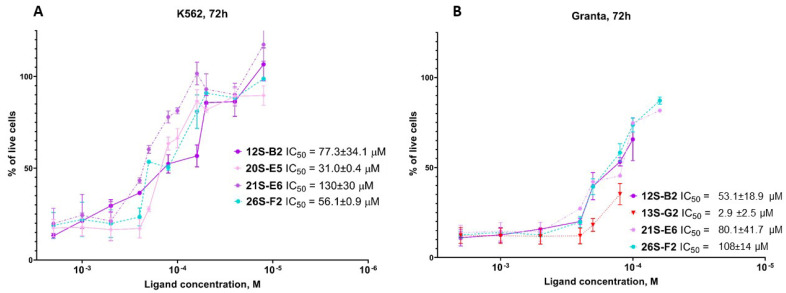
The cytotoxic effects of synephrine derivatives on K562 (**A**) and Granta (**B**) cells after 72 h incubation. Chronic myeloid leukemia cell line K562 (**A**) and mantle cell lymphoma cell line Granta (**B**) were seeded into 96-well plates and incubated overnight under 5% CO_2_ at 37 °C for 72 h. Next, cells were treated with **3S-C1**, **6S-C4**, **7S-E1**, **11S-E4**, **4S-C2**, **5S-C3**, **18S-C5**, **19S-C6**, **20S-E5**, **21S-E6**, **14S-G3**, **22S-G5**, **23S-G6**, **12S-B2**, **2S-B3**, **27S-B5**, **17S-B6**, **16S-F3**, **24S-F5**, **25S-F6**, **15S-F**, **9S-G1** (100 nM–10 mM), or vehicle for 72 h. Then, 20 μL of MTT solution was added to each well and mixed. After 4 h, the supernatants were removed and 100 μL DMSO was added to each well to dissolve the precipitate. Cell viability was estimated by measuring absorbance at 570 nm using the MultiScan MCC 340 spectrophotometer (Thermo Fisher, Waltham, MA, USA). IC_50_ values were determined using GraphPad Prism, Ver.7.0. Results are presented as mean ± standard deviation (SD).

After 72 h incubation with the compounds in the same concentration range (100 nM–10 mM), the IC_50_ values for **12S-B2**, **20S-E5**, **21S-E6**, and **26S-F2** in K562 cells reached 77.3 ± 34.1 µM, 31.0 ± 0.4 µM, 130 ± 30 µM, and 56.1 ± 0.9 µM, respectively. In Granta cells, **20S-E5** did not reach IC_50_, while the IC_50_ values for **12S-B2**, **21S-E6**, and **26S-F2** were 53.1 ± 18.9 µM, 80.1 ± 41.7 µM, and 108 ± 14.1 µM, respectively (Figure 3). During a prolonged treatment, 4-(2-(hexylamino)-1-hydroxyethyl)phenol (**12S-B2**) exhibited cytotoxic effects within 3 days after the treatment at concentrations of 50–70 µM. This may be associated with a gradual induction of the mechanisms underlying cell survival inhibition. Our findings implicate a new dosage regimen for *in vivo* experiments that does not require daily administration. Detailed stability and pharmacokinetic studies will be required to further validate this approach.

All findings on the cytotoxic effects of the synephrine derivatives in K562 and Granta cells after 24 and 72 h incubations are summarized in Table 1.

### 3.3. The Evaluation of GR Affinity In Silico

**Figure 4:**
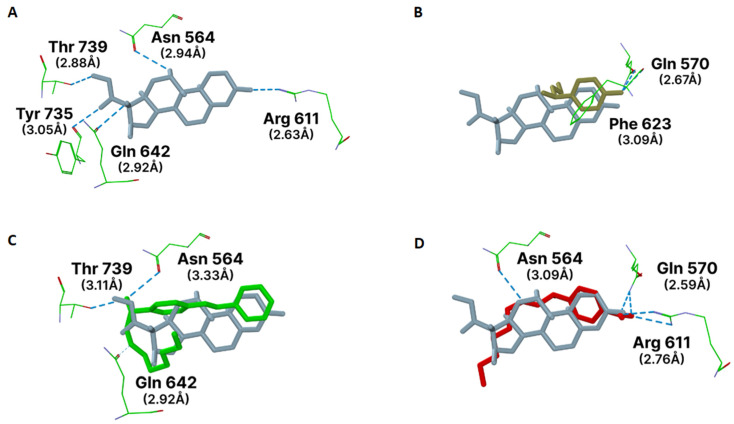
The structure of the GR active site interacting with the analogs of synephrine **10S-C2** (**C**), **13S-G2** (**D**), and synephrine (**B**) compared to Dex (**A**). Molegro Virtual Docker 6.0 software was used for molecular docking. The GR structure (PDB ID: 1P93) was chosen as a target. The target structure was processed automatically by standard procedures of the Molegro Virtual Docker package. Ligand structures were constructed and optimized by molecular dynamic methods in the MMFF94 force field using Avogadro 1.2.0. The MolDock Score was chosen as the scoring function; dexamethasone (CID 5743) served as the reference ligand. Molecular docking was carried out in 40 iterations. MolDock SE was chosen as the docking algorithm following energy minimization and the optimization of hydrogen bonds.

To rank the tested compounds based on their affinity for the GR, we performed molecular docking studies, in order to predict their binding to the ligand binding domain (LBD) of the GR structure (PDB ID: 1P93) using Dex (CID 5743) as a reference ligand. According to the obtained results, the greatest potential affinity for the GR was shown for the derivative **10S-G2**. The MolDock Score, which correlates to the steric orientation and hydrogen bonds in the active site of **10S-E2,** was similar to the score of Dex (−143 and −146, correspondingly; Table 2 and Table S1). The following top five compounds included **21S-E6**, **8S-E3**, **20S-E5**, **7S-E1**, and **13S-G2**. All mentioned compounds occupied a sterically advantageous location at the GR binding site formed by the amino acid residues Thr 739, Asn564, and Gln642 (see Figure 4 and Supplementary Figure S53 for the representative images of molecular docking of novel compounds compared to the Dex background). Dex binding to the LBD mostly involved Thr 739, Asn564, and Gln642 residues (Figure 3 and Supplementary Figure S53).

The affinity of the other synephrine derivatives for the GR *in silico* was more than 1.2-fold lower (Table 2 and Supplementary Table S1). Of note, the template molecule synephrine demonstrated the weakest MolDock Score and potentially the lowest GR affinity.

## 4. Discussion

The biological effects of synephrine, its molecular targets, and a safe pharmacological profile along with the possible mechanisms of action indicate that it can act as a potential SEGRA and can be used as a template to obtain novel SEGRAs [15]. SEGRAs could serve as safer alternatives to GCs for cancer therapy [4,9,10,13,15,17,18,24,25,26]. Therefore, searching for synephrine derivatives opens up new avenues for the development of novel SEGRAs.

Synephrine has itself exhibited anti-cancer and anti-inflammatory properties in several studies [27,28,29,30]. Synephrine is a small molecule with a hydroxyl group located at one terminus lacking a methyl group on the side chain, which affects its stereochemistry and impairs the interaction with proteins [15]. In the present work, we proposed modifications of synephrine with bulky substituents and synthesized 26 synephrine derivatives that could be utilized as potential SEGRAs by two synthetic routes: the direct alkylation of the secondary amines with bromoacetophenones or by interaction between alcohol-derived epoxides that corresponded to bromoacetophenones and primary amines. We estimated the cytotoxic effects of the compounds in K562 and Granta cells as well as their potential affinity for the glucocorticoid receptor (GR) *in silico*. The non-steroidal GR structures were designed in order to preserve the dimensional arrangement of the functional groups in the molecule and remove the dexamethasone (Dex) sterane core, if possible. We varied the lipophilicity of the designed compounds using different substituents at the aromatic ring and the amino group (Supplementary Table S2).

The novel derivative 1-[4-(benzyloxy)phenyl]-2-(hexylamino)ethanol (**10S-E2**) exhibited micromolar-range cytotoxicity in both cell lines after 24 h of treatment, which correlated with the highest affinity for the GR *in silico.* This implies the GR-dependency of **10S-E2**’s cytotoxic effects. The synephrine derivative 2-(hexylamino)-1-(4-nitrophenyl)ethanol (**13S-G2**) with high GR affinity exerted cytotoxic activity at concentrations of 50–70 µM. Furthermore, we revealed a positive correlation between the lipophilicity of the novel synephrine analogs and their biological activity, with the most lipophilic compounds exhibiting higher activity. However, it should be noted that hydroxyl groups in the substituent at the amino group improve the interaction with the GR as the derivatives of aminoethanol (**8S-E3**) and aminopropanediols (**20S-E5**, **21S-E6**) also demonstrate affinity for the GR.

These results imply that synephrine derivatives could serve as promising anti-cancer agents of the SEGRA class. Further investigation is required to elucidate the detailed mechanism of action of the compounds with the highest cytotoxicity *in vitro,* such as their direct interaction with the GR as well as *in vivo* anti-cancer activity. It is especially important to scrutinize the safety of the obtained compounds, including the absence of atrophic or metabolic GC-related side effects.

## 5. Conclusions

In the present work, we synthesized 26 synephrine derivatives that could be used as potential selective glucocorticoid receptor agonists (SEGRAs) via two synthetic routes: the direct alkylation of secondary amines with bromoacetophenones or the interaction between alcohol-derived epoxides corresponding to bromoacetophenones with primary amines. We investigated the *in vitro* anti-cancer effects of the obtained compounds against leukemia and lymphoma cells as well as their potential affinity for the glucocorticoid receptor (GR) *in silico*.

The novel derivative 1-[4-(benzyloxy)phenyl]-2-(hexylamino)ethanol (**10S-E2**) exhibited cytotoxicity in the micromolar range for both cell lines after 24 h of treatment. These effects positively correlated with the highest affinity for the GR *in silico,* implying the GR-dependency of 10S-E2’s activity. The synephrine derivative 2-(hexylamino)-1-(4-nitrophenyl)ethanol (**13S-G2**) with high GR affinity demonstrated cytotoxicity *in vitro* at concentrations of 50–70 µM.

Overall, our findings indicate a high anti-proliferative activity of the synephrine derivatives 1-[4-(benzyloxy)phenyl]-2-(hexylamino)ethanol (**10S-E2**) and 2-(hexylamino)-1-(4-nitrophenyl)ethanol (**13S-G2**) and their possible application for anti-cancer treatment strategies that employ SEGRA class drugs.

## Figures and Tables

**Table 1:** The cytotoxic effects of the synephrine derivatives in K562 and Granta cells after 24 and 72 h incubations.

Code	IC_50_, µM			
	K562		Granta	
	24 h	72 h	24 h	72 h
4S-C2	N/I	N/I	201 ± 32.1	N/I
8S-E3	120 ± 20.4	N/I	89.1 ± 22.41	N/I
10S-E2	13.1 ± 1.51	N/I	13.0 ± 0.73	N/I
12S-B2	N/I	77.3 ± 34.1	246 ± 6.4	53.1 ± 18.92
13S-G2	184 ± 91.2	36.0 ± 14.0	26.8 ± 1.24	N/I
20S-E5	N/I	31.0 ± 0.4	N/I	N/I
21S-E6	N/I	130 ± 30	725 ± 392.3	80.1 ± 41.73
26S-F2	670 ± 148.0	56.1 ± 0.93	215 ± 71.1	108 ± 14.4

N/I—no inhibition

**Table 2:** The *in silico* synephrine derivatives’ affinity for the GR.

Compound	MolDock Score	H-Bond	Compound	MolDock Score	H-Bond
**Dex**	−146.169	Asn564, Thr739, Tyr735, Gln642, Arg611	**19S-C6**	−102.139	Asn564, Gln570, Arg611, Leu563
**10S-E2**	−142.988	Asn564, Thr739, Gln642	**9S-G1**	−99.2892	Gln570, Arg611
**21S-E6**	−136.472	Asn564, Thr739, Gln642, Leu732	**18S-C5**	−99.2424	Asn564, Gln570, Arg611, Met560
**8S-E3**	−125.395	Asn564, Thr739, Gln642, Leu732	**17S-B6**	−95.9832	Asn564, Leu732, Cys643
**20S-E5**	−123.111	Thr739, Gln642	**25S-F6**	−95.955	Asn564, Leu563, Gln570
**7S-E1**	−118.911	Thr739, Leu732	**5S-C3**	−95.2164	Thr739, Cys643, Leu732
**13S-G2**	−117.383	Asn564, Gln642, Arg611	**3S-C1**	−94.9736	Thr739, Leu732
**11S-E4**	−116.985	Thr739, Tyr735, Gln642	**6S-C4**	−94.1991	Met604, Gln570, Arg611, Leu563
**4S-C2**	−106.142	Thr739	**24S-F5**	−93.3857	Asn564, Gln642, Cys643
**26S-F2**	−105.588	Asn564, Thr739, Leu732	**2S-B3**	−90.1894	Asn564, Cys643
**12S-B2**	−103.893	Asn564, Thr739, Gln642	**15S-F1**	−85.8472	Asn564, Met604
**23S-G6**	−103.408	Thr739, Gln642, Cys643, Leu732	**16S-F3**	−89.1399	Asn564, Leu732, Cys643
**14S-G3**	−102.897	Asn564, Gln570, Arg611, Cys736	**27S-B5**	−89.0255	Asn564, Gln570, Arg611

## Data Availability

Data are contained within the article and Supplementary Materials.

## Supplementary Materials

The following supporting information can be downloaded at: https://www.mdpi.com/article/10.3390/biom15010002/s1, Figures S1–S52: copies of NMR spectra; Figure S53: Structure of the GR active site with analogue of synephrine; Table S1: Synephrine derivatives affinity for GR *in silico*; Table S2: Calculated lipophilicity of structures.
