# Supplementary material for: Synthesis and Anti-Cancer Activity In Vitro of Synephrine Derivatives
Source: Biomolecules. 2024 Dec 24;15(1):2. doi: 10.3390/biom15010002 (PMC11762542; doi:10.3390/biom15010002)
Supplement: Supplementary file 1 [file biomolecules-15-00002-s001.zip › biomolecules-3286022-supplementary.pdf]

Synthesis and anti-cancer activity *in vitro* of synephrine derivatives

Ekaterina M. Zhidkova [1], Evgeniya S. Oleynik [2], Ekaterina A. Mikhina [2], Daria V. Stepanycheva [1], Diana D. Grigoreva [1], Lyubov E. Grebenkina [2], Kirill V. Gordeev [3], Ekaterina D. Savina [2], Andrey V. Matveev [2], Marianna G. Yakubovskaya [1,4] and Ekaterina A. Lesovaya [1,4,5*]

Supplementary Material

Table S1. Synephrine derivatives affinity to GR *in silico*

Structure, name	Code	MolDock Score	Structure	Code	MolDock Score
	Dex	-146.169		Syn	-75.4565
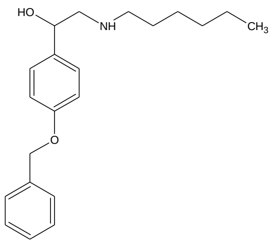	10S-E2	-142.988	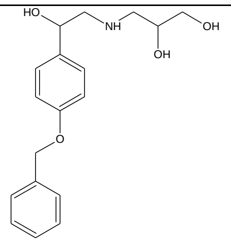	21S-E6	-136.472
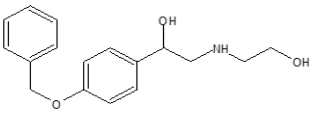	8S-E3	-125.395	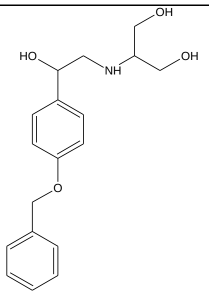	20S-E5	-123.111
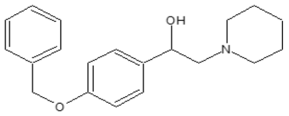	7S-E1	-118.911	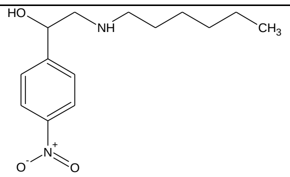	13S-G2	-117.383
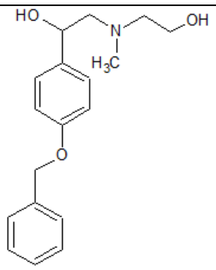	11S-E4	-116.985	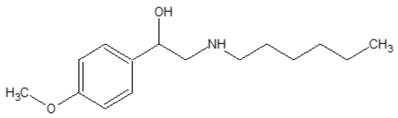	4S-C2	-106.142
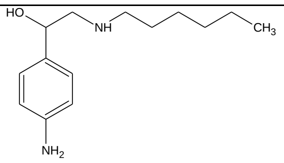	26S-F2	-105.588	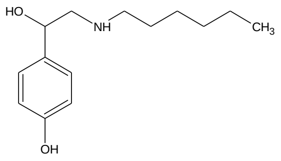	12S-B2	-103.893

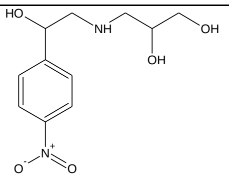	23S-G6	-103.408	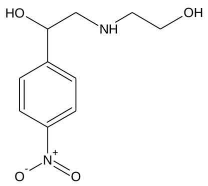	14S-G3	-102.897
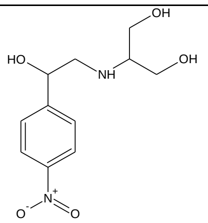	22S-G5	-103.044	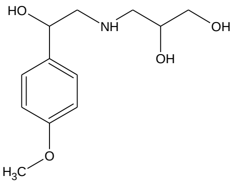	19S-C6	-102.139
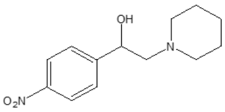	9S-G1	-99.2892	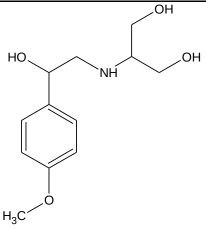	18S-C5	-99.2424
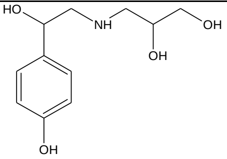	17S-B6	-95.9832	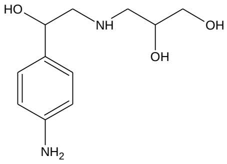	25S-F6	-95.955
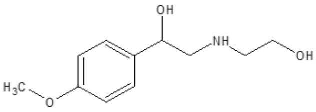	5S-C3	-95.2164	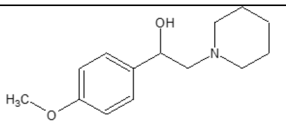	3S-C1	-94.9736
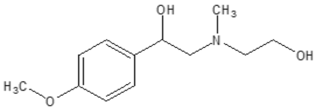	6S-C4	-94.1991	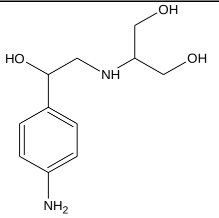	24S-F5	-93.3857
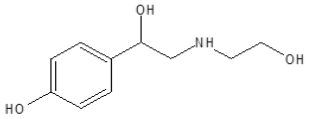	2S-B3	-90.1894	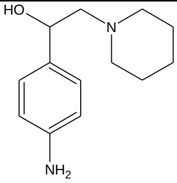	15S-F1	-85.8472
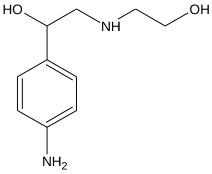	16S-F3	-89.1399	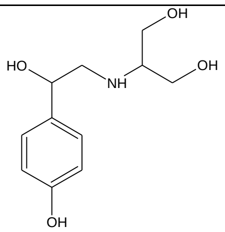	27S-B5	-89.0255

NMR spectra copies:

1-(4-methoxyphenyl)-2-piperedine-1-yl ethanol (3S-C1)

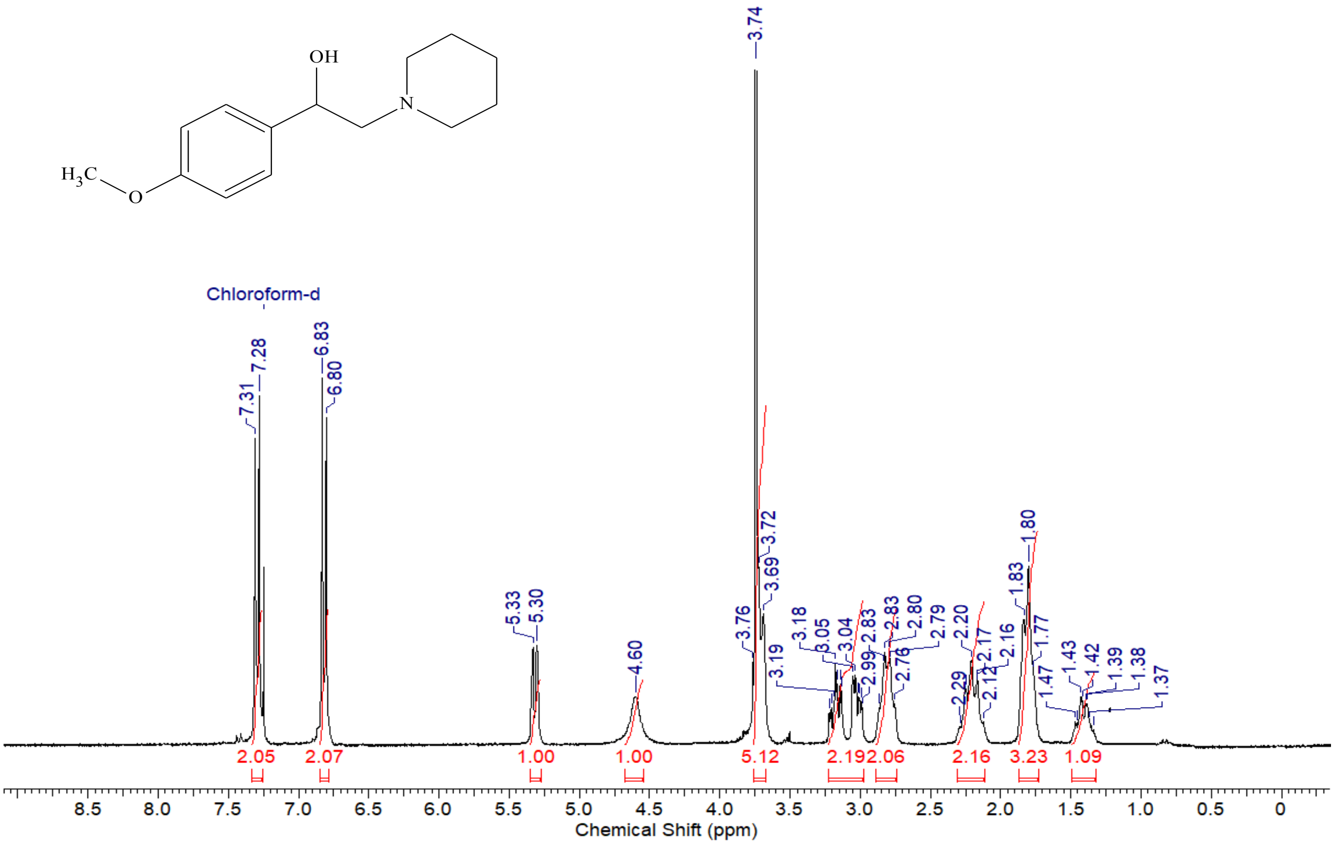

Figure S1. ^{1}H NMR spectrum **3S-C1**

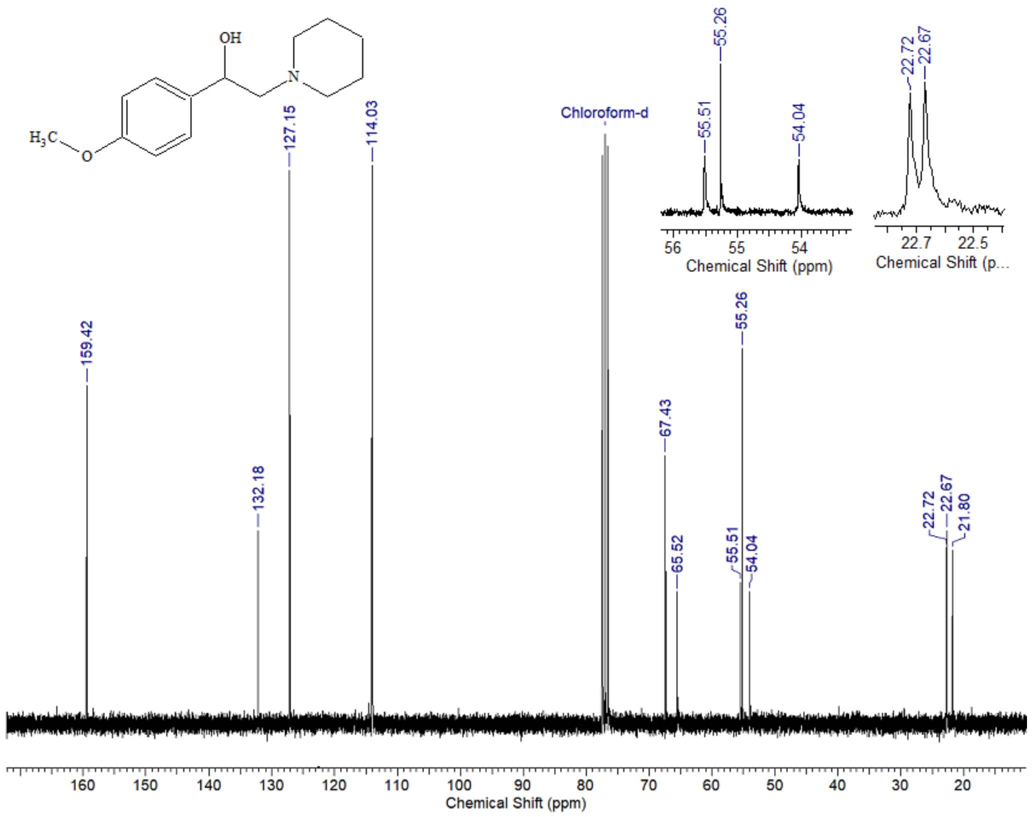

Figure S2. ^{13}C NMR spectrum **3S-C1**

2-(2-hydroxyethyl)(methyl) amino)-1-(4-methoxyphenyl)ethanol (6S-C4)

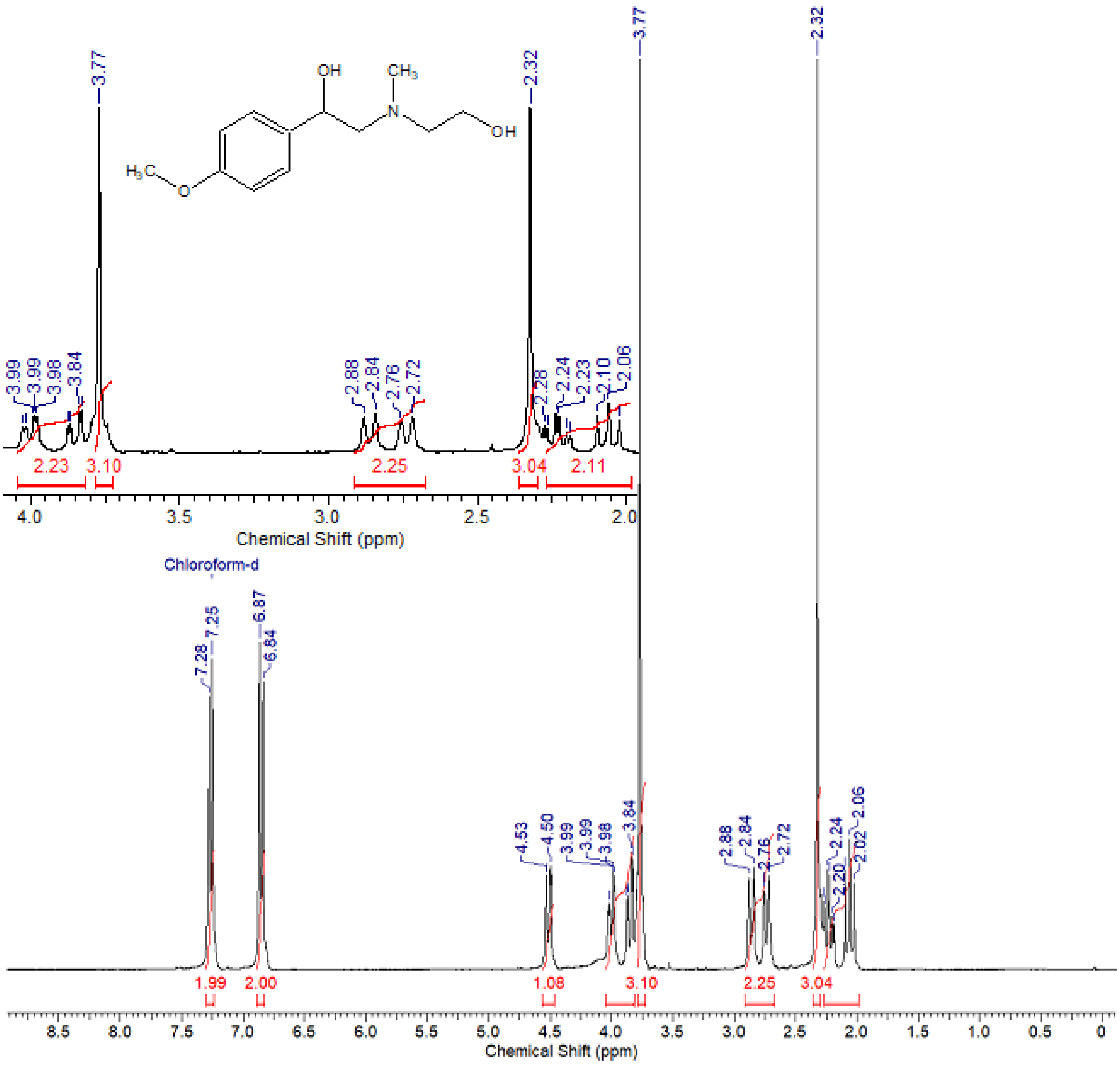

Figure S3. ^{1}H NMR spectrum **6S-C4**

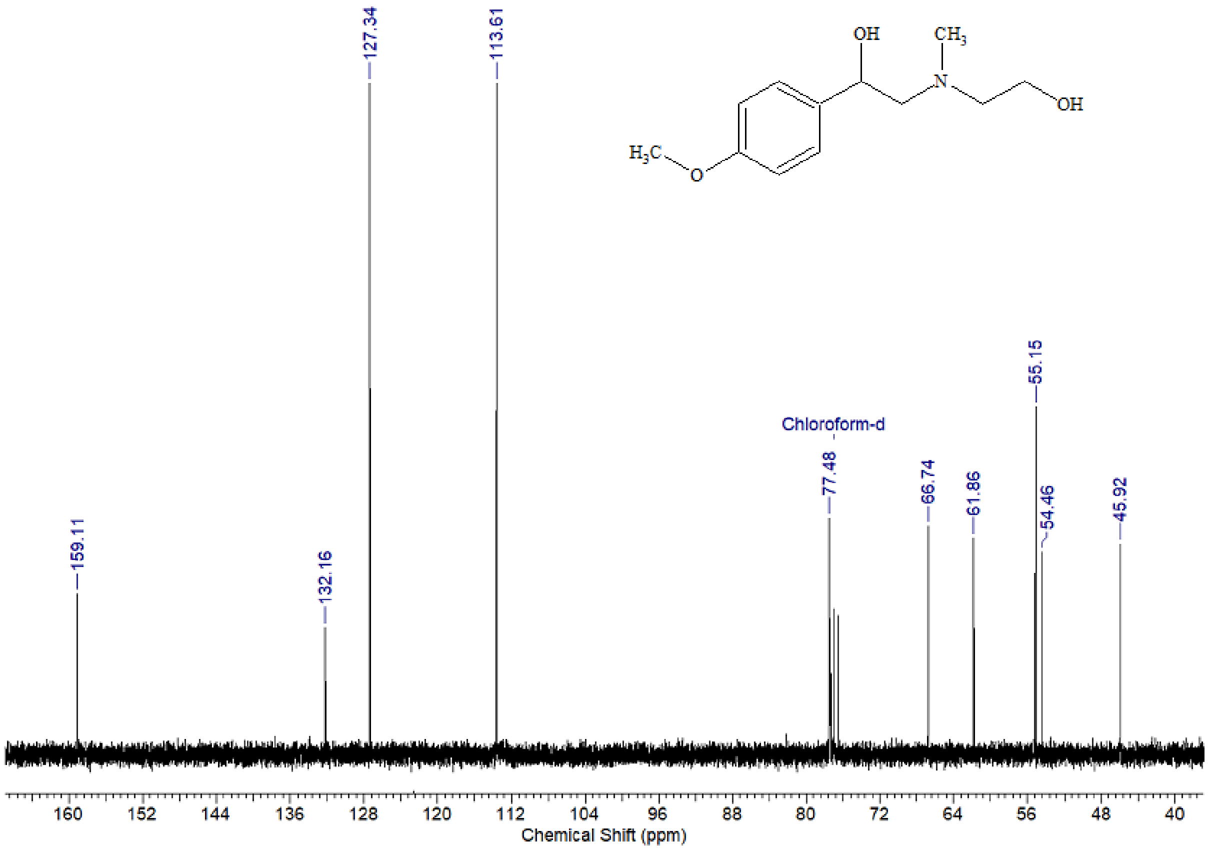

Figure S4. ^{13}C NMR spectrum **6S-C4**

1-(4-(benzyloxy) phenyl)-2-piperidine-1-yl-ethanol (7S-E1)

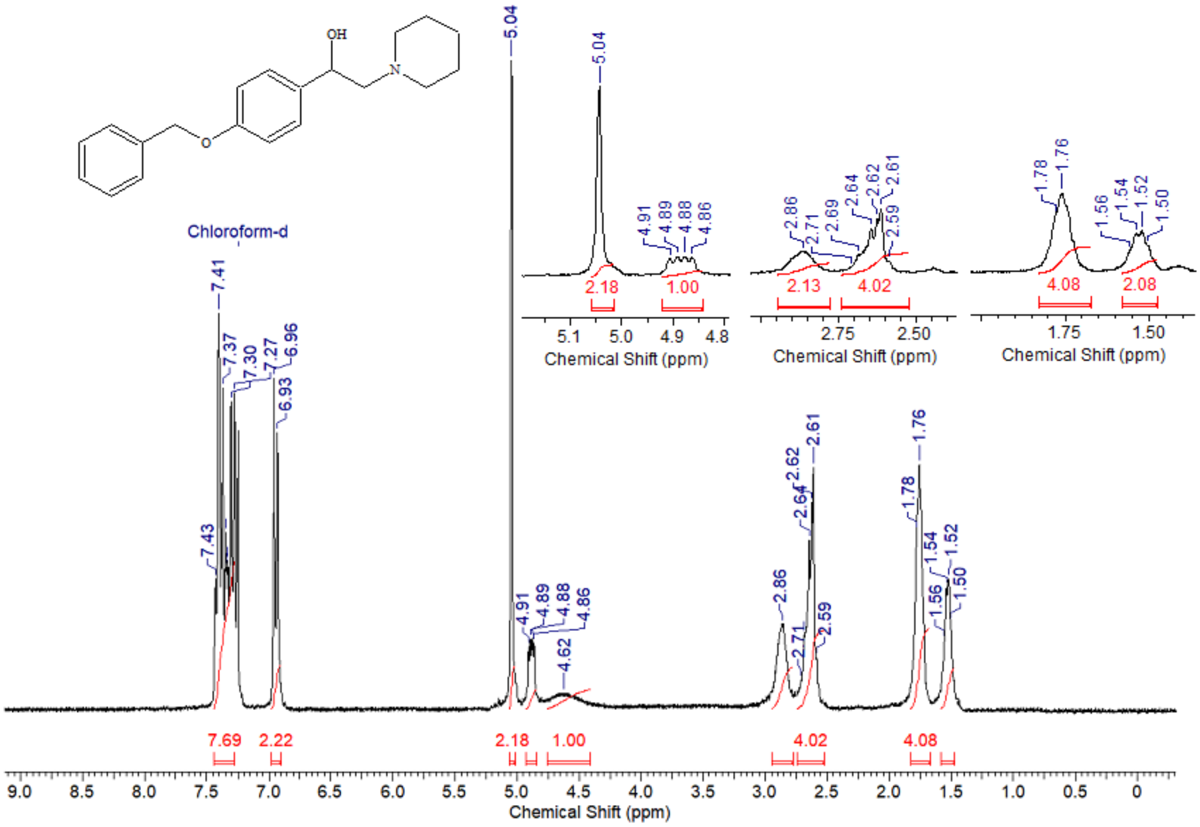

Figure S5. ¹H NMR spectrum **7S-E1**

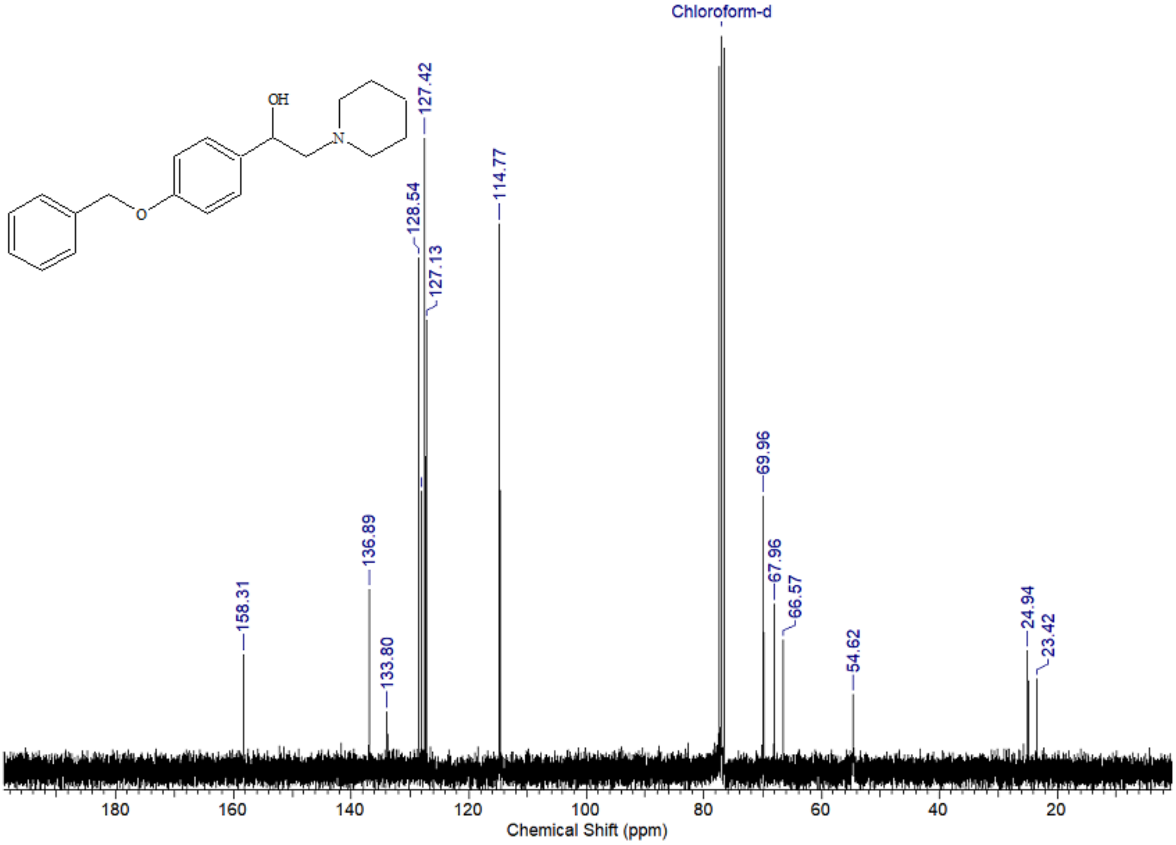

Figure S6. ^{13}C NMR spectrum **7S-E1**

1-(4-(benzyloxy)phenyl)-2-((2-hydroxyethyl)(methyl)amino)ethanol (11S-E4)

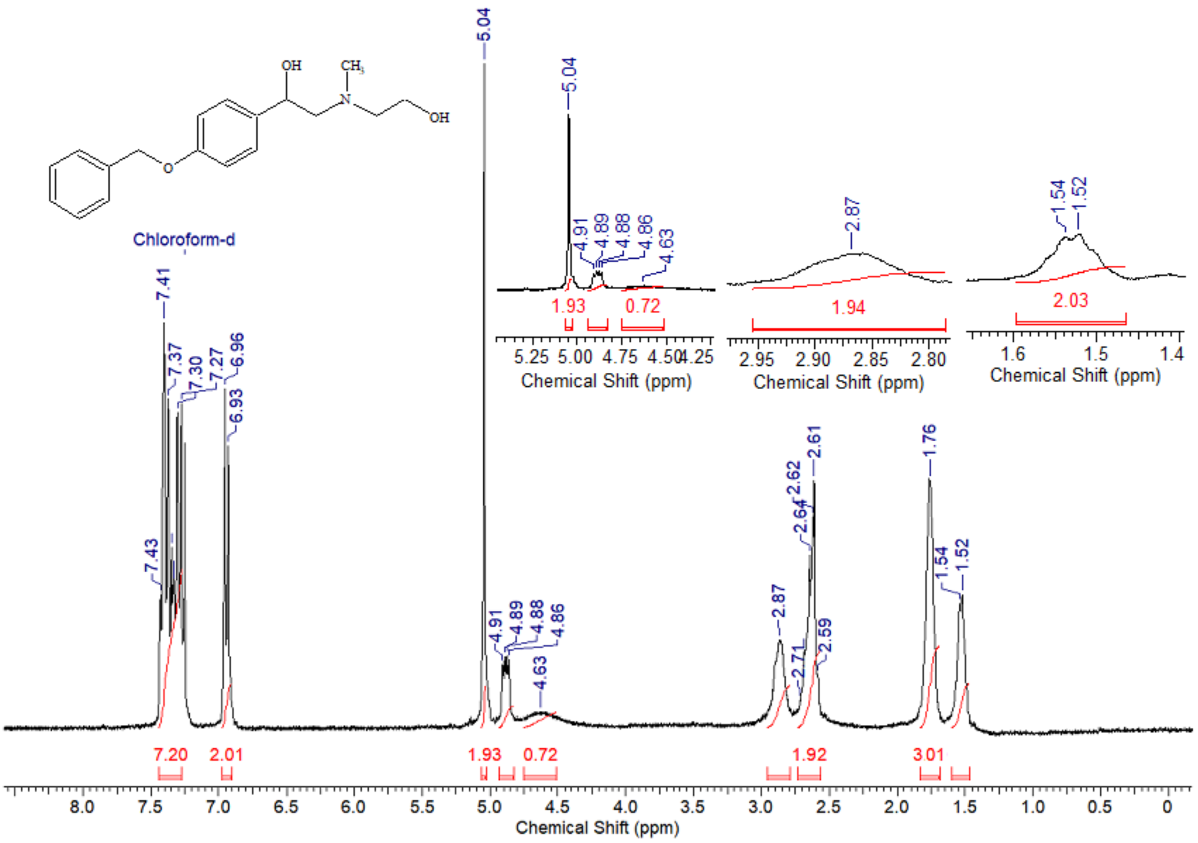

Figure S7. ^{1}H NMR spectrum **11S-E4**

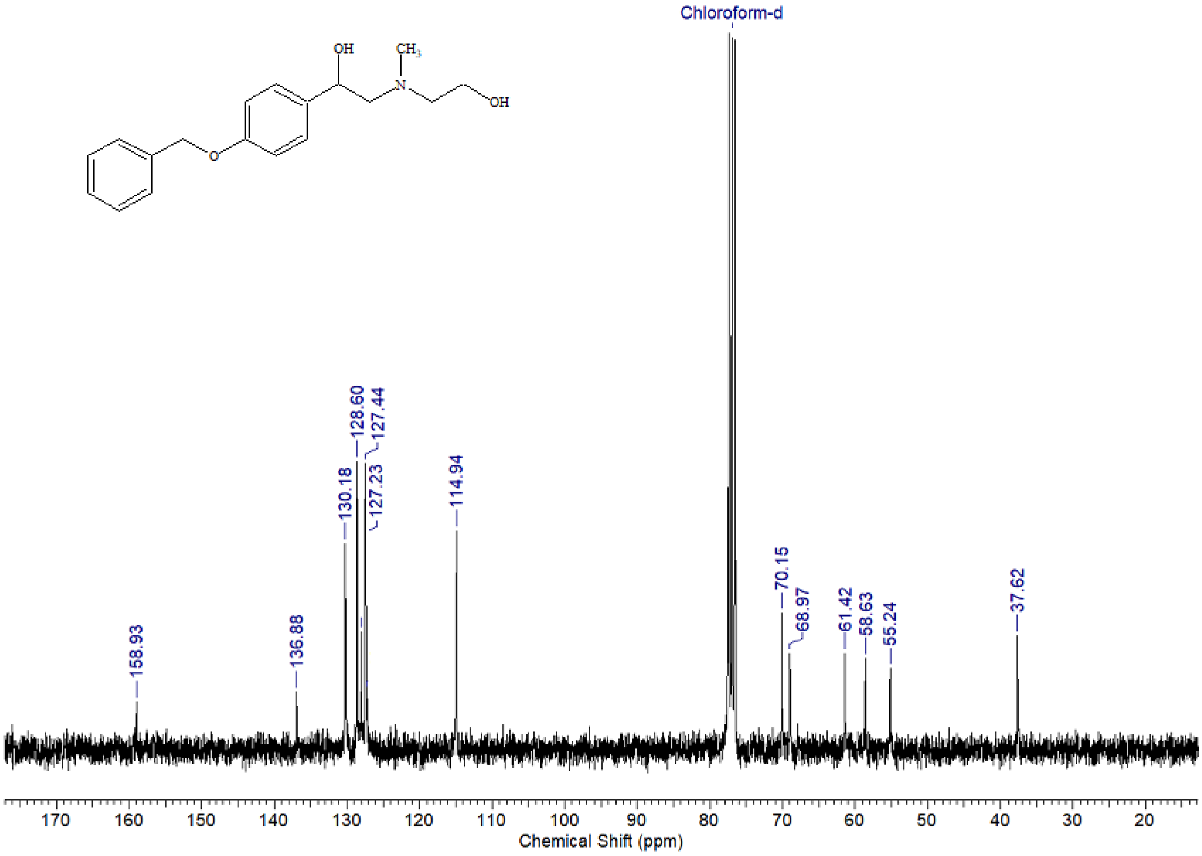

Figure S8. ^{13}C NMR spectrum **11S-E4**

2-(hexylamino)-1-(4-methoxyphenyl)ethanol (4S-C2)

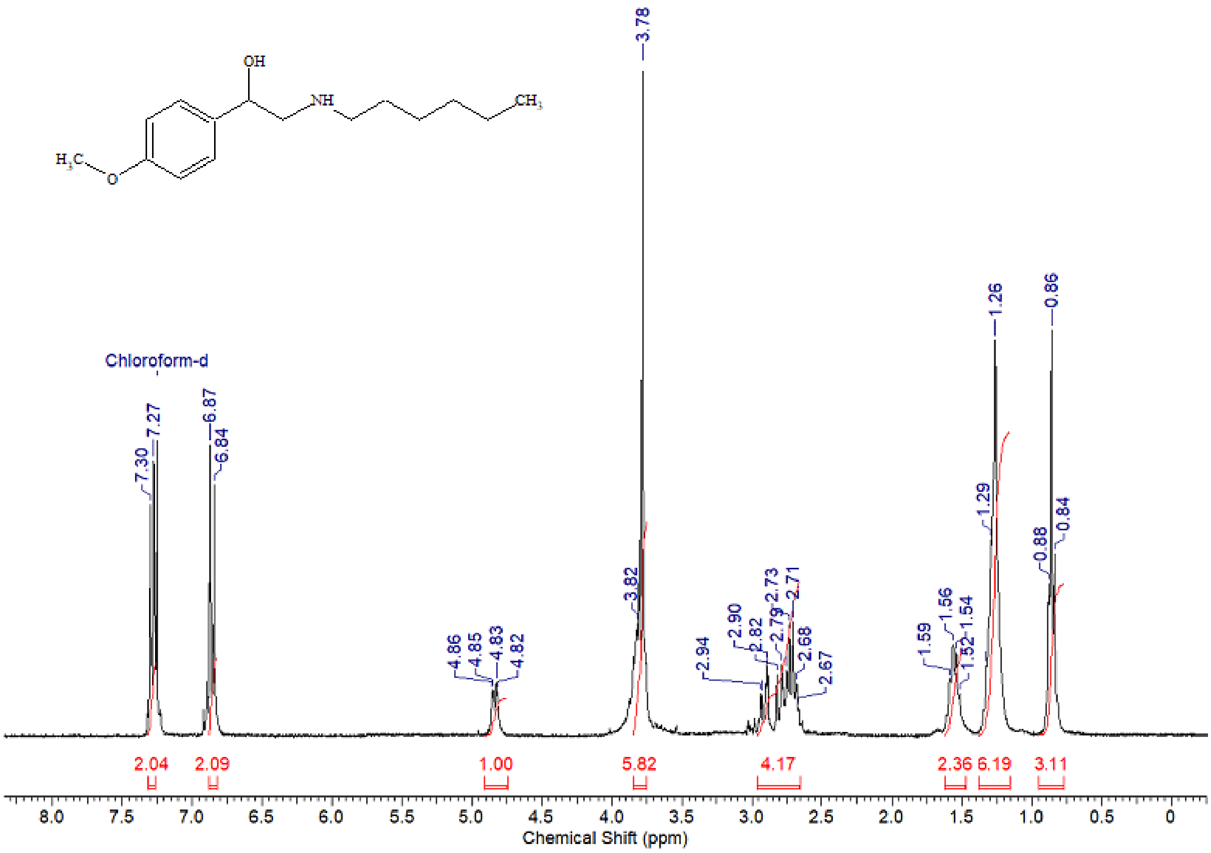

Figure S9. ^{1}H NMR spectrum **4S-C2**

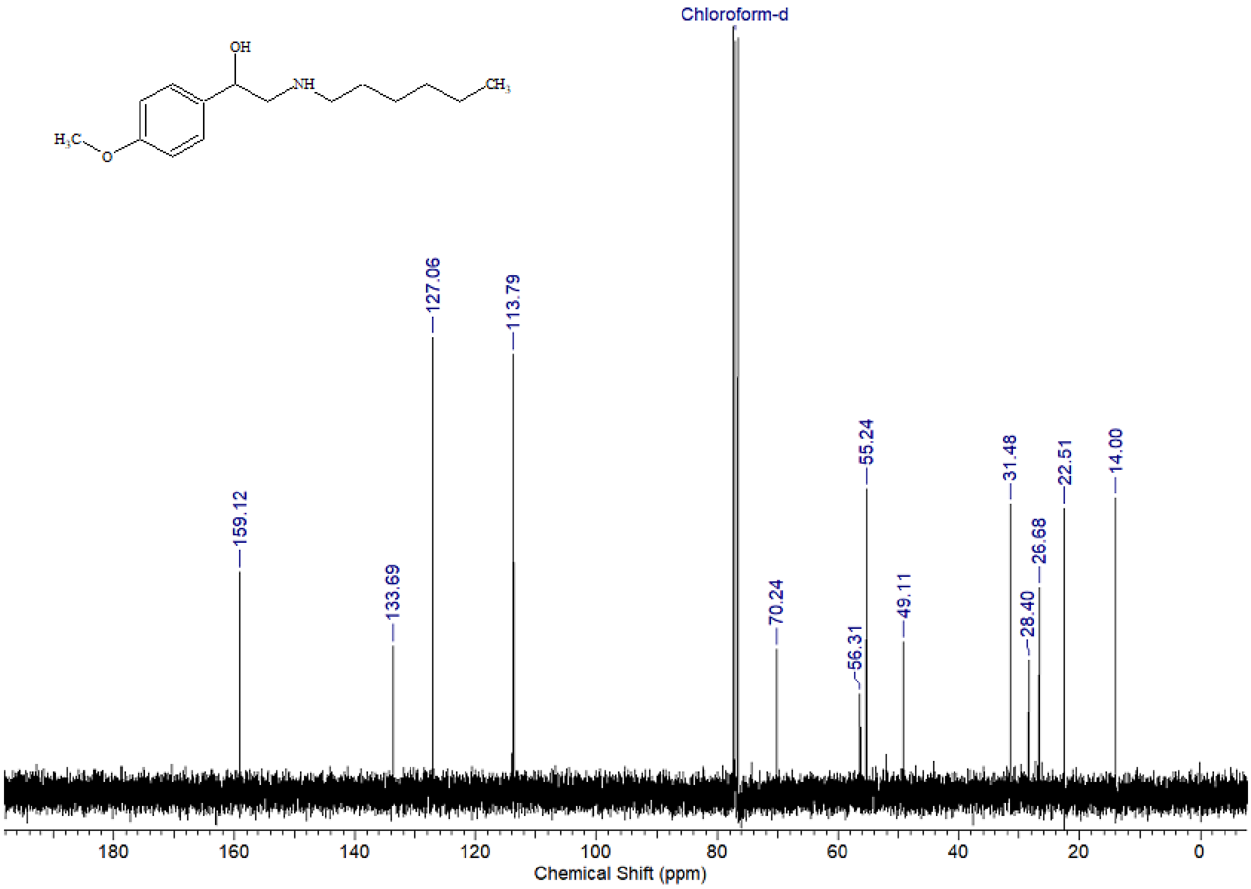

Figure S10. ^{13}C NMR spectrum **4S-C2**

2-((2-hydroxyethyl)amino)-1-(4-methoxyphenyl)ethanol (5S-C3)

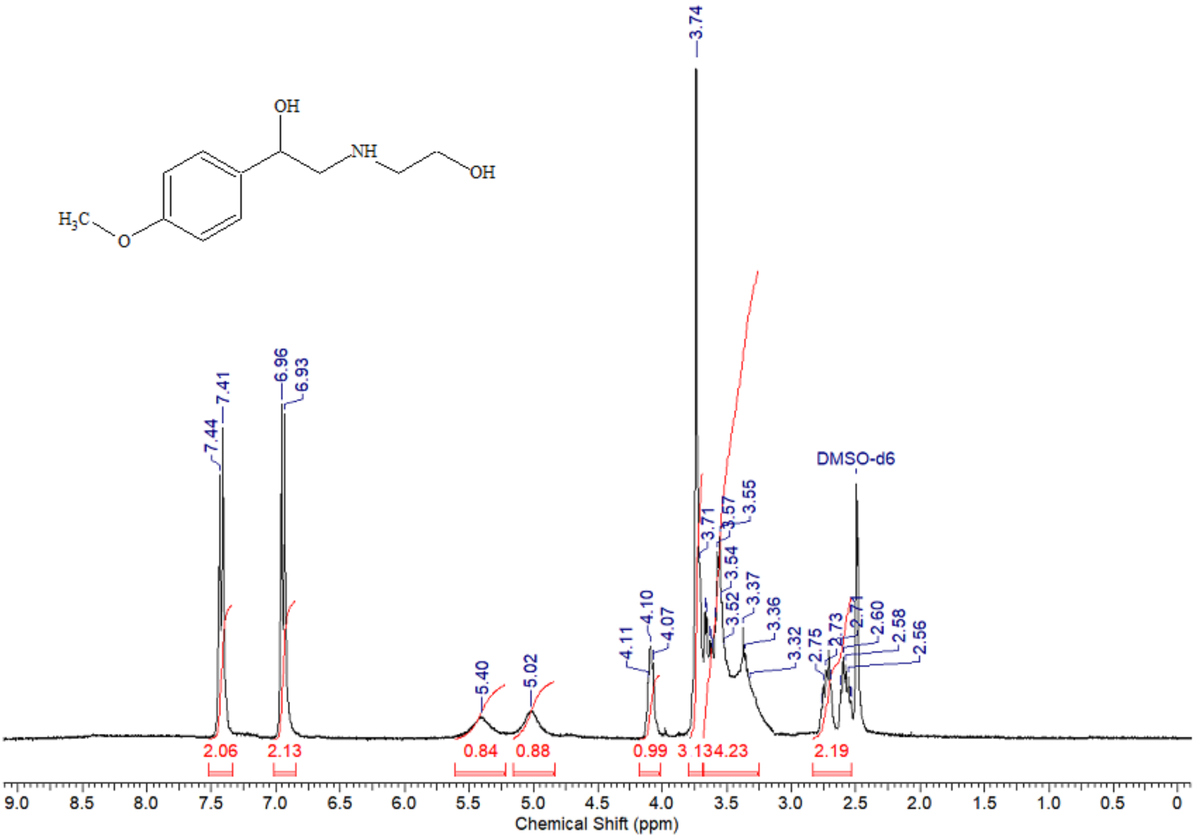

Figure S11. ^{1}H NMR spectrum **5S-C3**

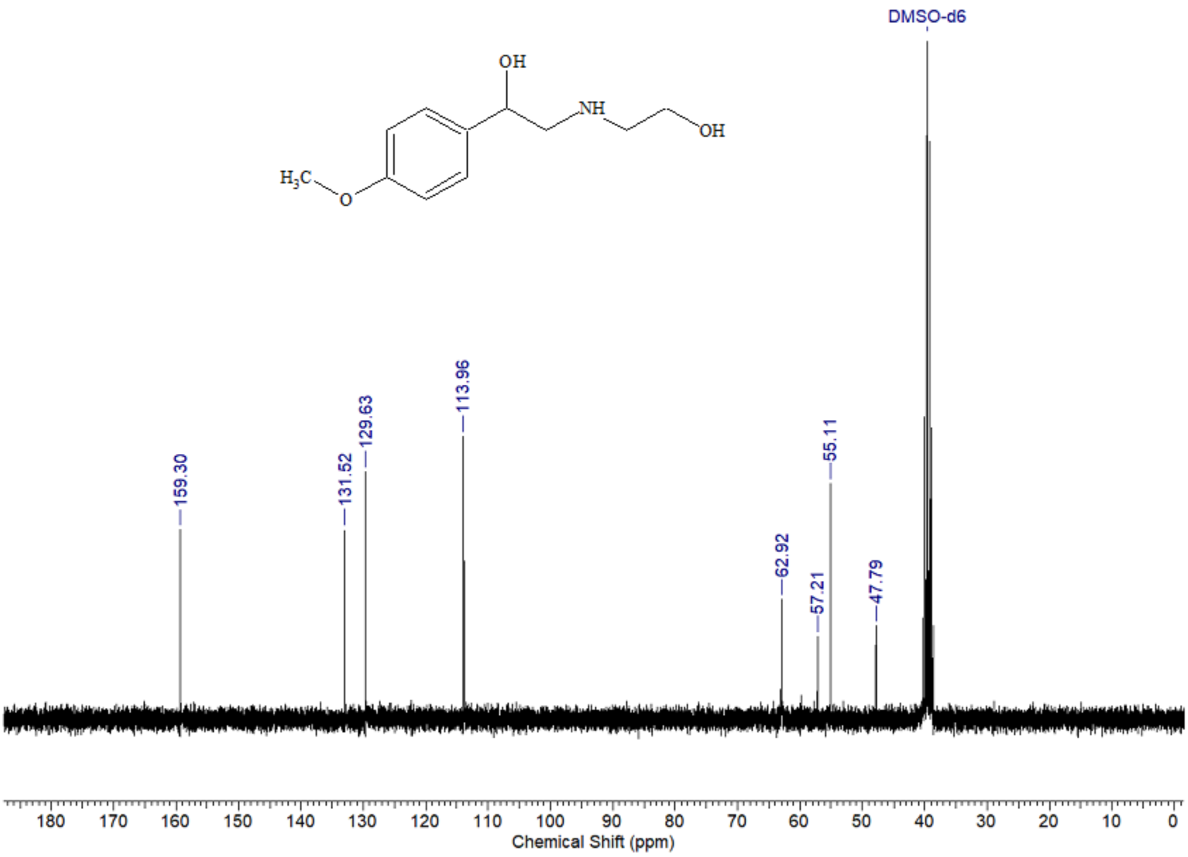

Figure S12. ^{13}C NMR spectrum **5S-C3**

2-((2-hydroxy-2-(4-methoxyphenyl)ethyl)amino)propane-1,3-diol (18S-C5)

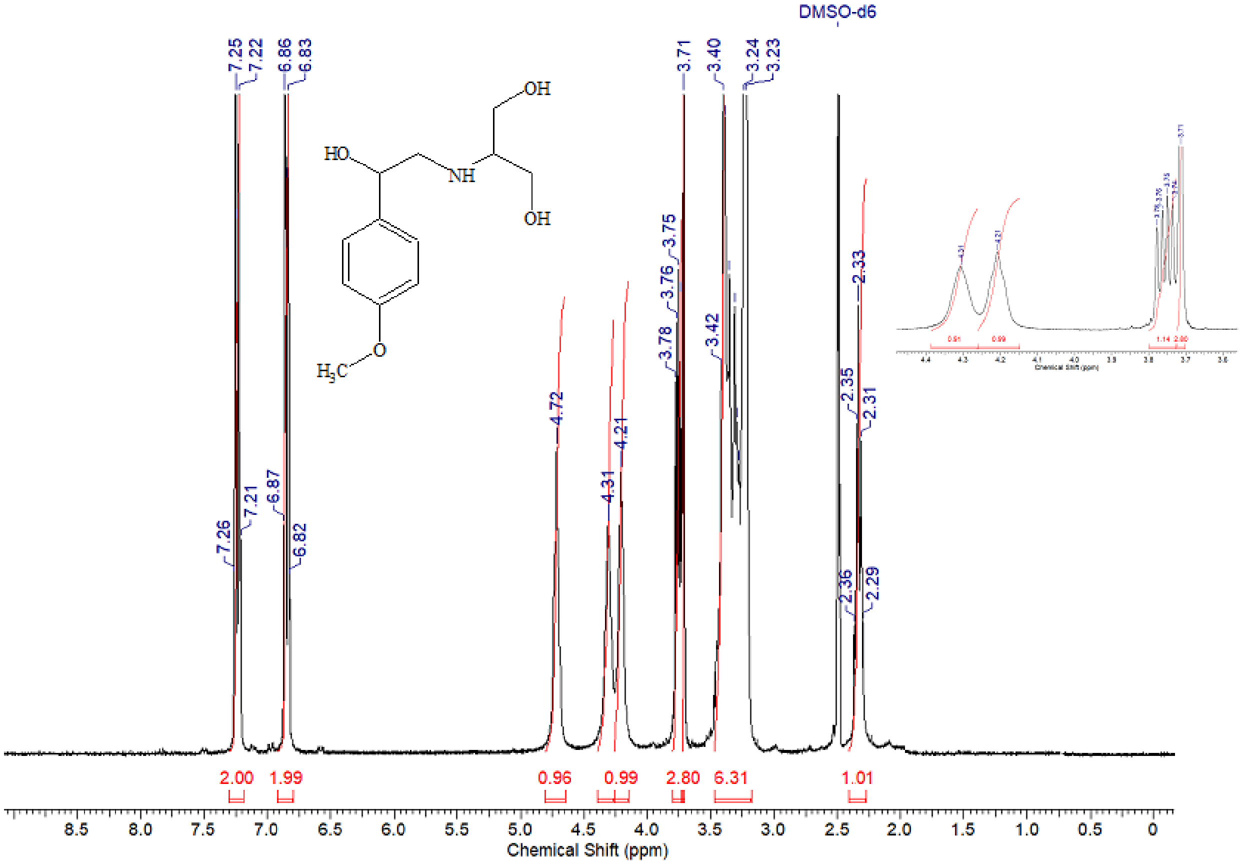

Figure S13. ^{1}H NMR spectrum **18S-C5**

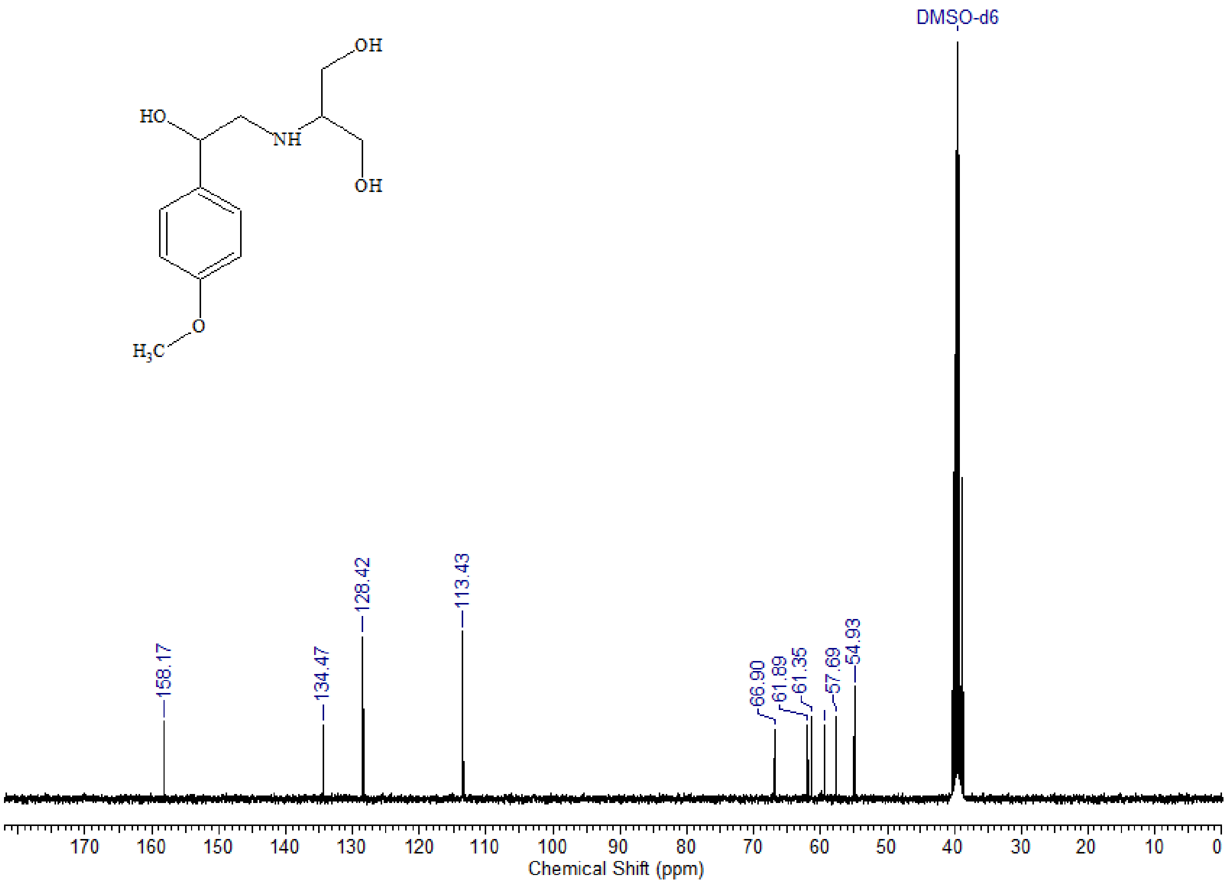

Figure S14. ^{13}C NMR spectrum **18S-C5**

3-((2-hydroxy-2-(4-methoxyphenyl)ethyl)amino)propane-1,2-diol (19S-C6)

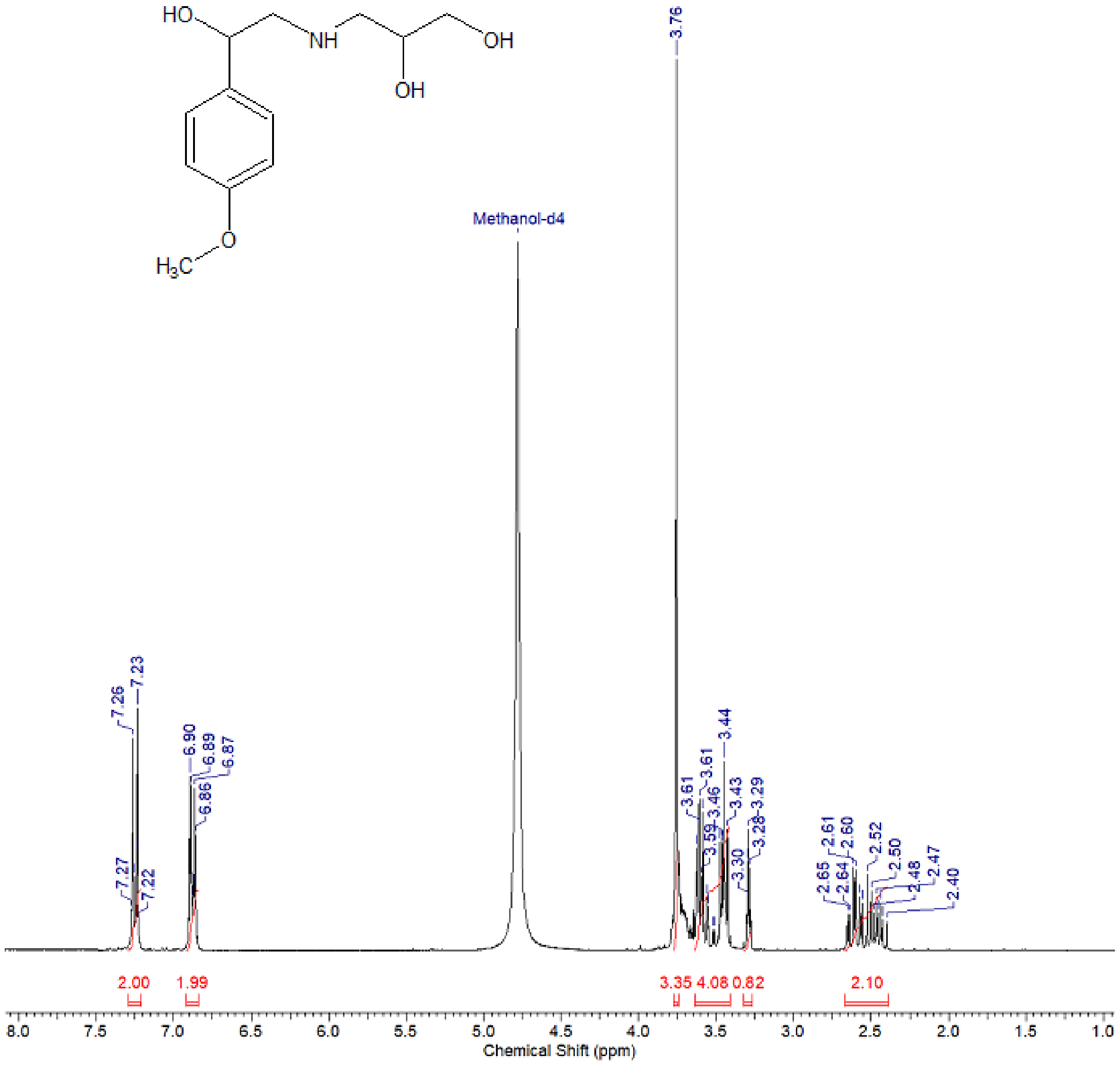

Figure S15. ^{1}H NMR spectrum **19S-C6**

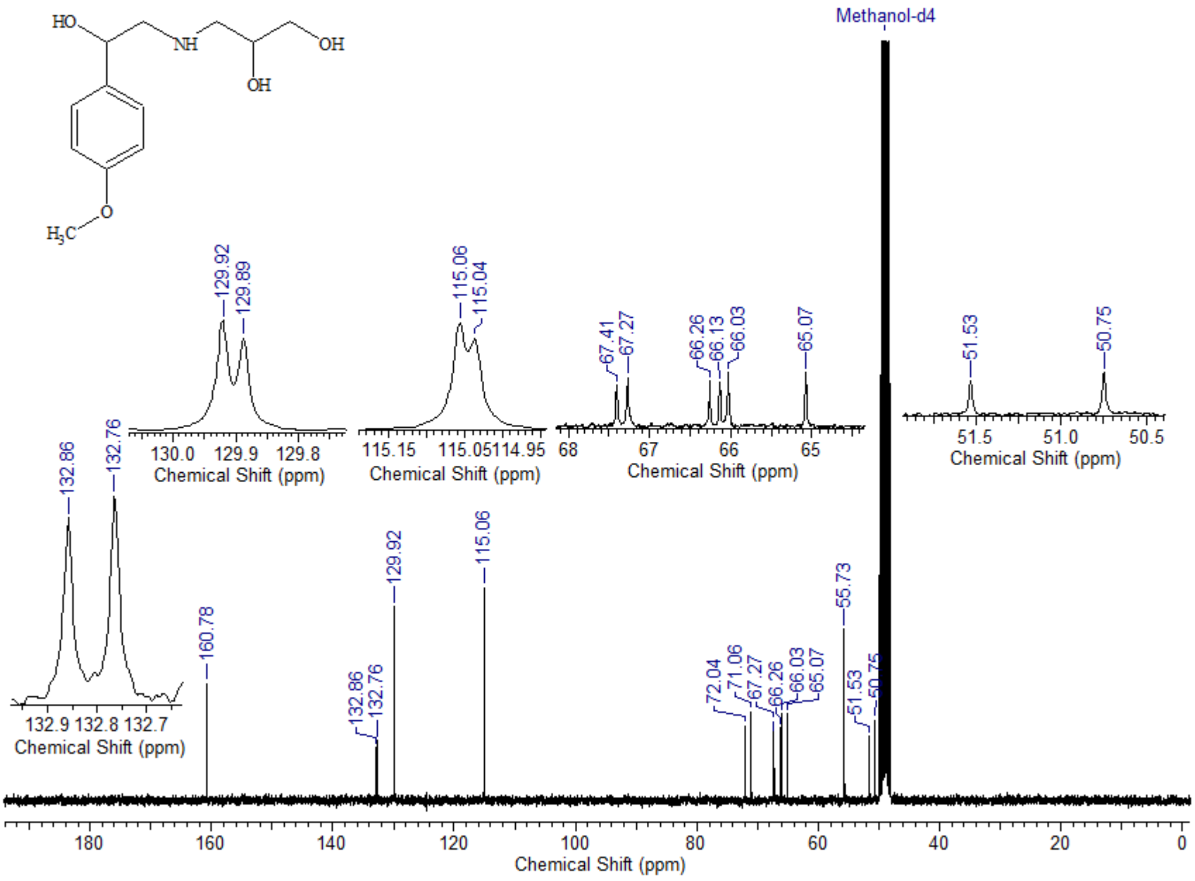

Figure S16. ^{13}C NMR spectrum **19S-C6**

1-(4-(benzyloxy)phenyl)-2(hexylamino)ethanol (10S-E2)

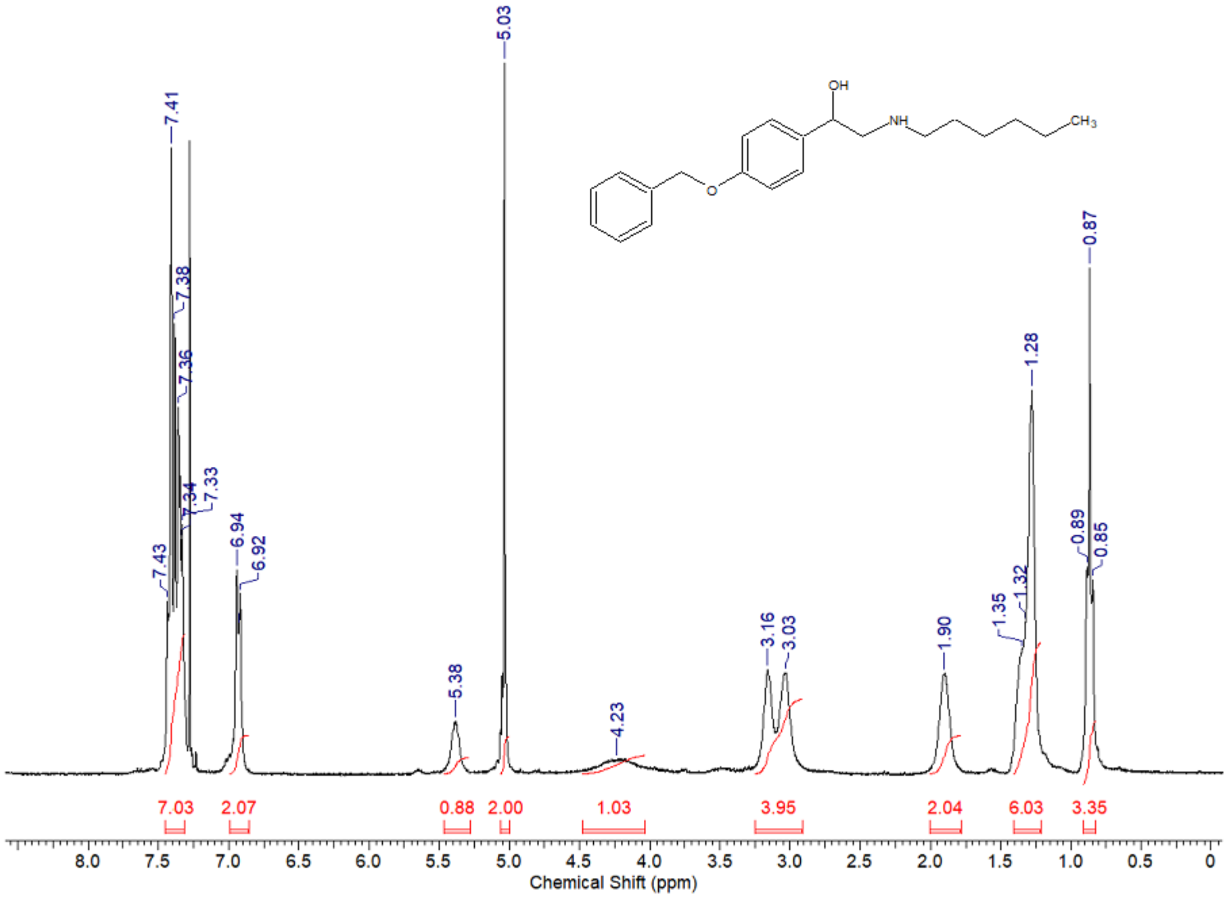

Figure S17. ^{1}H NMR spectrum **10S-E2**

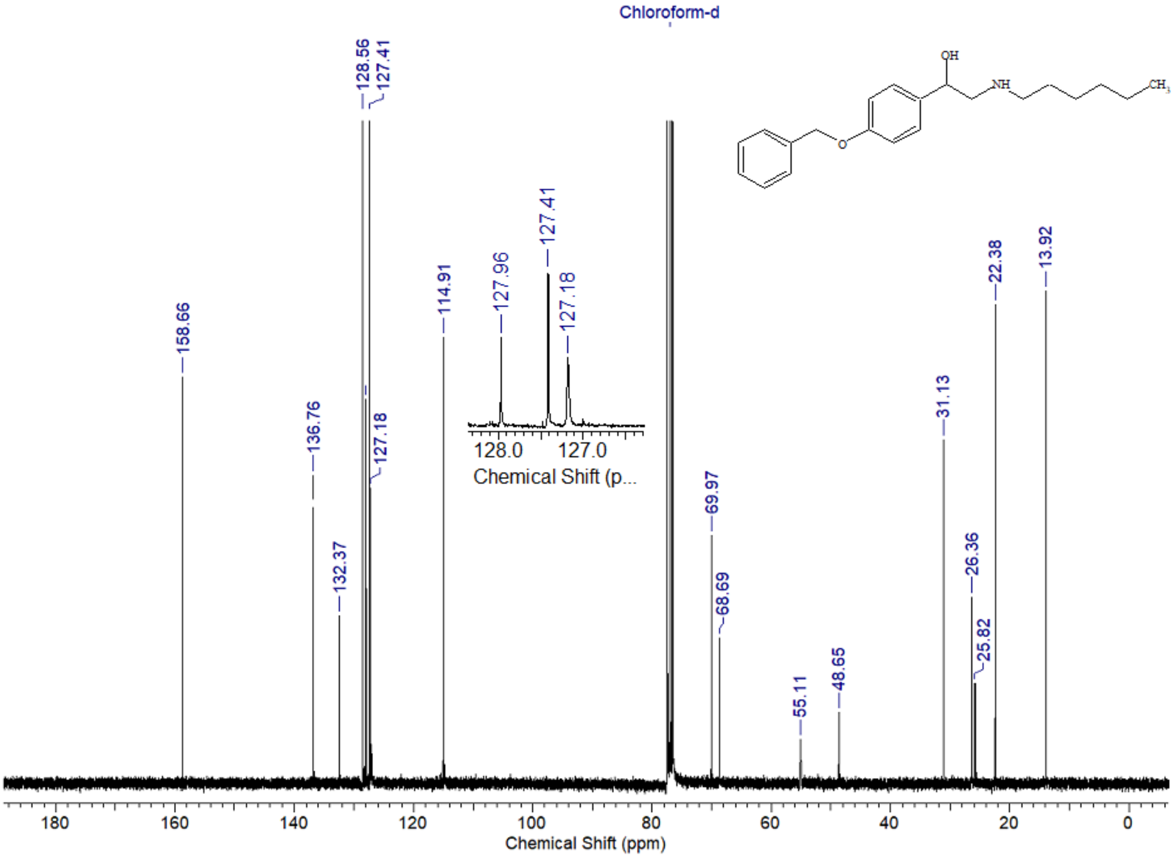

Figure S18. ^{13}C NMR spectrum **10S-E2**

1-(4-(benzyloxy)phenyl)-2-((2-hydroxyethyl)amino)ethanol (8S-E3)

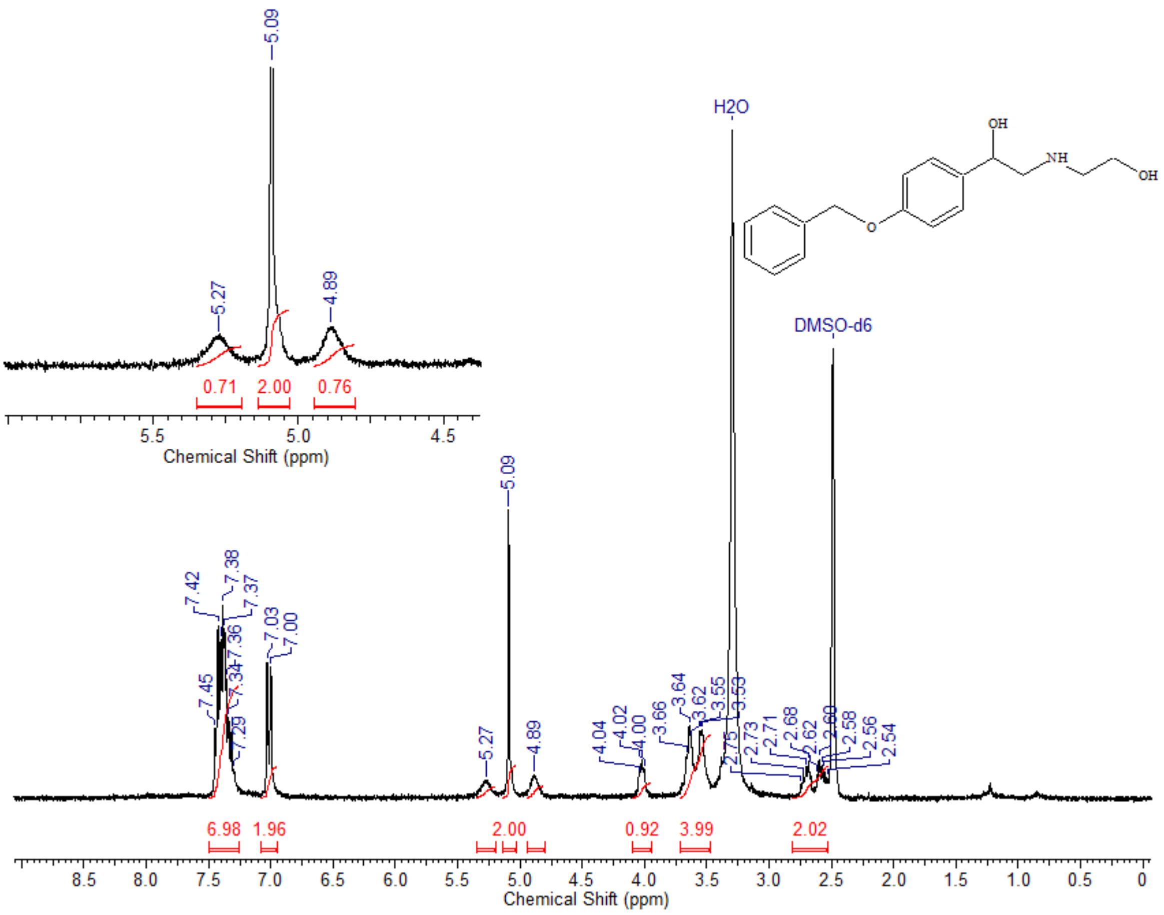

Figure S19. ^{1}H NMR spectrum **8S-E3**

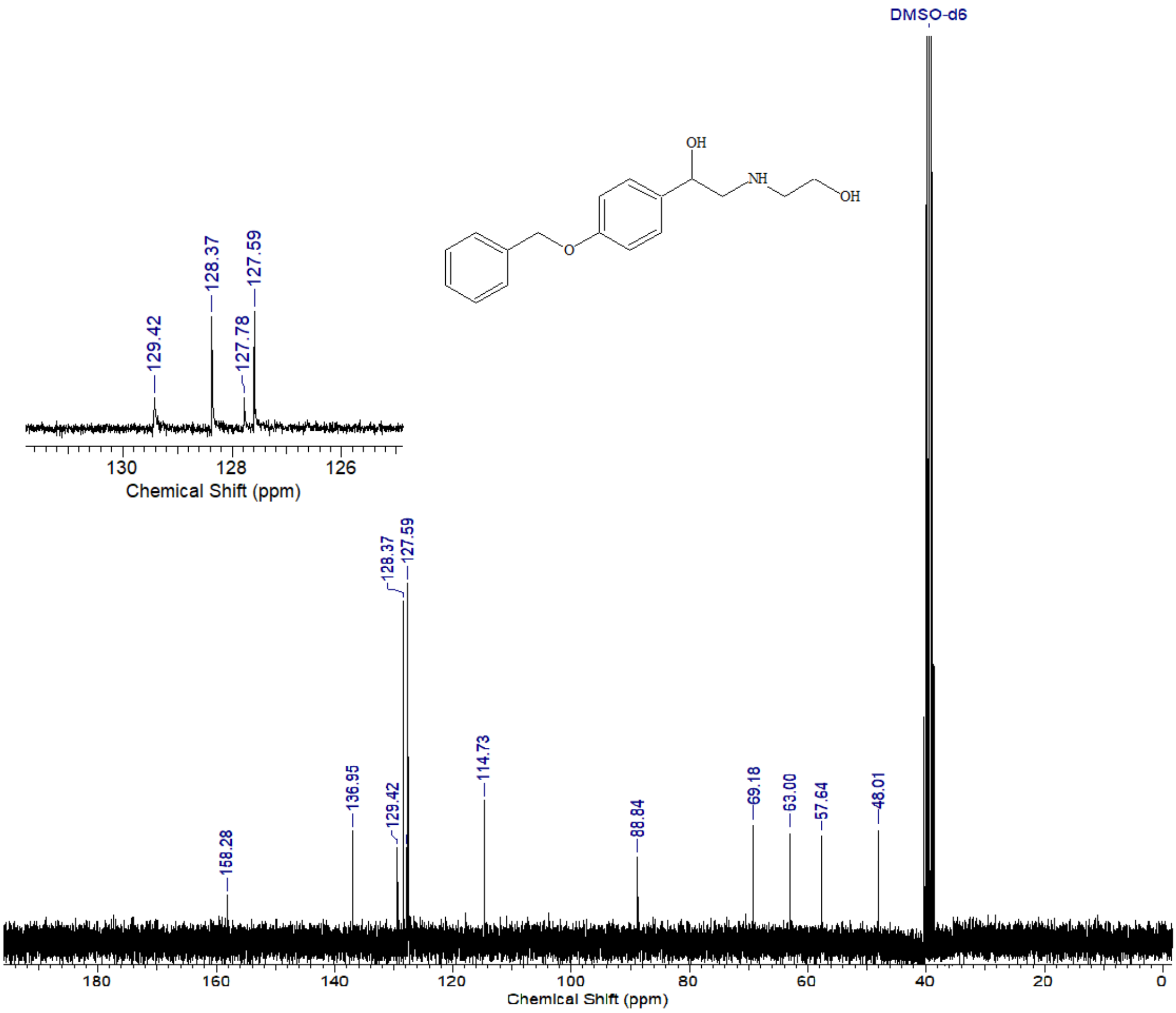

Figure S20. ^{13}C NMR spectrum **8S-E3**

2-((2-(4-(benzyloxy)phenyl)-2-hydroxyethyl)amino)propane-1,3-diol (20S-E5)

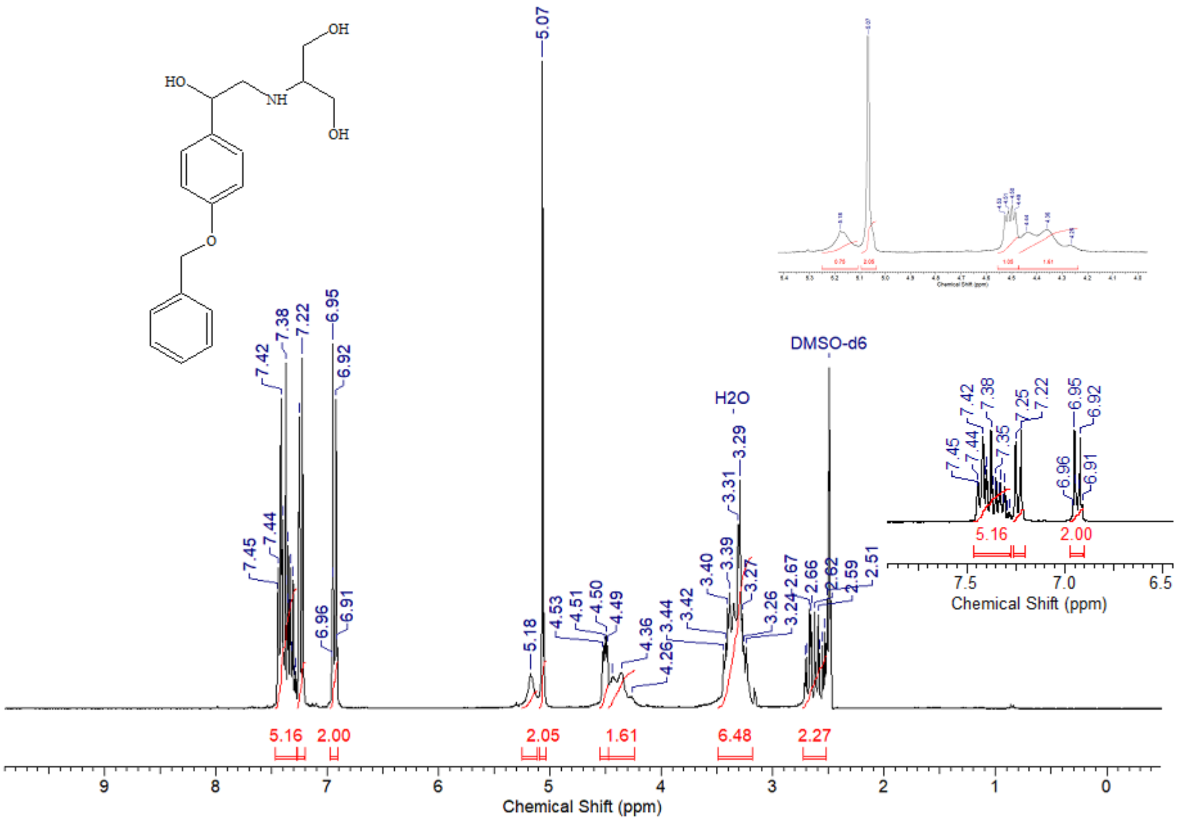

Figure S21. <u>^{1}H NMR spectrum **20S-E5**</u>

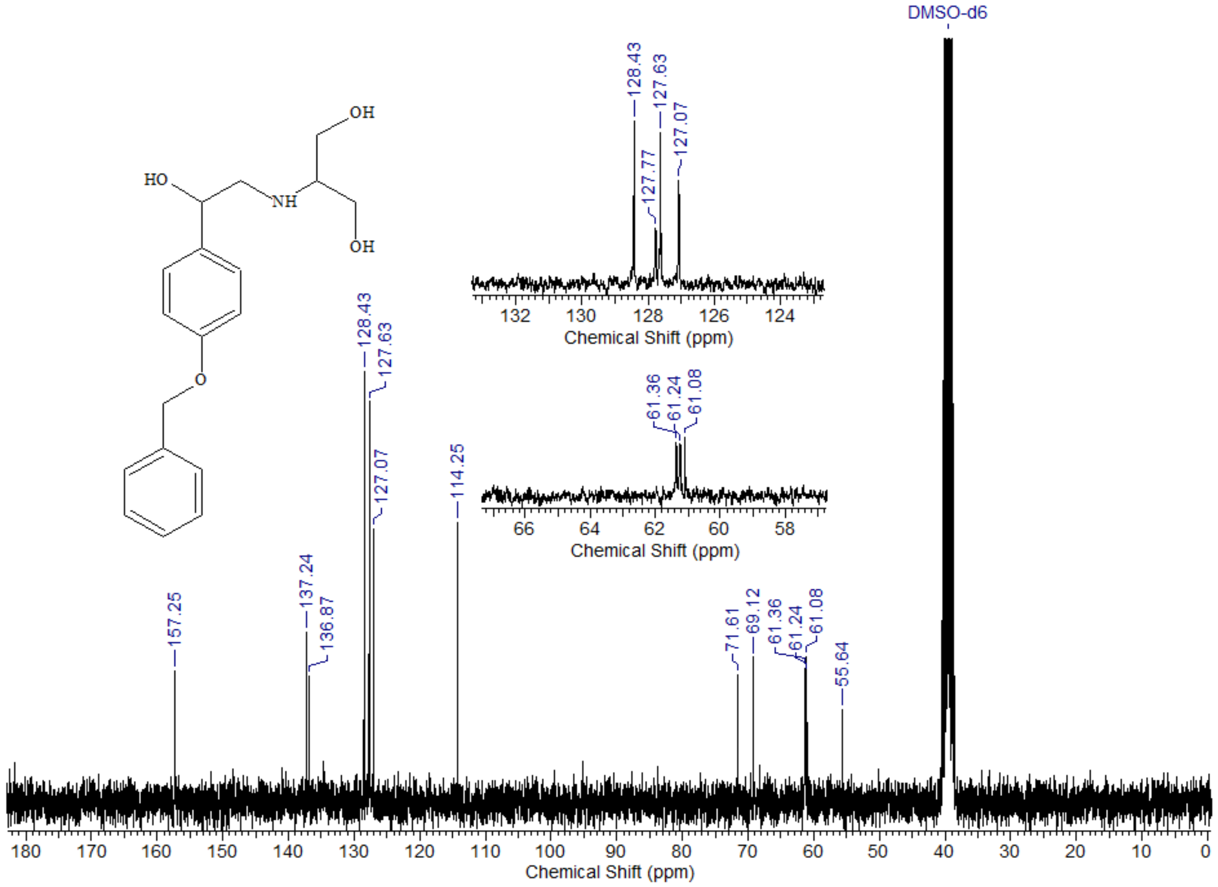

Figure S22<u>^{13}C NMR spectrum **20S-E5**</u>

3-((2-(4-(benzyloxy)phenyl)-2-hydroxyethyl)amino)propane-1,2-diol (21S-E6)

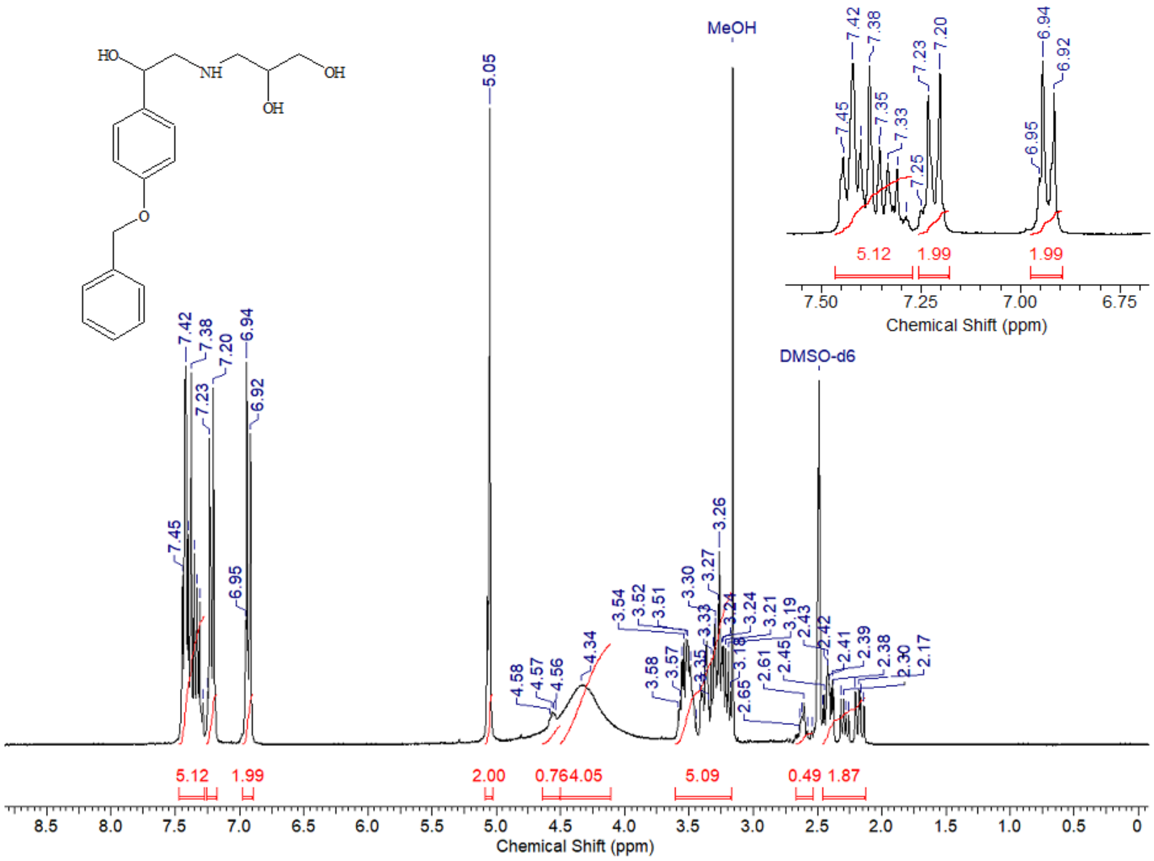

Figure S23. ^{1}H NMR spectrum **21S-E6**

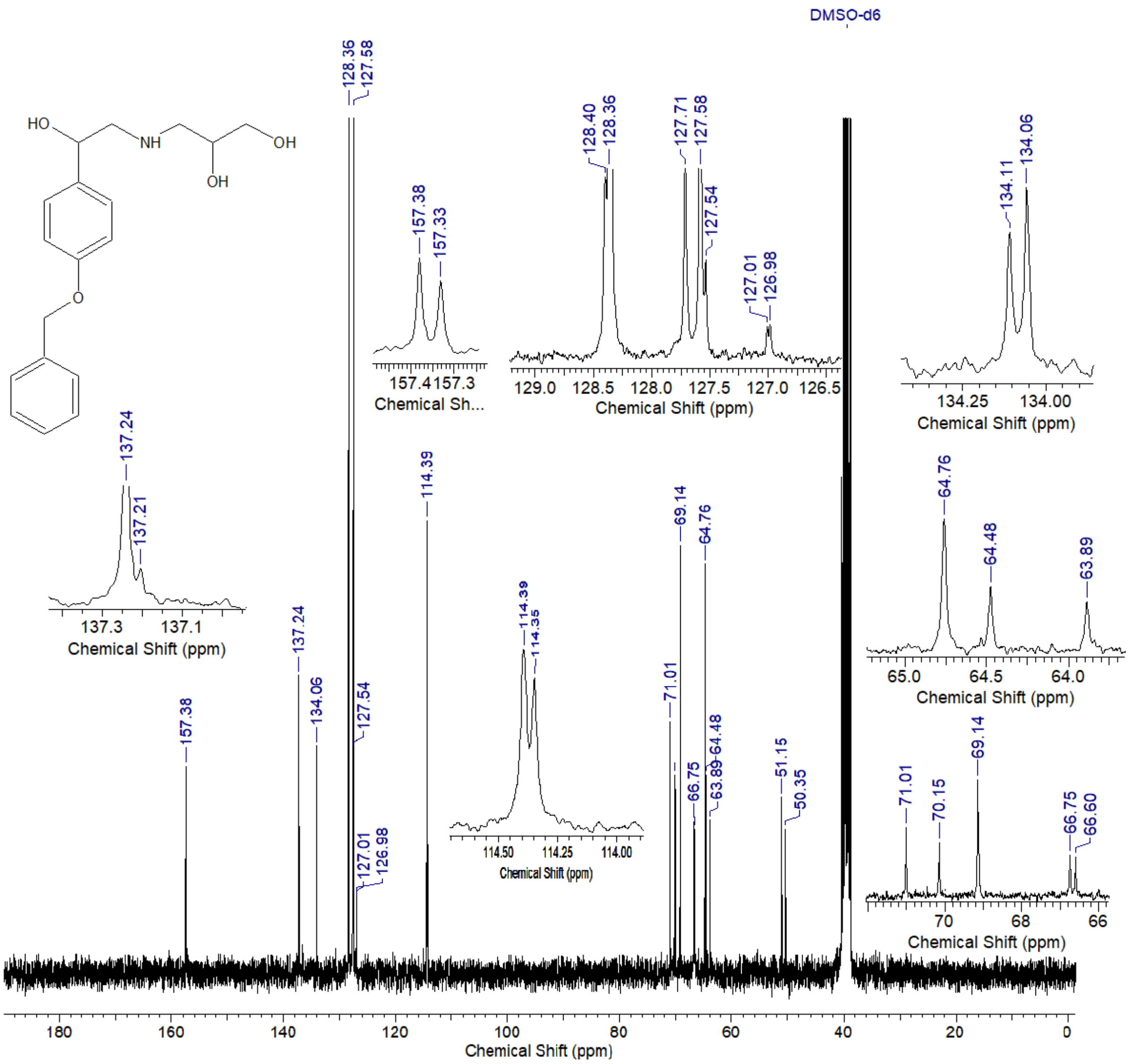

Figure S24. ^{13}C NMR spectrum **21S-E6**

4-(2-(hexylamino)-1-hydroxyethyl)phenol (12S-B2)

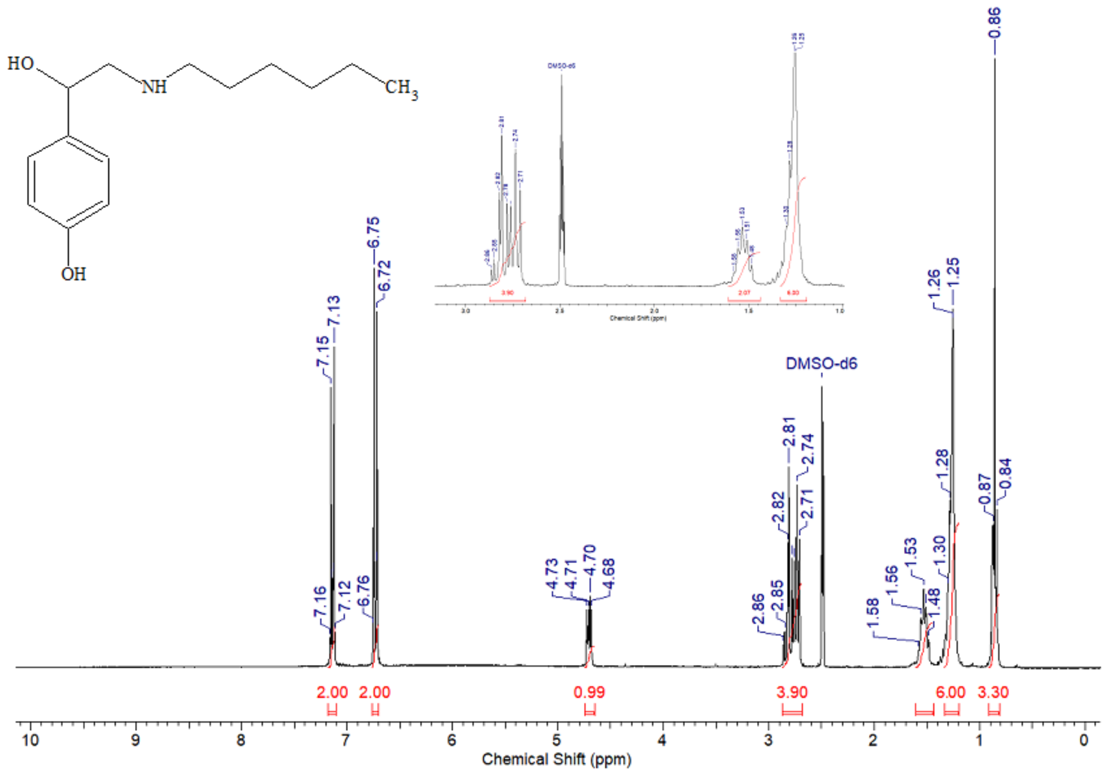

Figure S25. ^{1}H NMR spectrum **12S-B2**

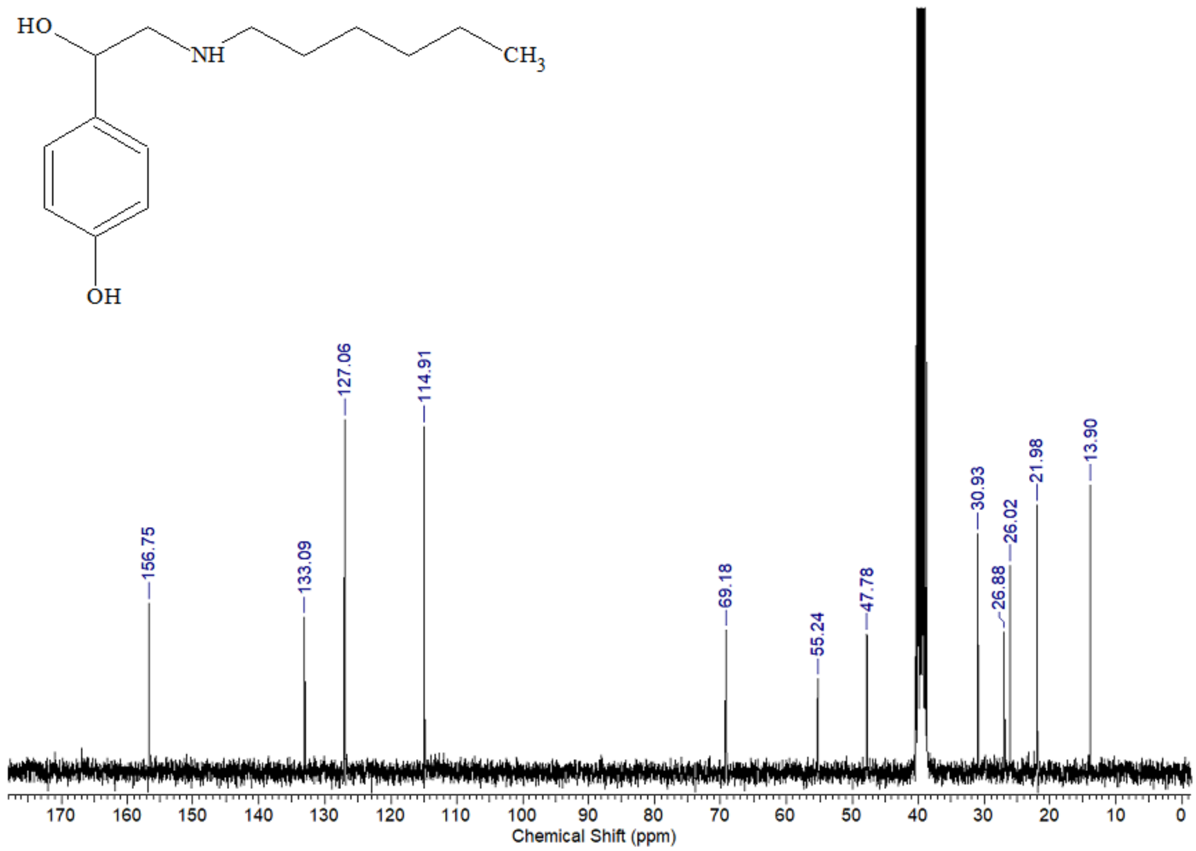

Figure S26. ^{13}C NMR spectrum **12S-B2**

4-(1-hydroxy-2-((2-hydroxyethyl)amino)ethyl)phenol (2S-B3)

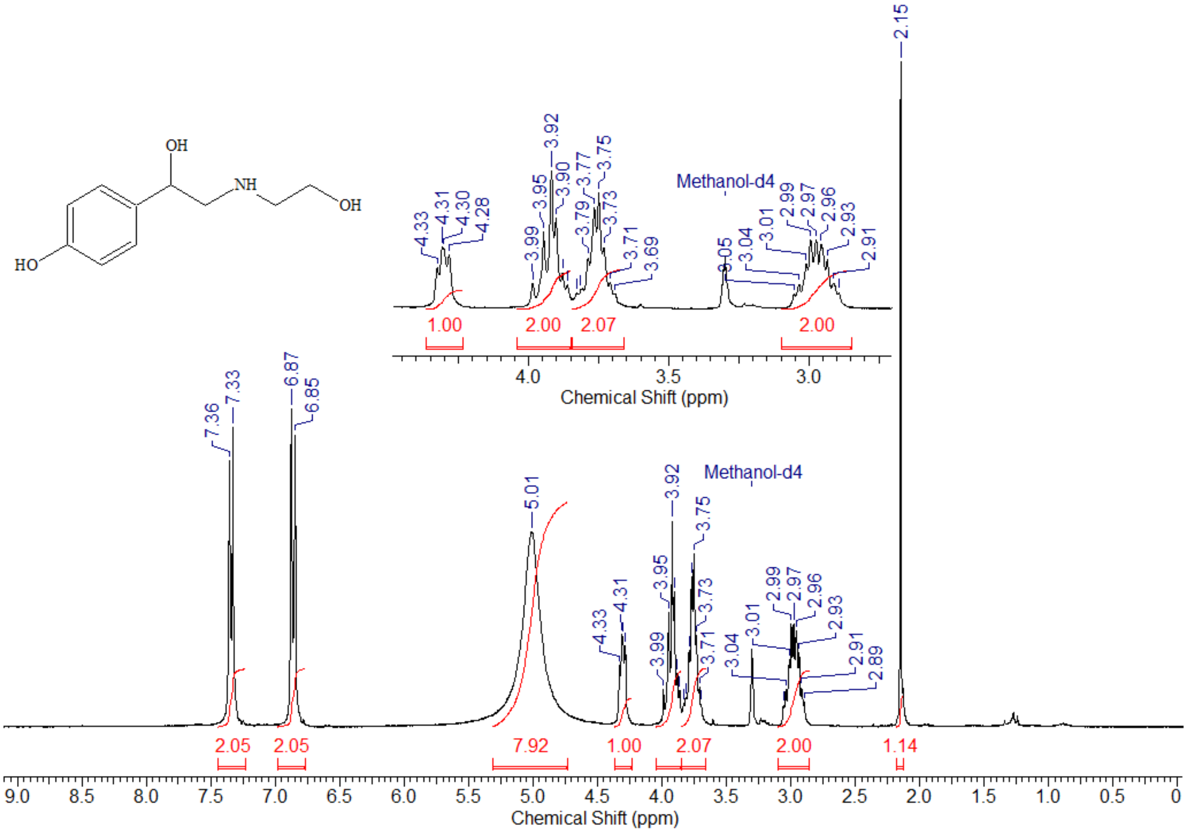

Figure S27. ^{1}H NMR spectrum **2S-B3**

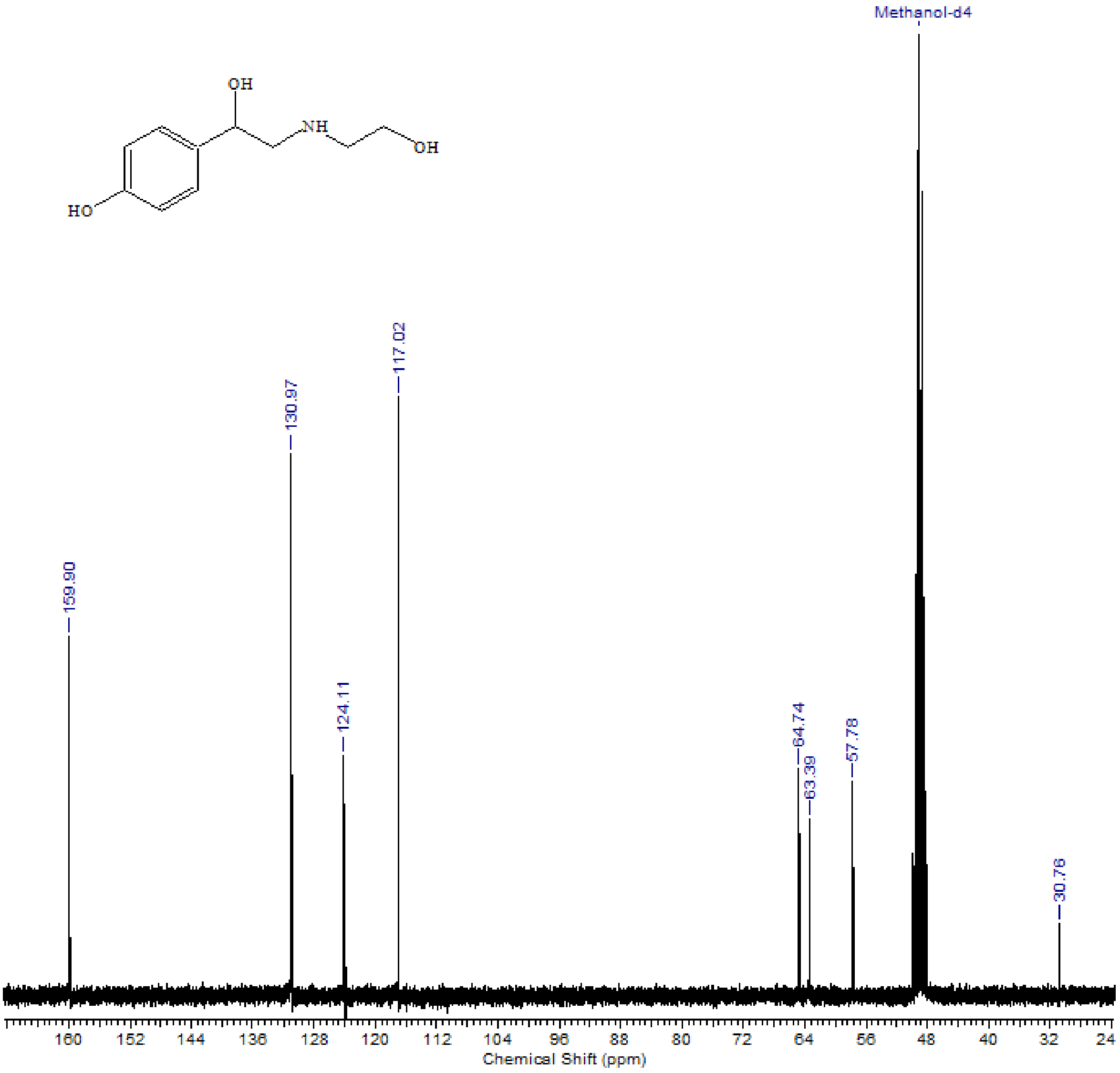

Figure S28. ^{13}C NMR spectrum **2S-B3**

2-((2-hydroxy-2-(4-hydroxyphenyl)ethyl)amino)propane-1,3-diol
(27S-B5)

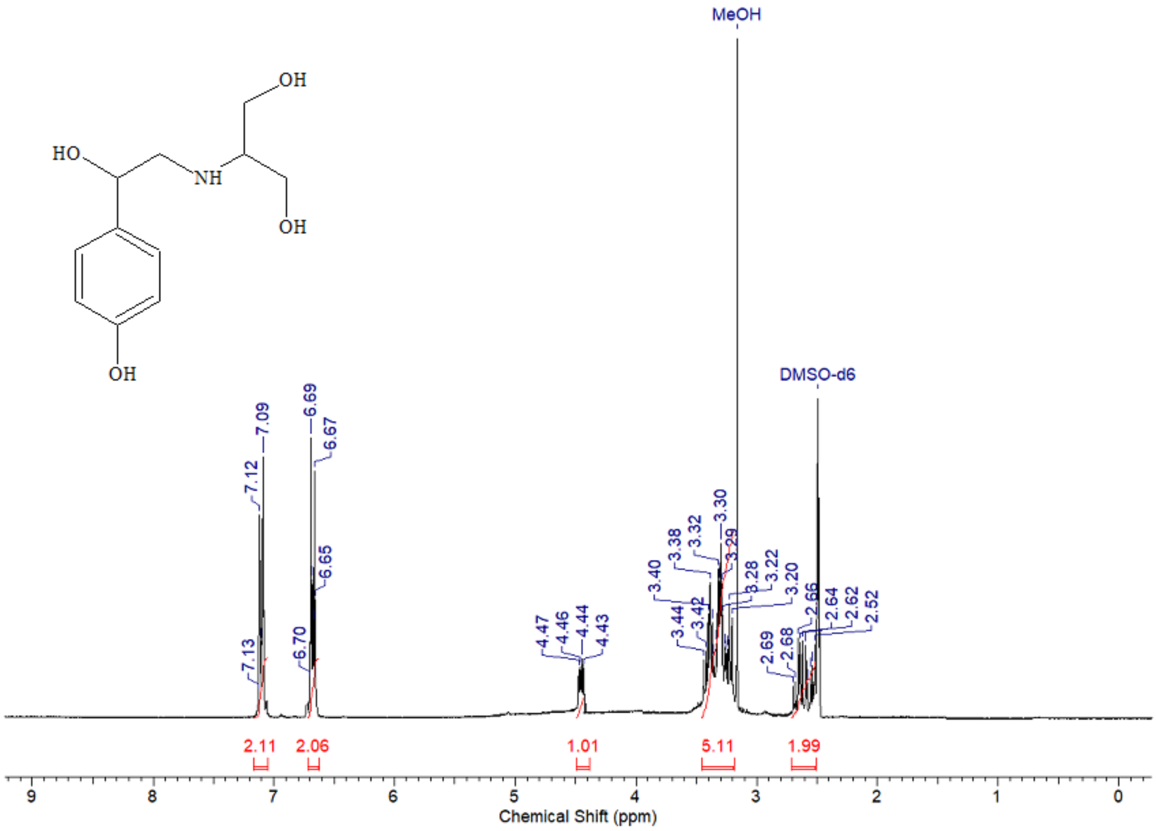

Figure S29. ^{1}H NMR spectrum **27S-B5**

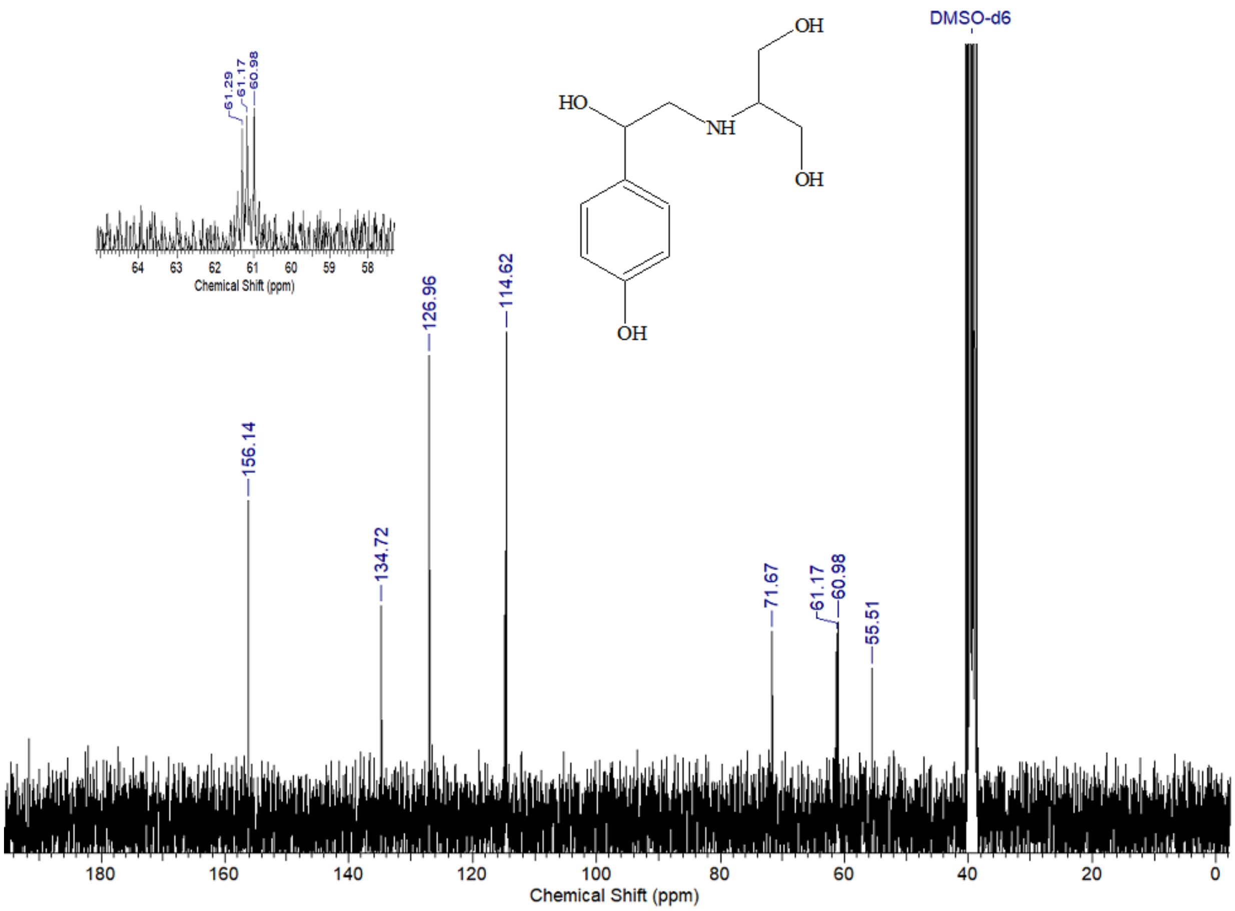

Figure S30. ^{13}C NMR spectrum **27S-B5**

3-((2-hydroxy-2-(4-hydroxyphenyl)ethyl)amino)propane-1,2-diol (17S-B6)

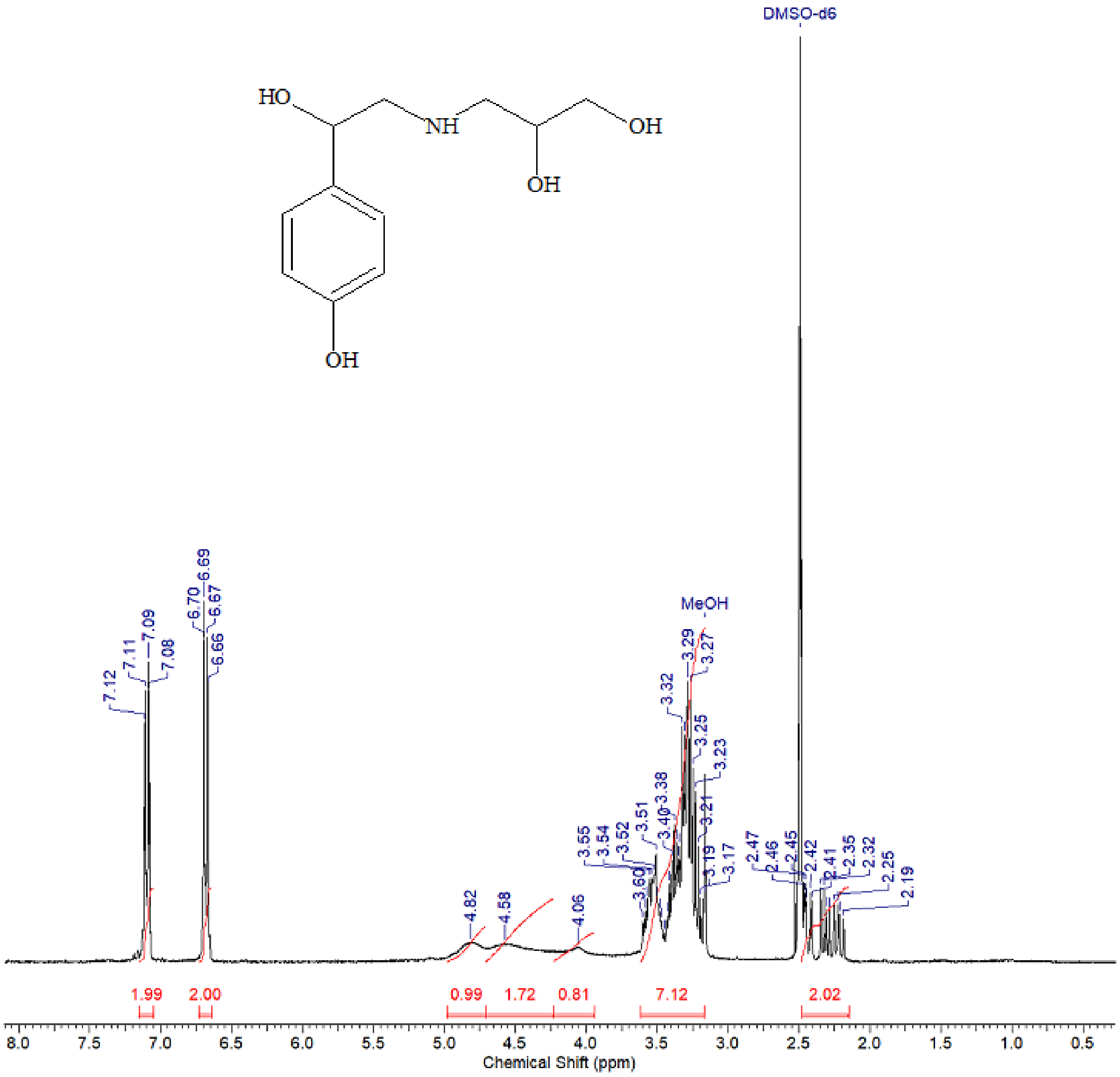

Figure S31. ^{1}H NMR spectrum **17S-B6**

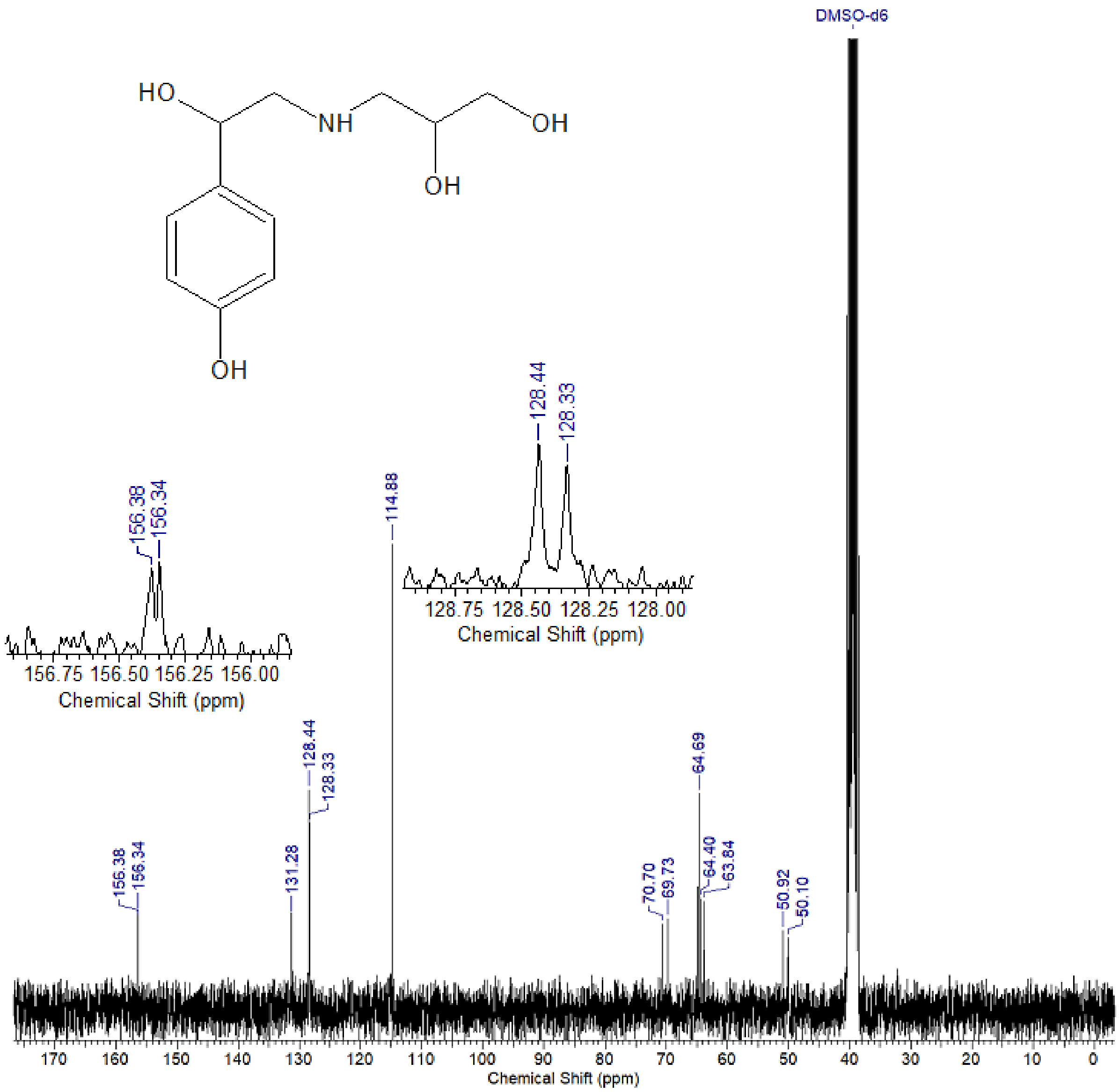

Figure S32. ^{13}C NMR spectrum **17S-B6**

2-(hexylamino)-1-(4-nitrophenyl)ethanol (13S-G2)

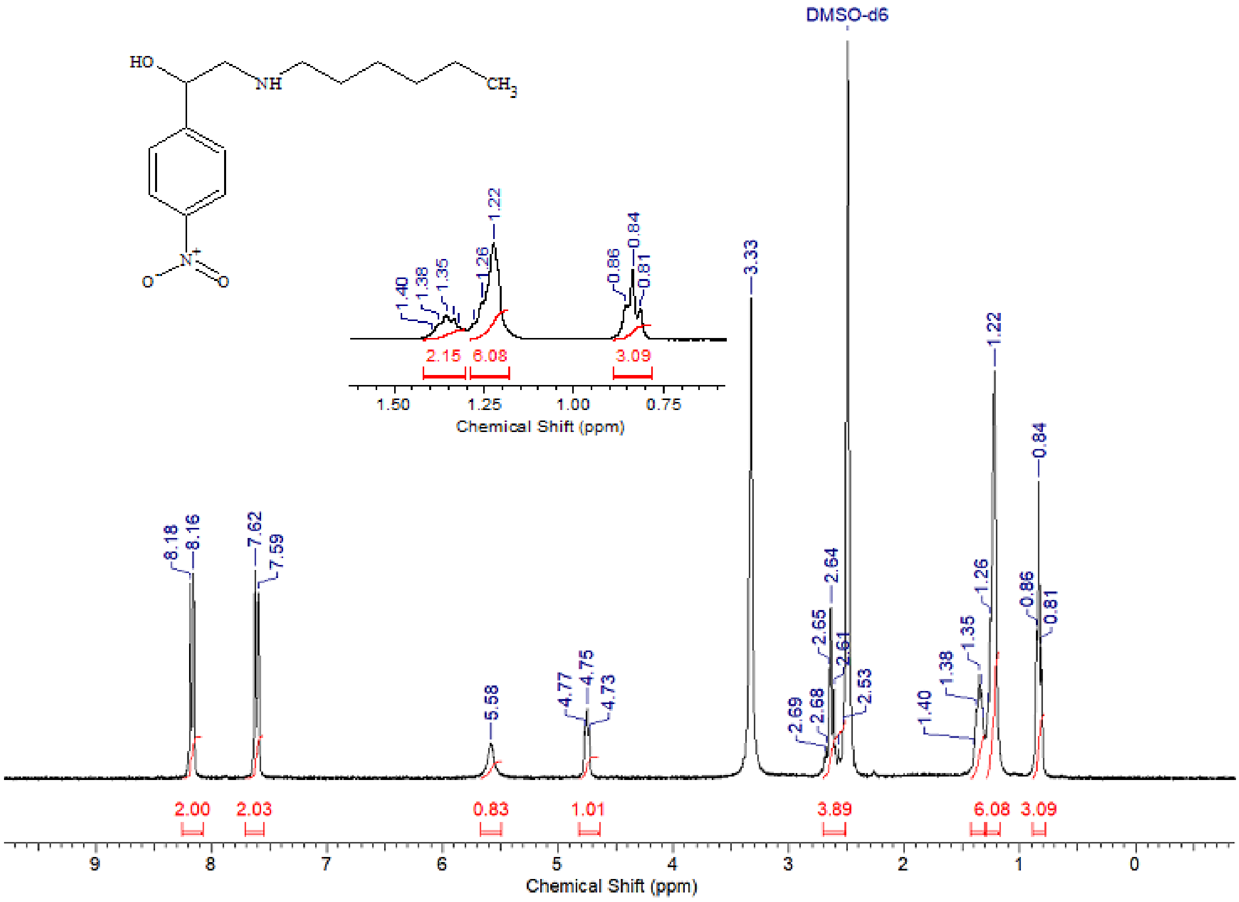

Figure S33. ^{1}H NMR spectrum **13S-G2**

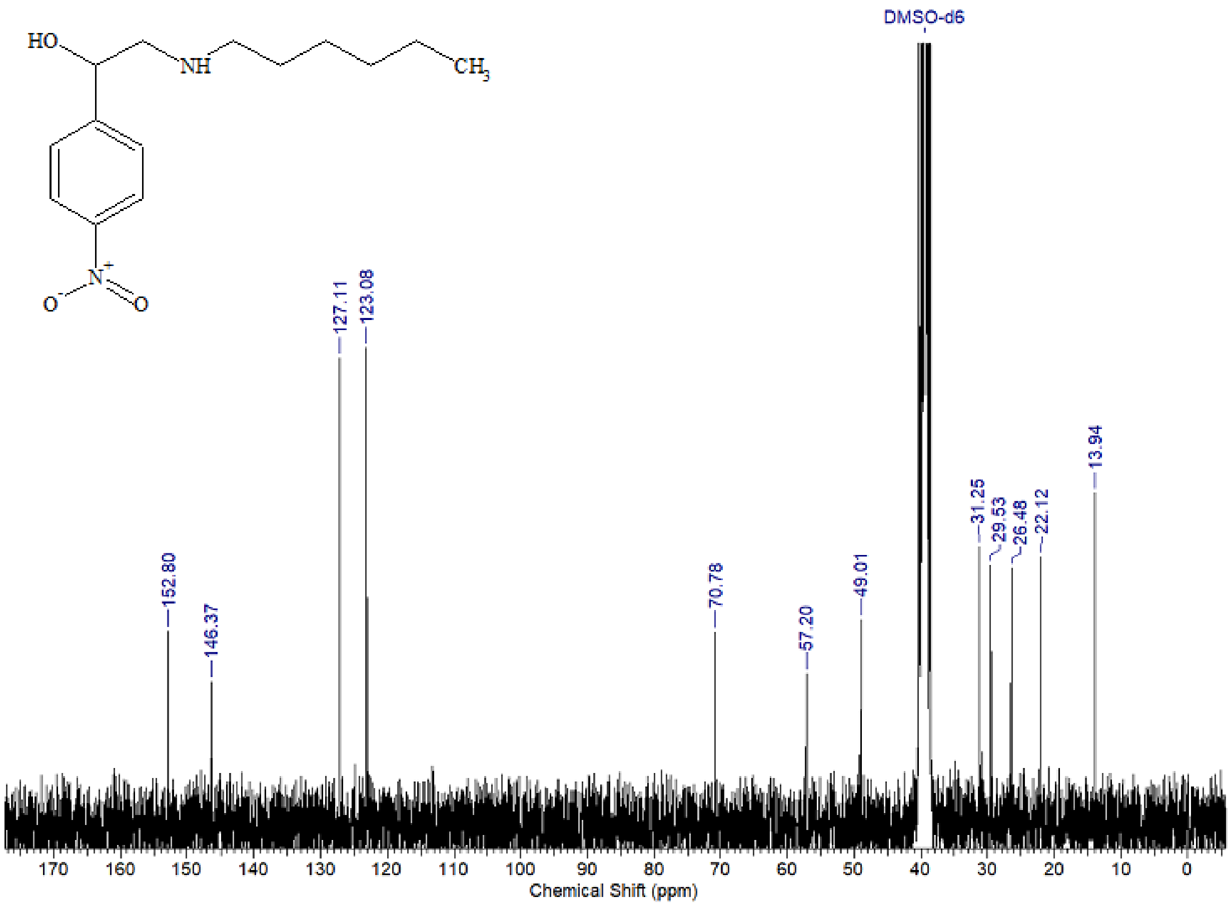

Figure S34. ^{13}C NMR spectrum **13S-G2**

2-((2-hydroxyethyl)amino)-1-(4-nitrophenyl)ethanol (14S-G3)

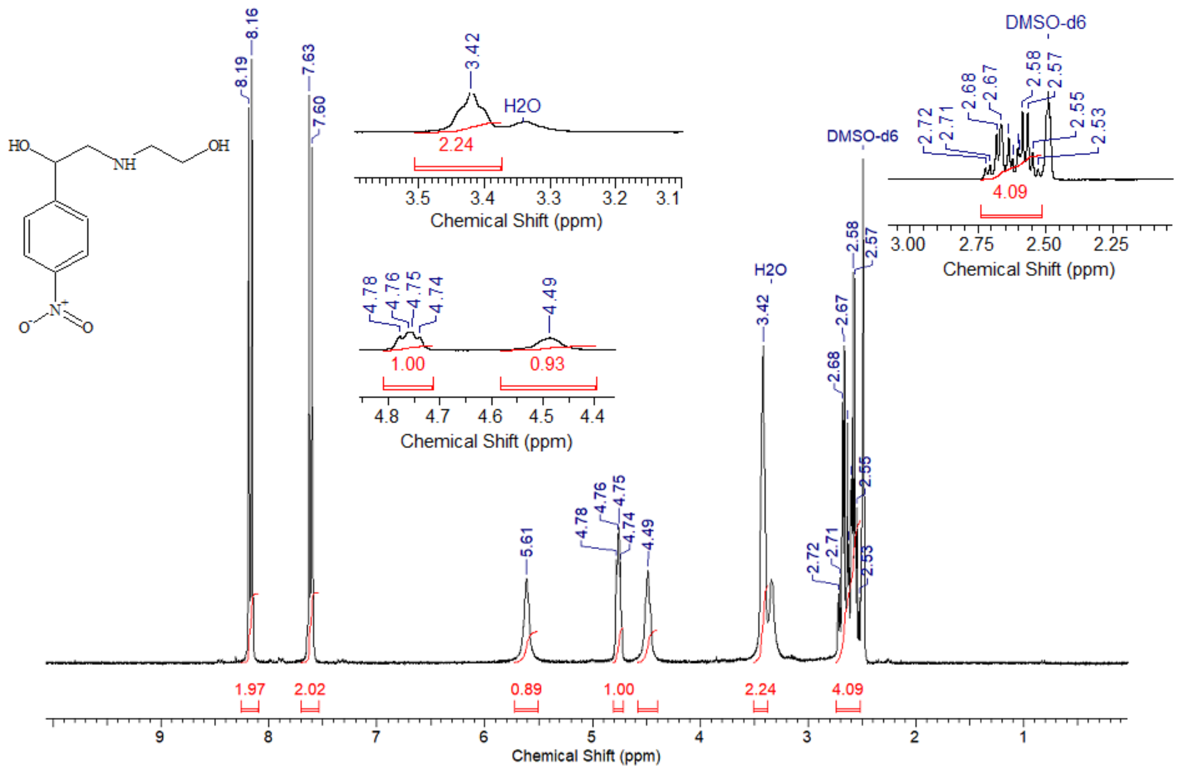

Figure S35. ^{1}H NMR spectrum **14S-G3**

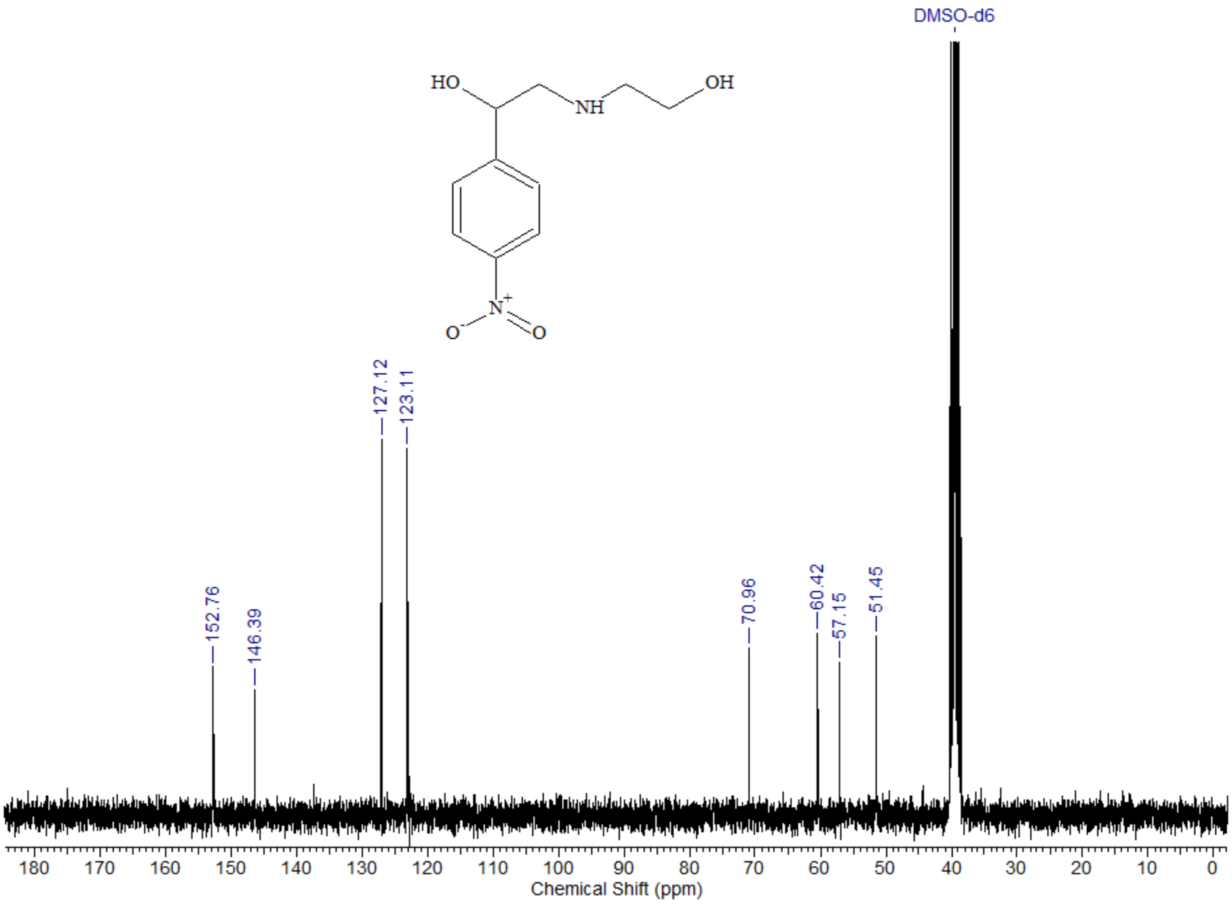

Figure S36. ^{13}C NMR spectrum **14S-G3**

2-((2-hydroxy-2-(4-nitrophenyl)ethyl)amino)propane-1,3-diol (22S-G5)

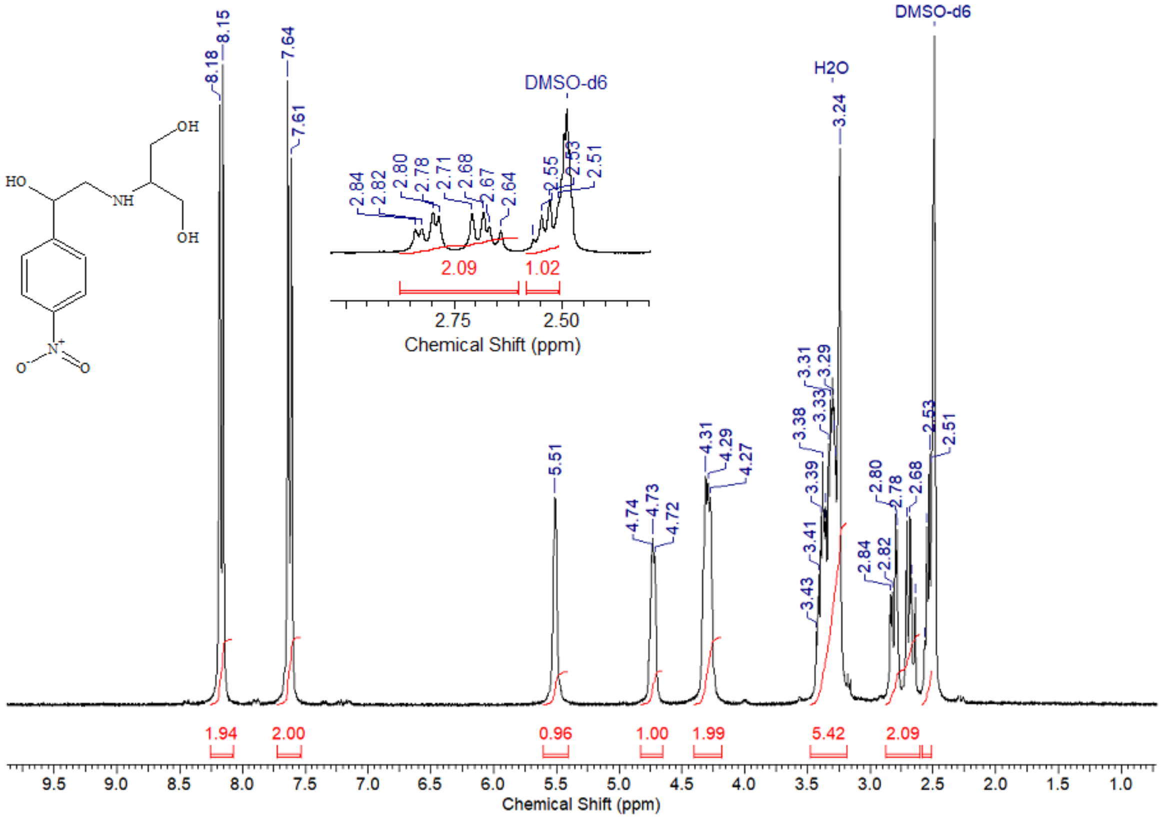

Figure S37. ^{1}H NMR spectrum **22S-G5**

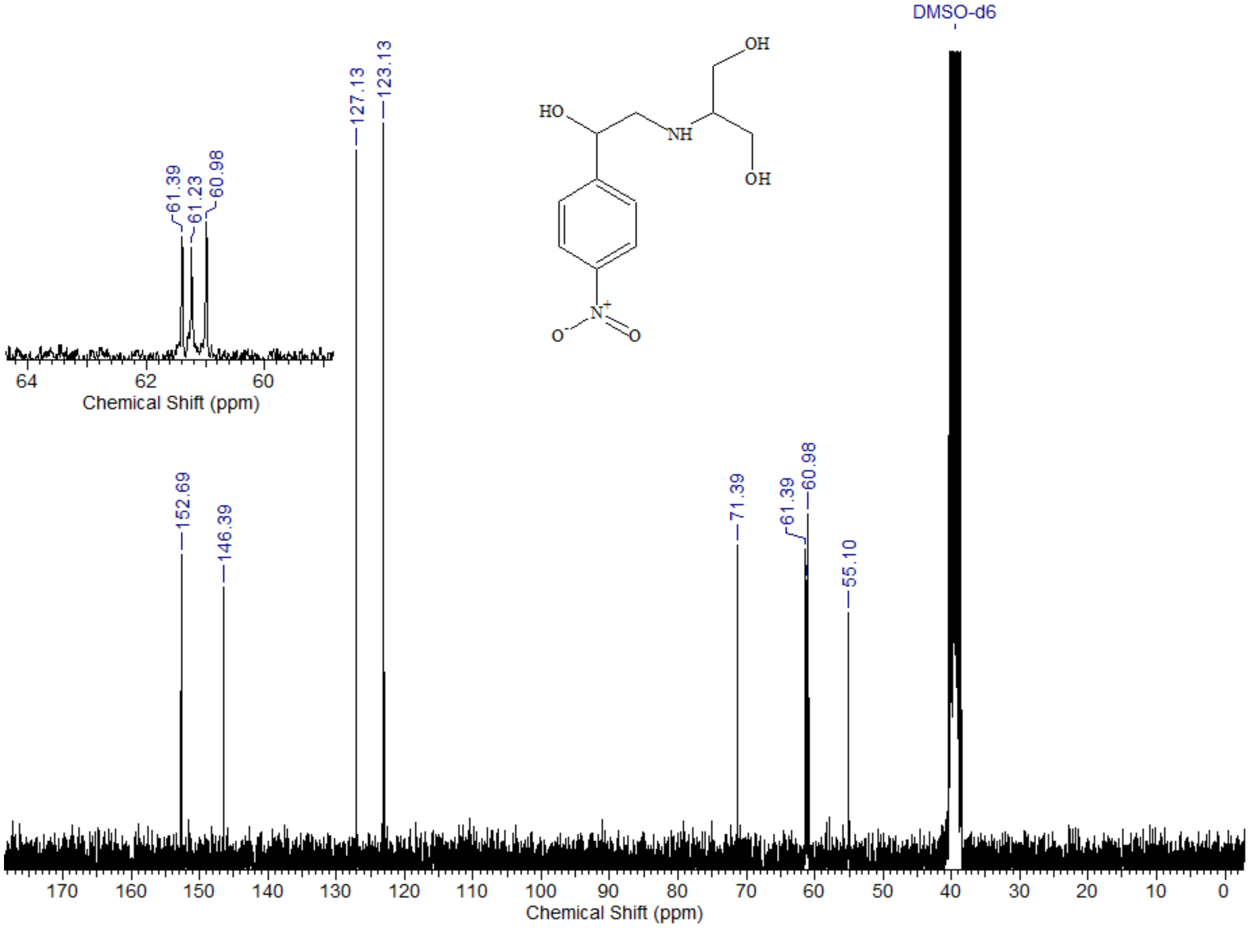

Figure S38. ^{13}C NMR spectrum **22S-G5**

3-((2-hydroxy-2-(4-nitrophenyl)ethyl)amino)propane-1,2-diol (23S-G6)

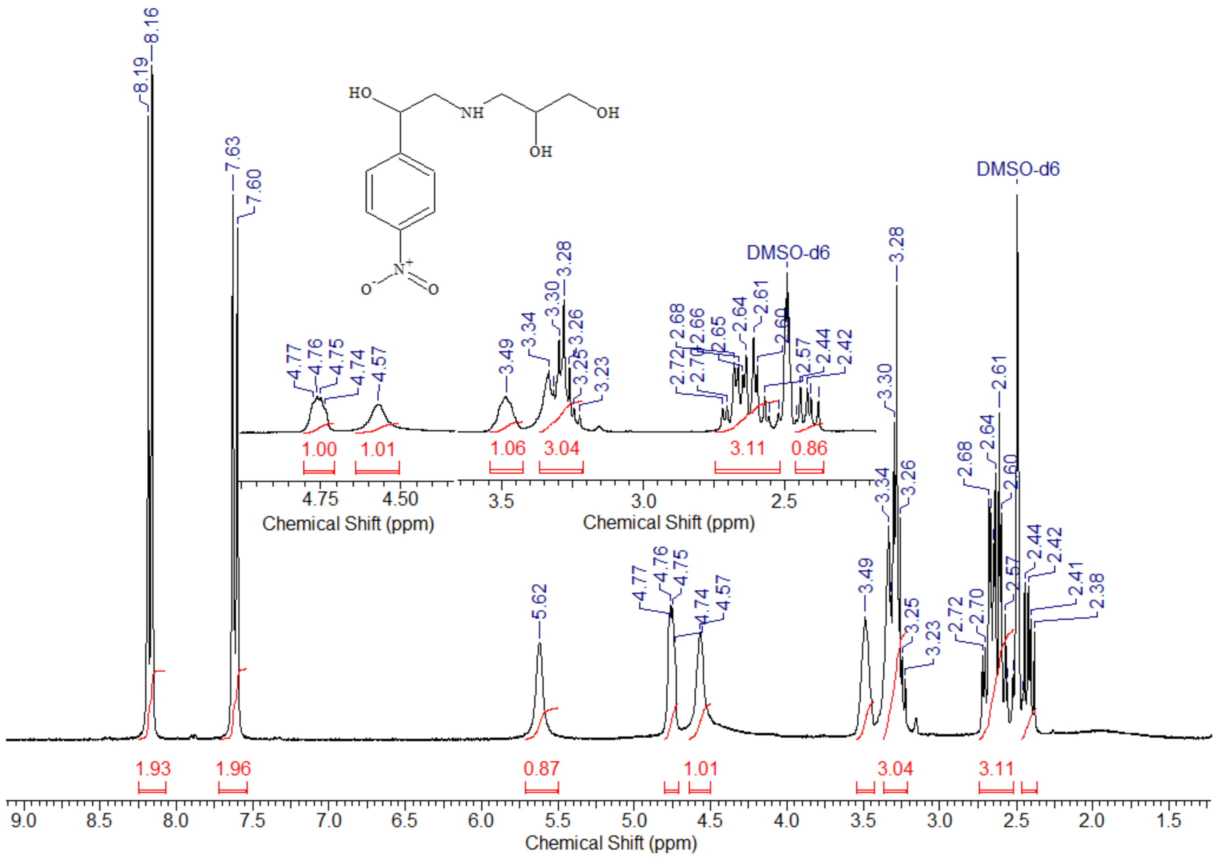

Figure S39. ^{1}H NMR spectrum **23S-G6**

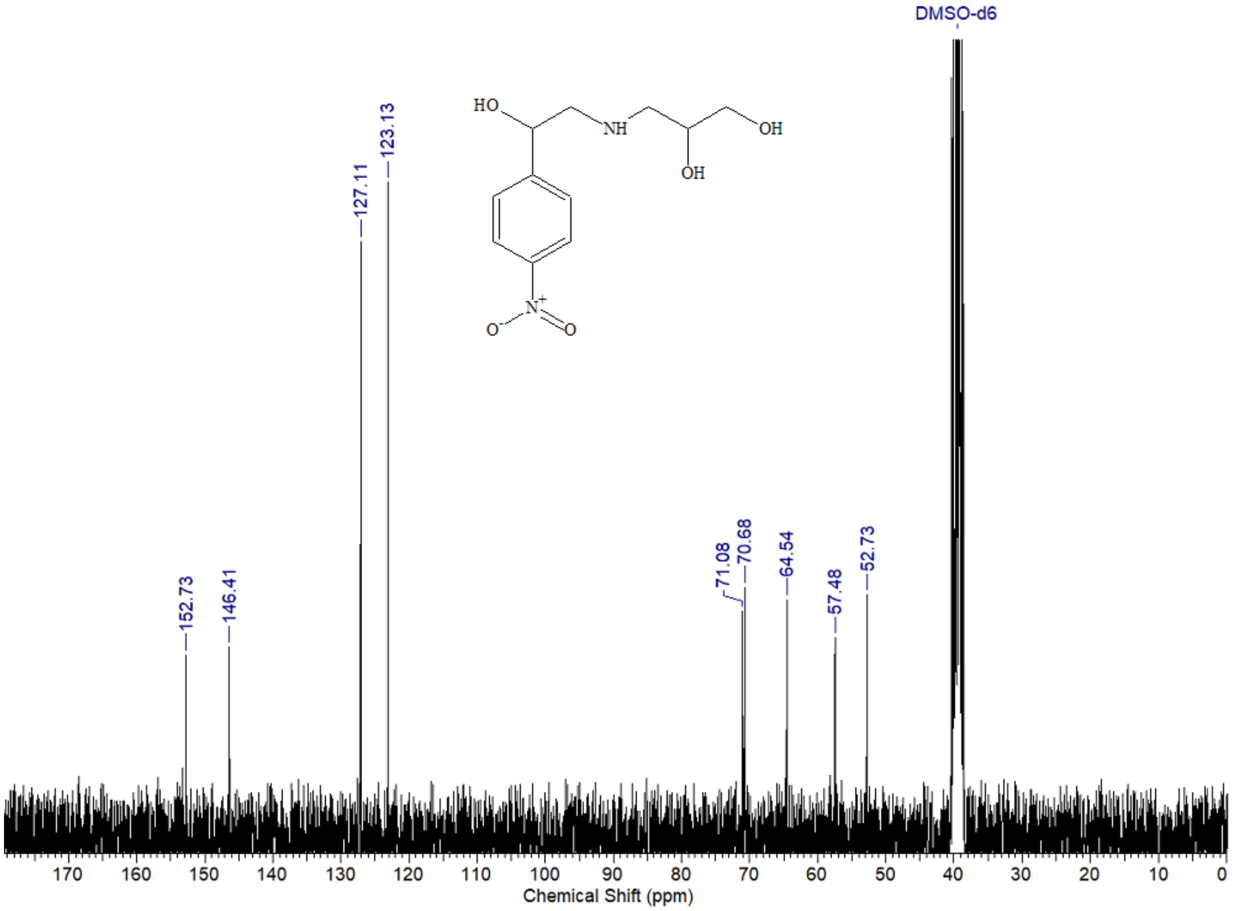

Figure S40. ^{13}C NMR spectrum **23S-G6**

1-(4-nitrophenyl)-2-piperidin-1-yl ethanol (9S-G1)

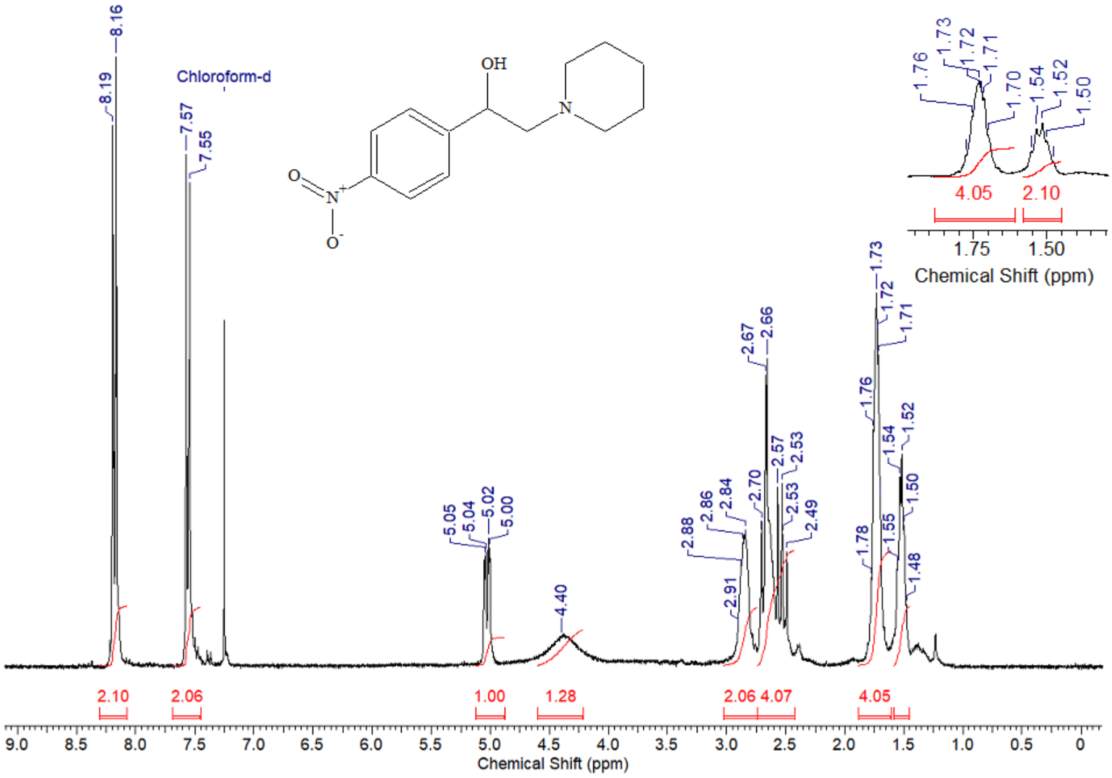

Figure S41. ^{1}H NMR spectrum **9S-G1**

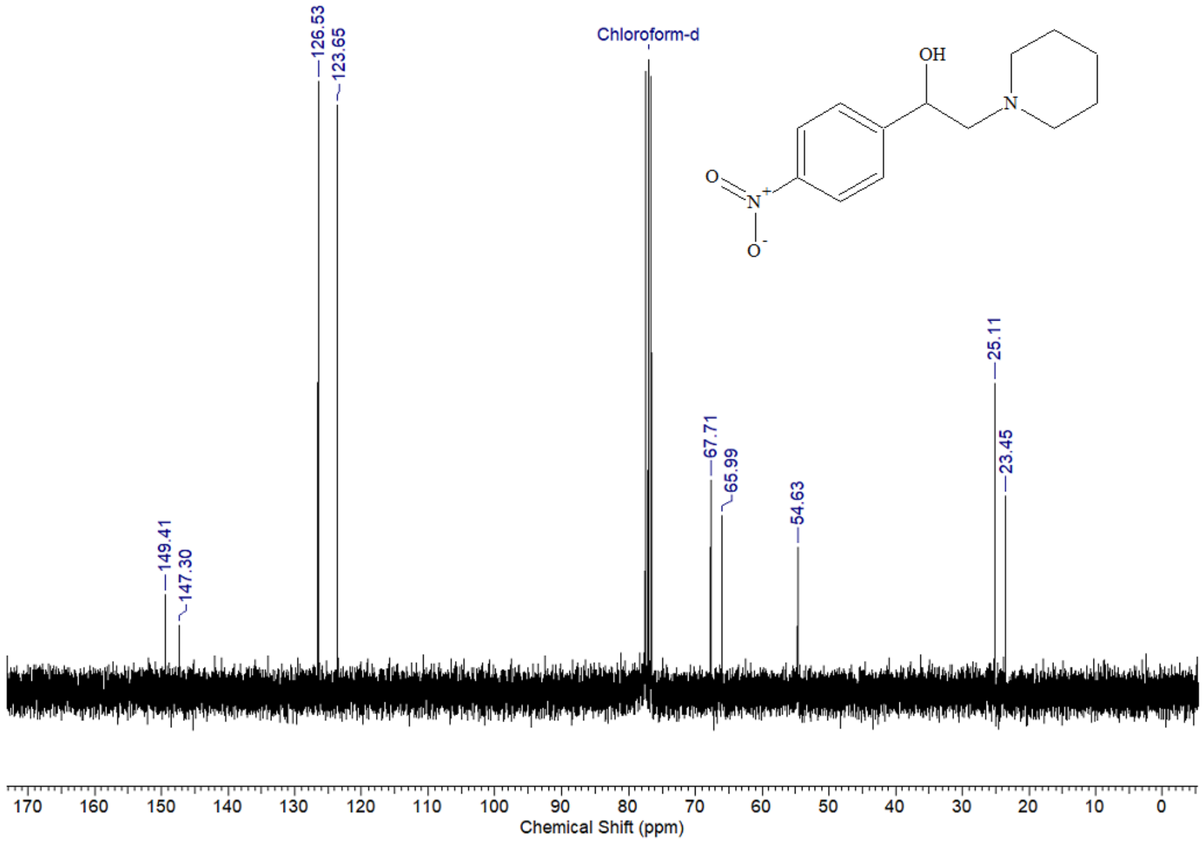

Figure S42. ^{13}C NMR spectrum **9S-G1**

1-(4-aminophenyl)-2-(hexylamino)ethanol (26S-F2)

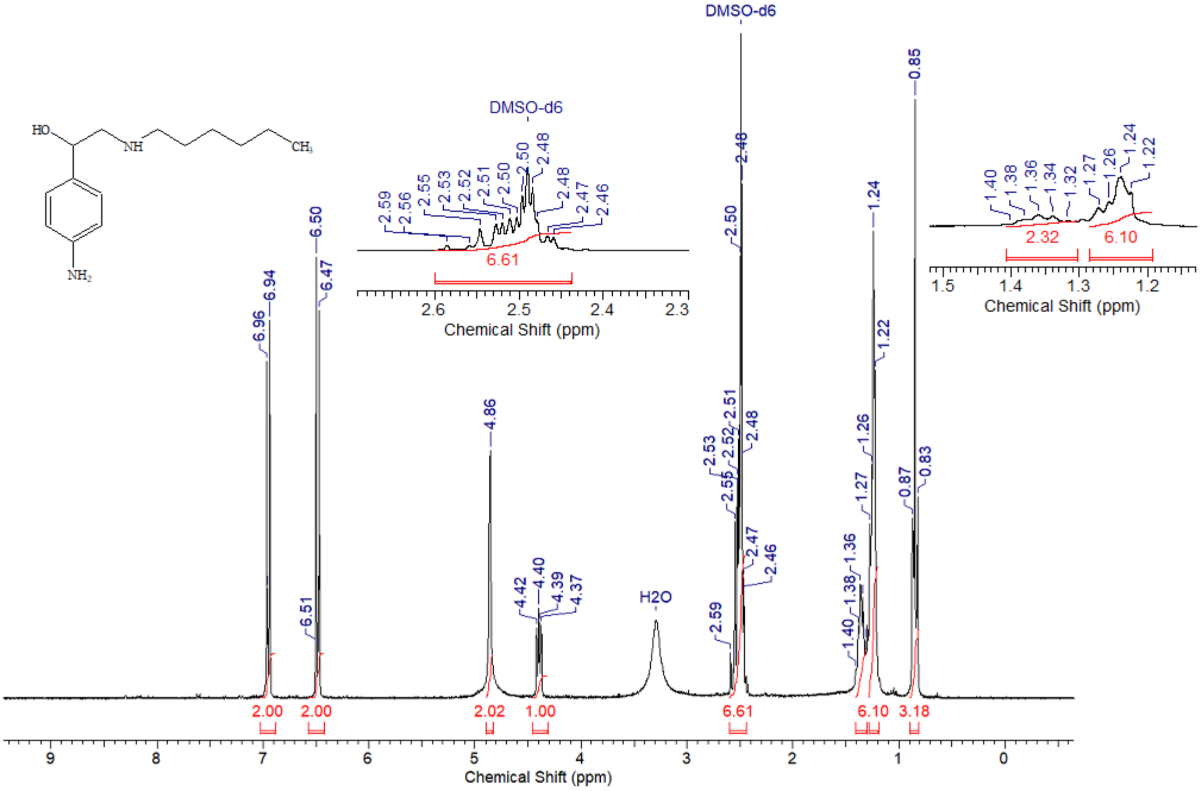

Figure S43. ^{1}H NMR spectrum **26S-F2**

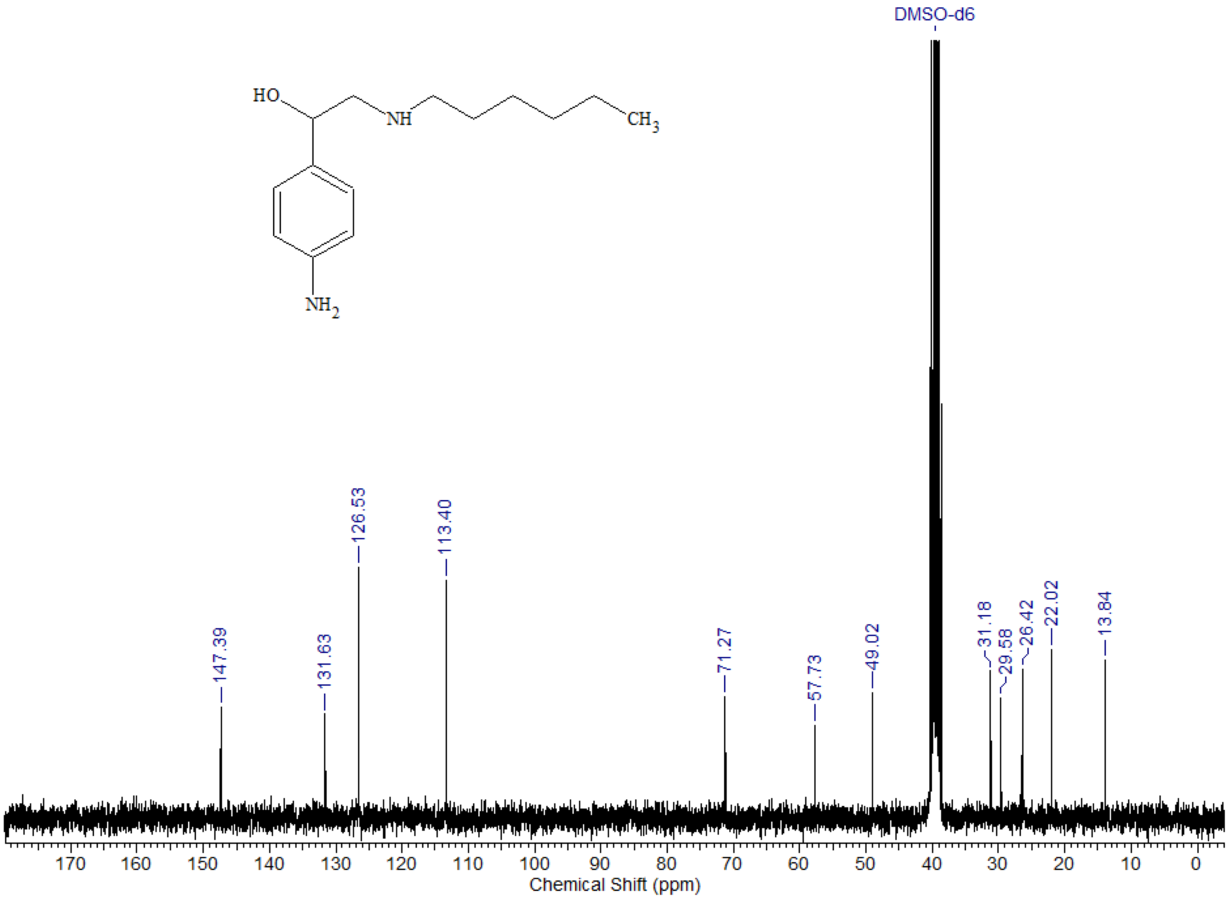

Figure S44. ^{13}C NMR spectrum **26S-F2**

1-(4-aminophenyl)-2-((2-hydroxyethyl)amino)ethanol (16S-F3)

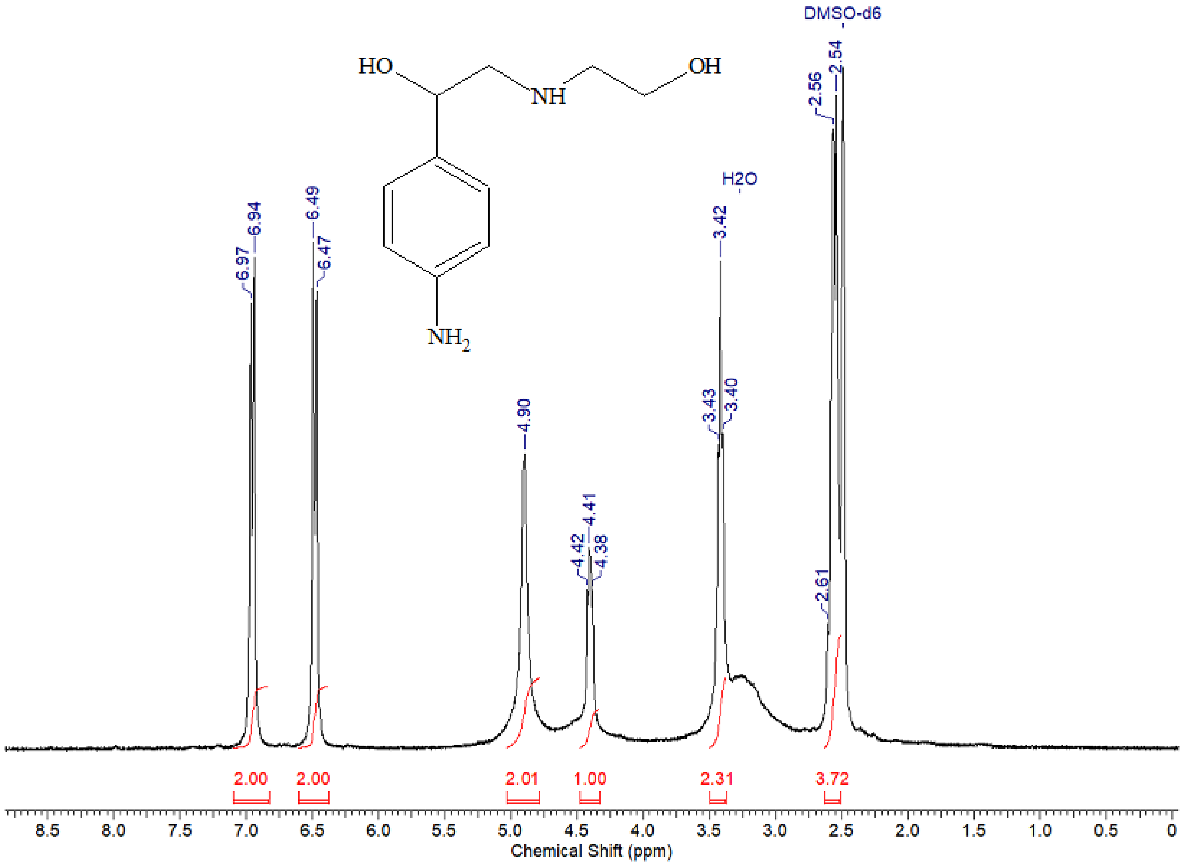

Figure S45. ^{1}H NMR spectrum **16S-F3**

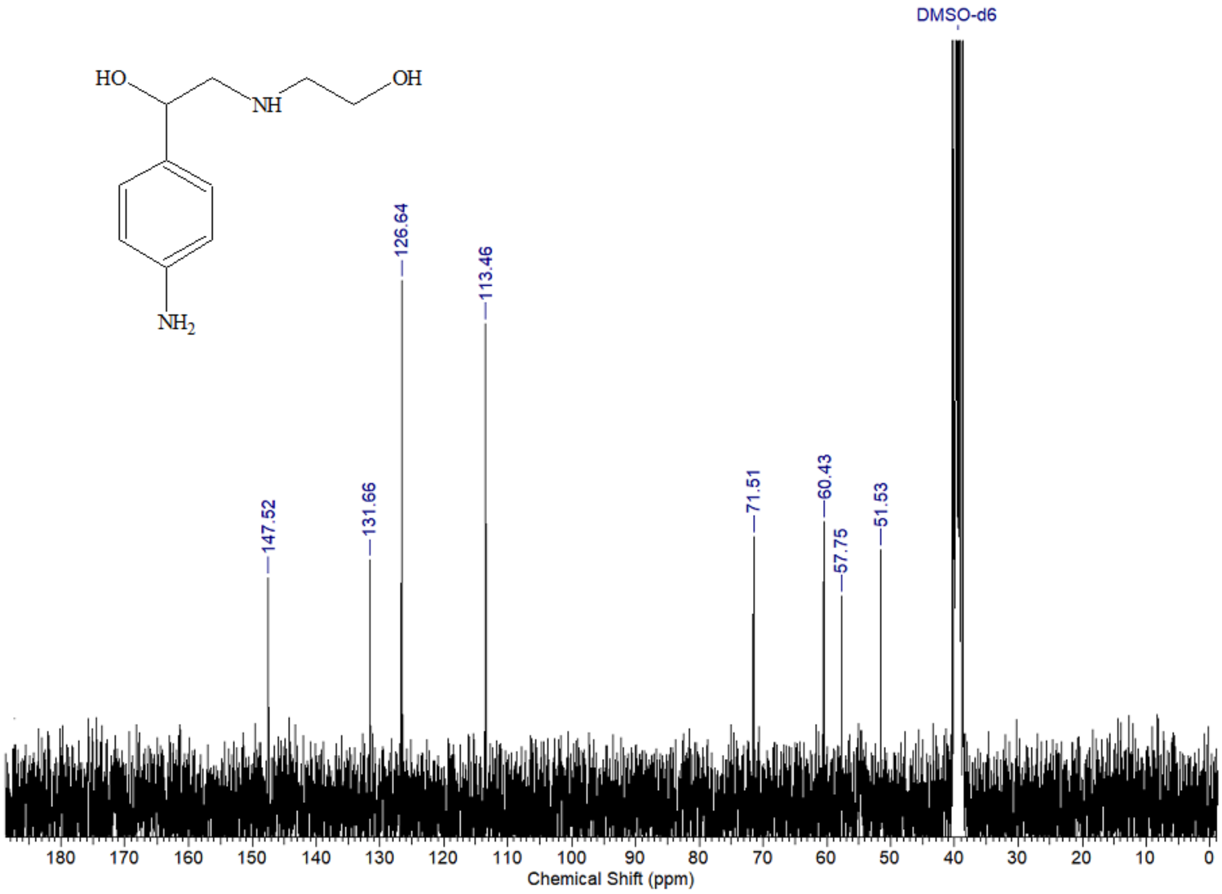

Figure S46. ^{13}C NMR spectrum **16S-F3**

2-((2-(4-aminophenyl)-2-hydroxyethyl)amino)propane-1,3-diol (24S-F5)

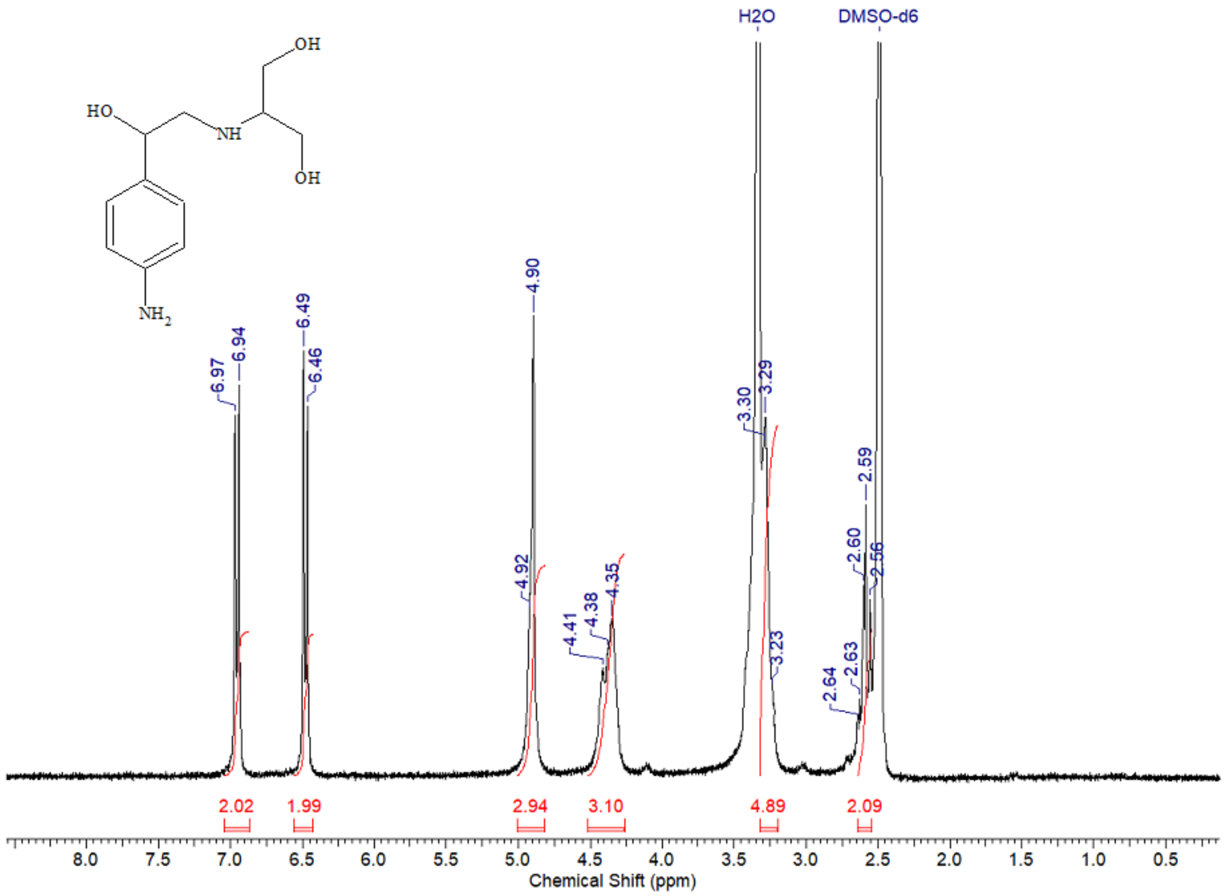

Figure S47. ^{1}H NMR spectrum **24S-F5**

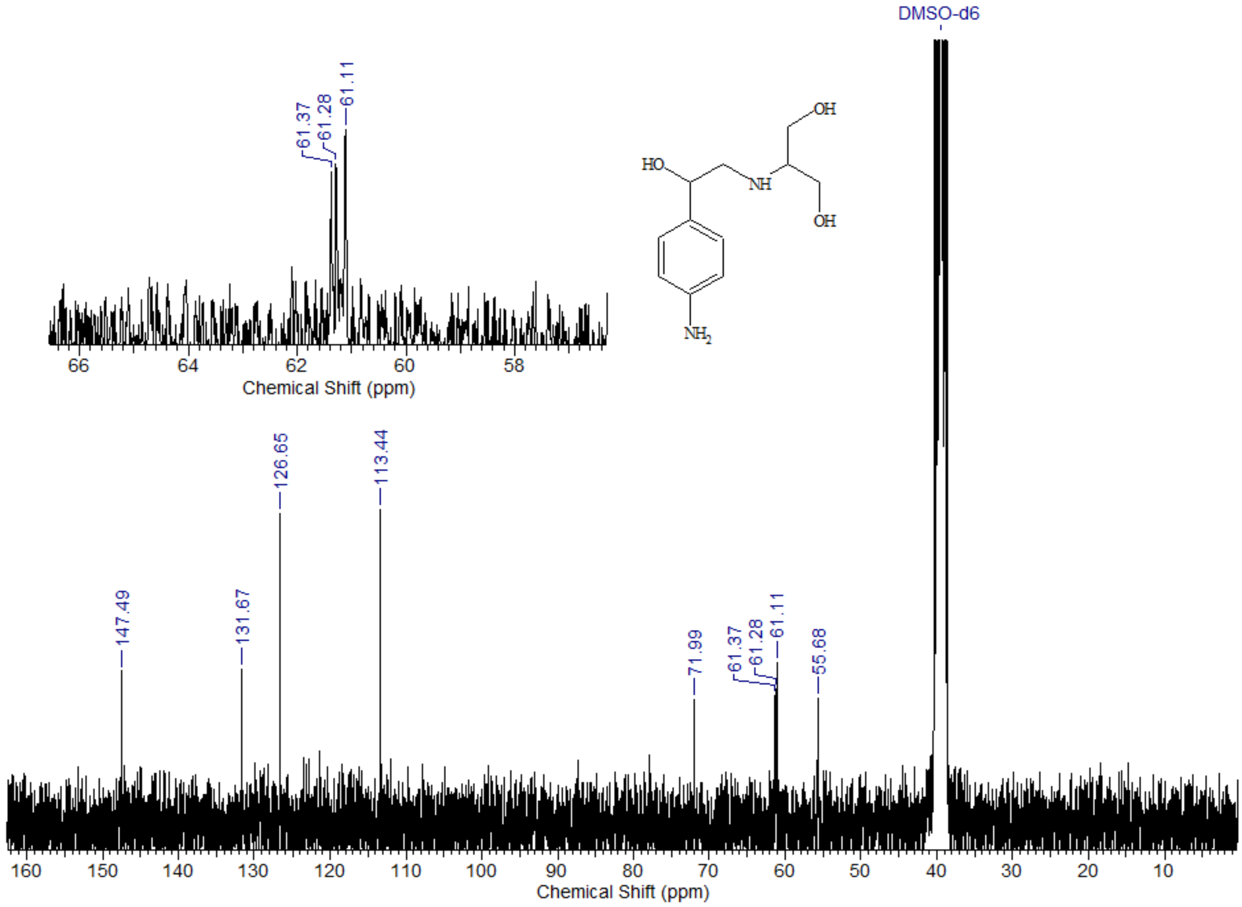

Figure S48. ^{13}C NMR spectrum **24S-F5**

3-((2-(4-aminophenyl)-2-hydroxyethyl)amino)propane-1,2-diol (25S-F6)

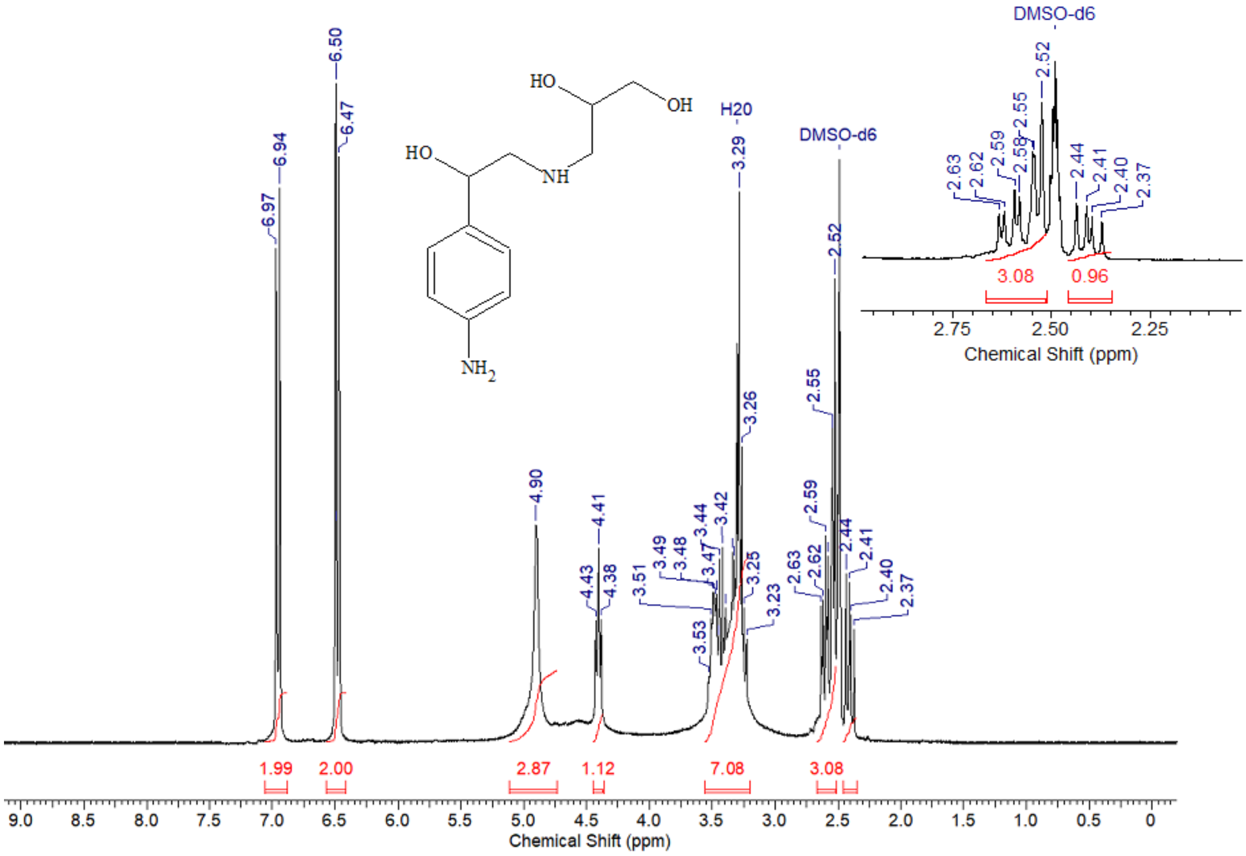

Figure S49. ^{1}H NMR spectrum **25S-F6**

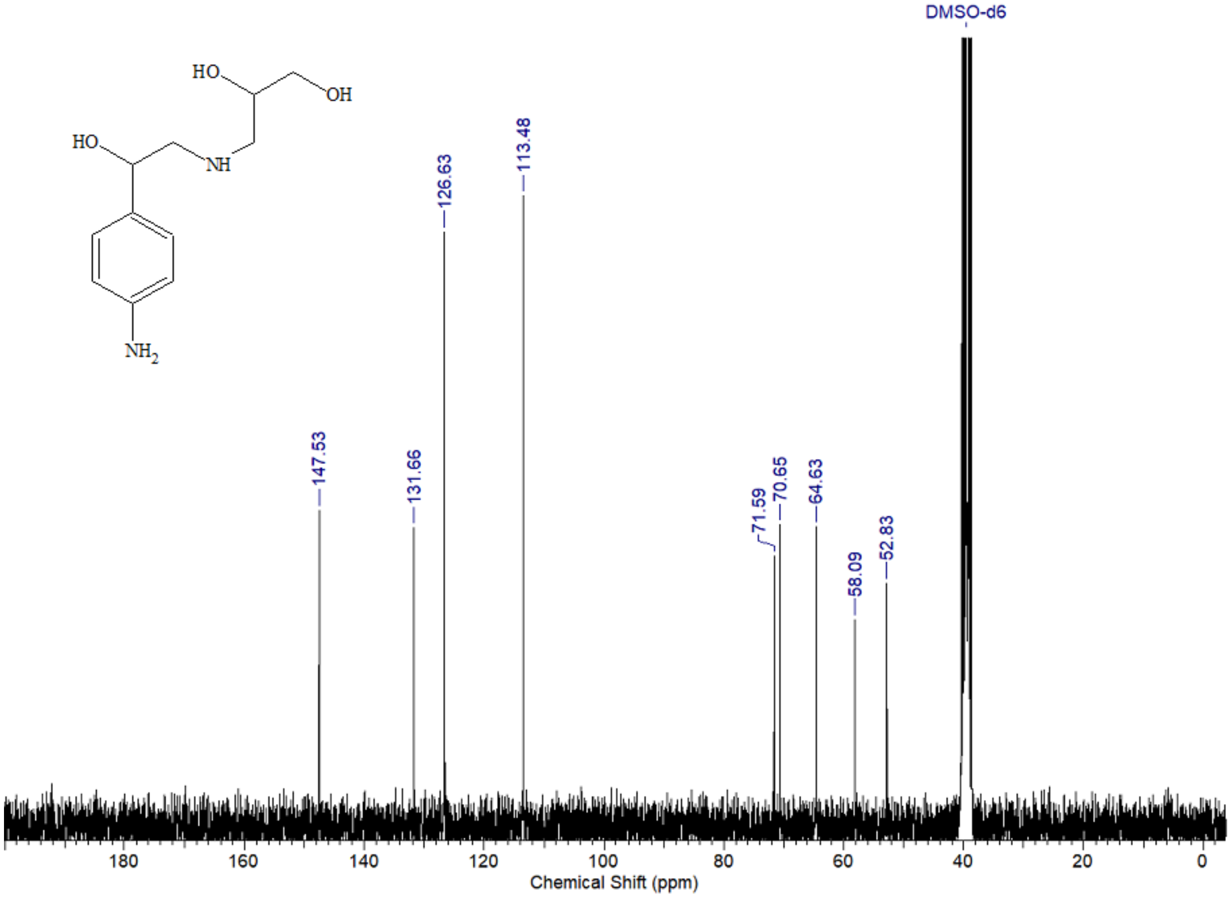

Figure S50. ^{13}C NMR spectrum **25S-F6**

1-(4-aminophenyl)-2-piperidin-1-ylethanol (15S-F1)

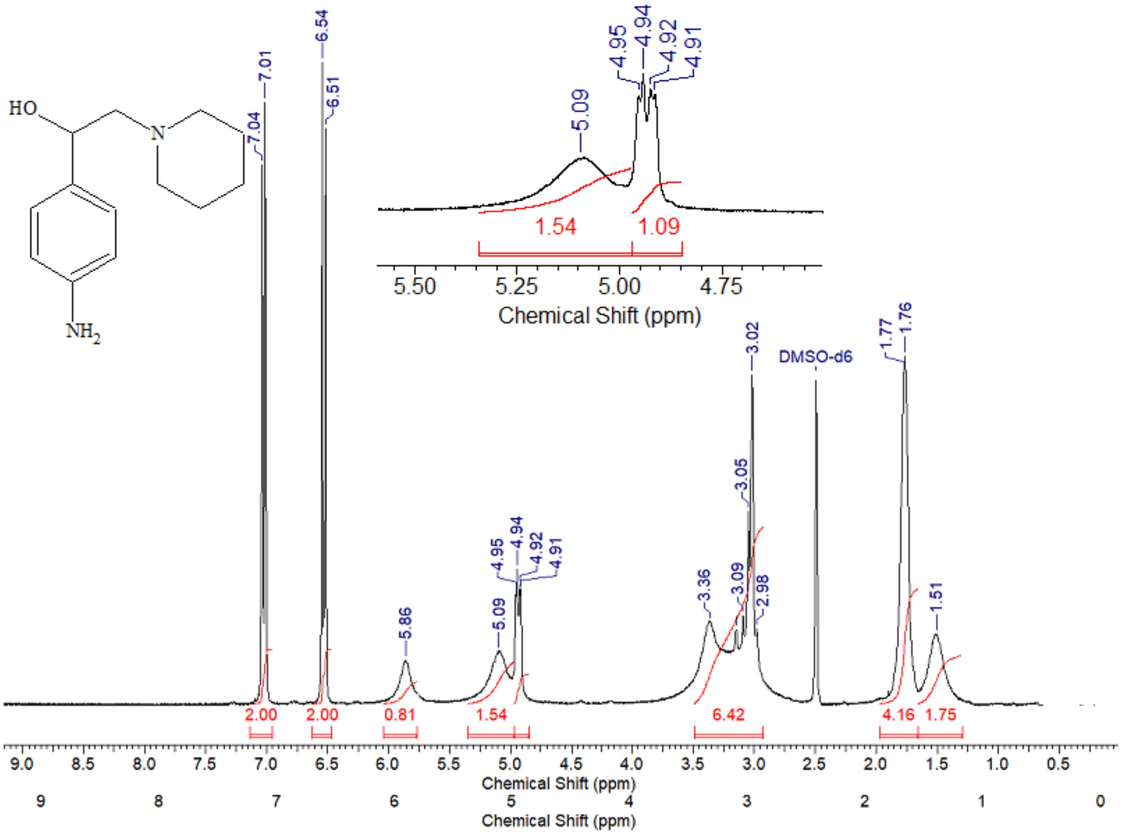

Figure S51. ¹H NMR spectrum **15S-F1**

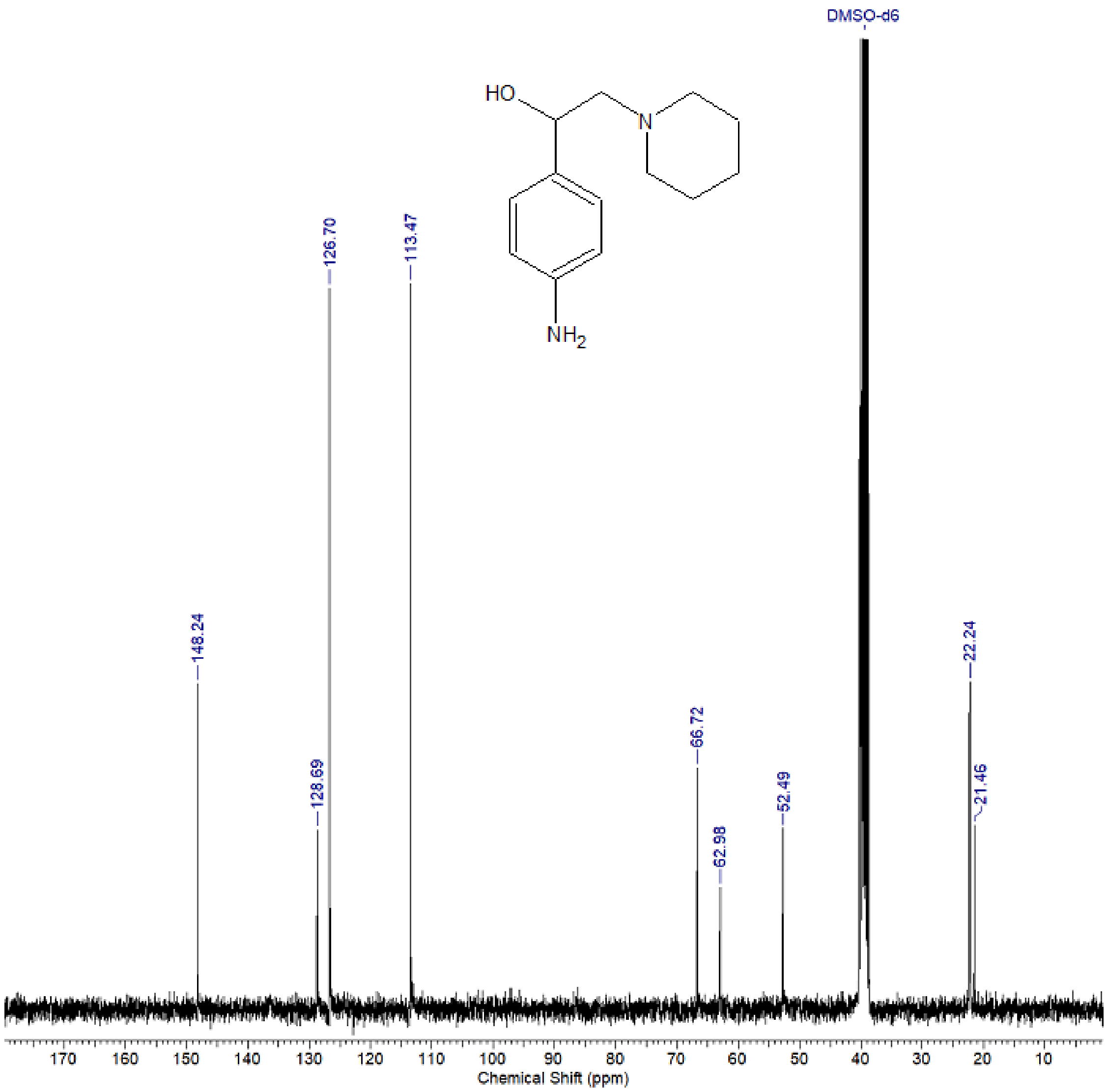

Figure S52. ^{13}C NMR spectrum **15S-F1**

Dexamethasone

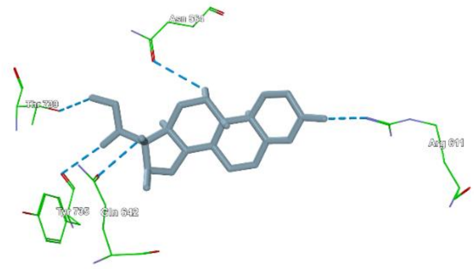

Synephrine

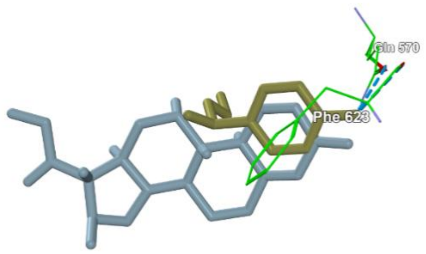

4S-C2

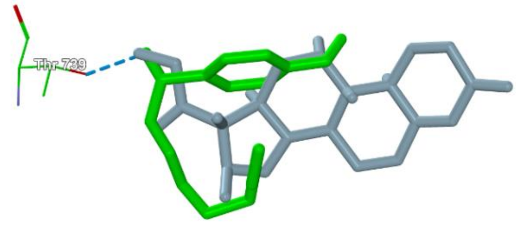

8S-E3

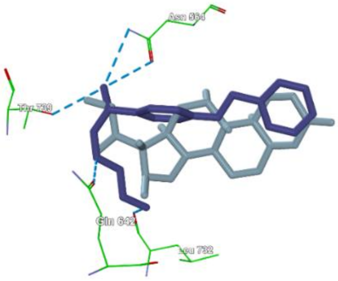

10S-C2

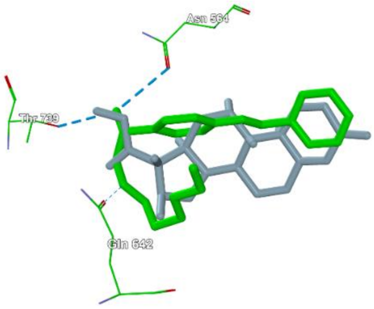

12S-B2

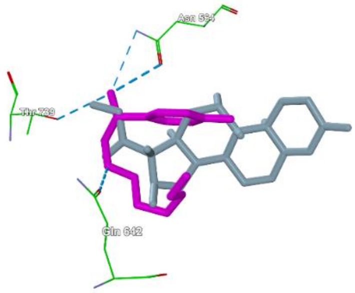

13S-G2

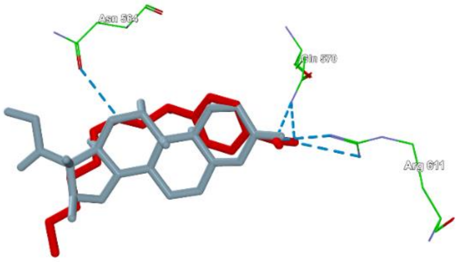

20S-E5

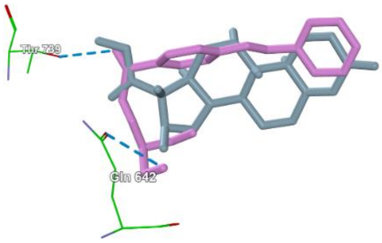

21S-E6

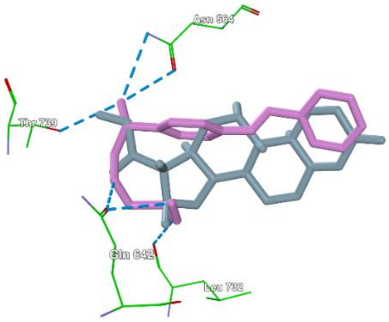

26S-F2

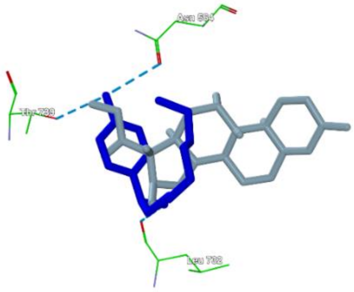

Figure S53. Structure of the GR active site with analogue of synephrine

Table S2. Calculated lipophilicity of structures (calculated using a software package ACD/Labs 6.00), structures that have demonstrated biological activity are highlighted in green.

	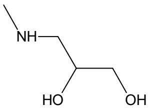	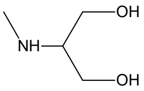	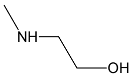	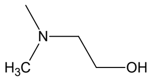	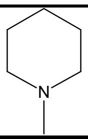	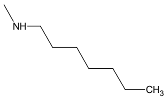
$-NH_2$	**25S-F6**<br>-1.73±0.39	**24S-F5**<br>-1.21±0.58	**16S-F3**<br>-1.11±0.31	**-**	**15S-F1**<br>1.37±0.46	**26S-F2**<br>2.08±0.27
-OH	**17S-B6**<br>-1.19±0.39	**27S-B5**<br>-0.67±0.58	**2S-B3**<br>-0.57±0.31	**-**	**-**	**12S-B2**<br>2.63±0.27
$-NO_2$	**23S-G6**<br>-0.72±0.39	**22S-G5**<br>-0.20±0.58	**14S-G3**<br>-0.10±0.32	**-**	**9S-G1**<br>2.38±0.47	**13S-G2**<br>3.09±0.27
-OMe	**19S-C6**<br>-0.54±0.39	**18S-C5**<br>-0.01±0.58	**5S-C3**<br>0.08±0.32	**6S-C4**<br>0.92±0.35	**3S-C1**<br>2.57±0.47	**4S-C2**<br>3.28±0.28
-OBn	**21S-E6**<br>1.12±0.40	**20S-E5**<br>1.64±0.59	**8S-E3**<br>1.74±0.38	**11S-E4**<br>2.57±0.39	**7S-E1**<br>4.22±0.48	**10S-E2**<br>4.93±0.28
Dex			1.87±0.63			
